# Confinement-Driven Aggregate Formation of Photoacids within Porous Metal–Organic Frameworks

**DOI:** 10.1021/acsomega.4c09621

**Published:** 2025-01-30

**Authors:** Markus Rödl, Viktoria Kiefer, Selina Olthof, Klaus Meerholz, Gregor Jung, Heidi A. Schwartz

**Affiliations:** † Institute of General, Inorganic and Theoretical Chemistry, Universität Innsbruck, Innrain 80–82, A-6020 Innsbruck, Austria; ‡ Biophysical Chemistry, Saarland University, Campus, Building B2 2, D-66123 Saarbrücken, Germany; § Department of Chemistry, University of Cologne, Greinstraße 4–6, D-50939 Cologne, Germany

## Abstract

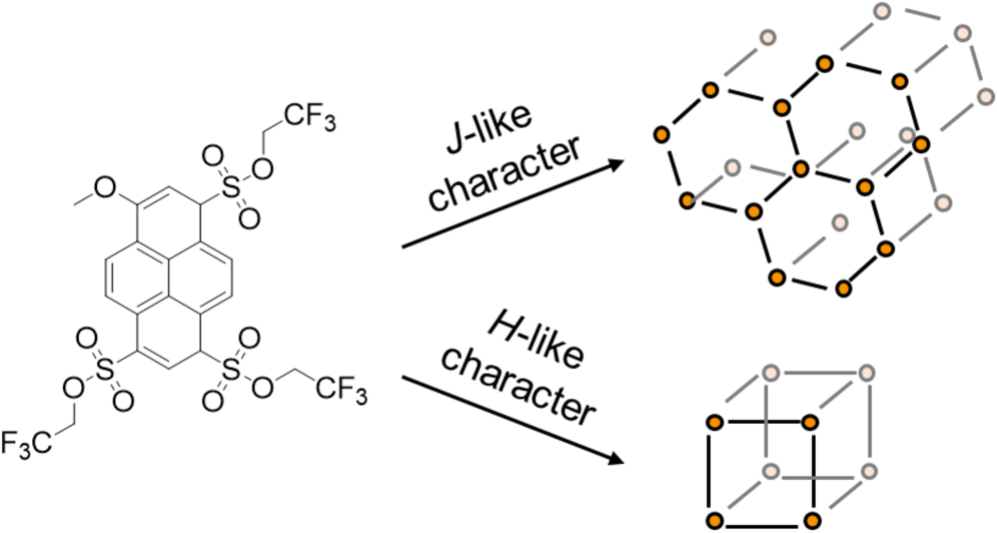

Structurally driven properties of hybrid materials are a fascinating feature of metal–organic framework (MOF) materials that can serve as hosts for various responsive dye molecules. In particular, the formation of aggregates and the related shift of the emission of fluorophors can be tuned as a function of pore confinement. In this work, the fluorosolvatochromic methylated photoacid tris(2,2,2-trifluoroethyl) 8-methoxypyrene-1,3,6-trisulfonate (MePhos) and the free photoacid tris(2,2,2-trifluoroethyl) 8-hydroxypyrene-1,3,6-trisulfonate (Phos) were inserted into various MOF scaffolds, and the resulting emission properties were found to be far beyond the observed red shifts for polar solvents such as methanol or ethanol. Instead of the modulation of the band gap by the local environment given by the physicochemical properties of the MOF pores, there is aggregation of the MePhos molecules depending on the MOF structure, leading either to *H*- or t o *J*-like character.

## Introduction

Over the last years, research has focused on the replacement of inorganic conversion dyes by their organic counterparts with the aim of reducing or even completely substituting rare elements in LED materials. However, one major disadvantage of organic light emitters is their limited stability: oxygen, high temperatures, moisture, and continuous light exposure foster chemical and structural changes.^1−4^ Additionally, in the event of exceeding certain concentrations, crystallization and precipitation occur e.g., for Lumogen Red 305, which reduces or even completely extinguishes luminescence.^5^ For fluorescein molecules, this is known as self-quenching limit, which is due to a higher probability of energy transfer between neighboring molecules (i.e., aggregation-caused quenching, ACQ).^6^ Therefore, when integrated into high-power LEDs, certain precautions are required to prevent these processes.

A possible solution is to insert the organic dye into an encapsulating matrix, thereby generating stable and processable materials. Several studies have been performed in this area, ranging from polymer matrices,^7−9^ epoxy-based encapsulation materials,^10^ and silica matrices^11^ to polysiloxanes.^5^ For the latter, a tremendous improvement of the emission properties of two inserted perylene dyes was found as a result of the sterically demanding polymer matrix structure on the one hand, but mostly due to the direct covalent bonding of the dye molecules to this structure on the other.^5^ By this, crystallization and thus the formation of excimers was avoided, which otherwise would have quenched the luminescence efficiency. Nevertheless, there are remaining challenges such as the solubility and distribution of the organic dye as well as its stability within the encapsulation matrix. Both aspects can be influenced by a targeted modification of the host, as has been shown by the covalent integration of perylene dyes into polyphenylsiloxanes as a potential way to protect the molecules against harsh conditions (thermal impact and intense light exposure) present in LED applications.^5^

Within the context of metal–organic frameworks (MOFs), a class of organic–inorganic hybrid materials containing potential voids,^12^ only few examples are known in combination with fluorescent dyes: Zhou and co-workers designed a MOF, where they isolated perylene fluorophores to enhance fluorescence by avoiding ACQ, using linear organic linkers with perylene moieties as part of the backbone.^13^ A similar approach was presented by Wuttke and co-workers^14^ with prefunctionalized dye-linker molecules incorporated as a doping fraction into an MOF without losing any luminescence property. Forming LG@MOF systems (with LG referring to the luminescent guests) with the fluorescent moiety being noncovalently attached to the MOF framework has recently been reviewed by Gutiérrez and co-workers, showing the beneficial impact of such porous matrices on the emission behavior.^15^ The luminescence properties of, e.g., thioindigo were found to be significantly improved compared to the pristine solid dye upon MOF incorporation.^16^ In addition to the mere occurrence of luminescence, the deliberate incorporation of fluorophores and their corresponding structuring within the porous materials can also lead to the formation of *J*-aggregates. *J*-aggregates (*J* denotes Jelley), which are also called Scheibe aggregates, have first been described by Scheibe^17^ and Jelley^18^ independently. *J*-aggregates are characterized in particular by their very narrow and red-shifted absorption as well as emission bands, high fluorescence intensity, and small Stokes shifts compared to those of the monomer. Furthermore, delocalized excitons have been found in such aggregates with increased mobility.^19^ In combination with MOFs, various attempts have been made to realize the formation of *J*-aggregates and to take advantage of their optical characteristics: The fluorophore perylene-3,4,9,10-tetracarboxylate (PTC) has been found to show coordination-driven fluorescent *J*-aggregates as linker molecule in various MOFs.^20,21^ Focusing on coordination polymers containing the same ligand, emission bands pointing to the presence of *J*-aggregates were observed as well.^22^ Utilizing a diethynyl-anthracene link, *J*-aggregate density was directed within the interwoven PIZOF-2/NNU-28 structures.^23^ Wöll and co-workers realized a vast library of emissive MOF linkers and controlled their molecular alignment by utilizing steric control units (SCUs) for the formation of highly emissive *J*-aggregates.^24^ In a computational study, Wenzel and co-workers describe the crystalline assembly of perylene-based linkers showing an enhanced emission by the formation of perylene-*J*-aggregates.^25^ Recently, Janiak et al. have reported the formation of *J*-aggregates within MIL-53(Al) with enhanced fluorescence.^26^

**Figure 1:**
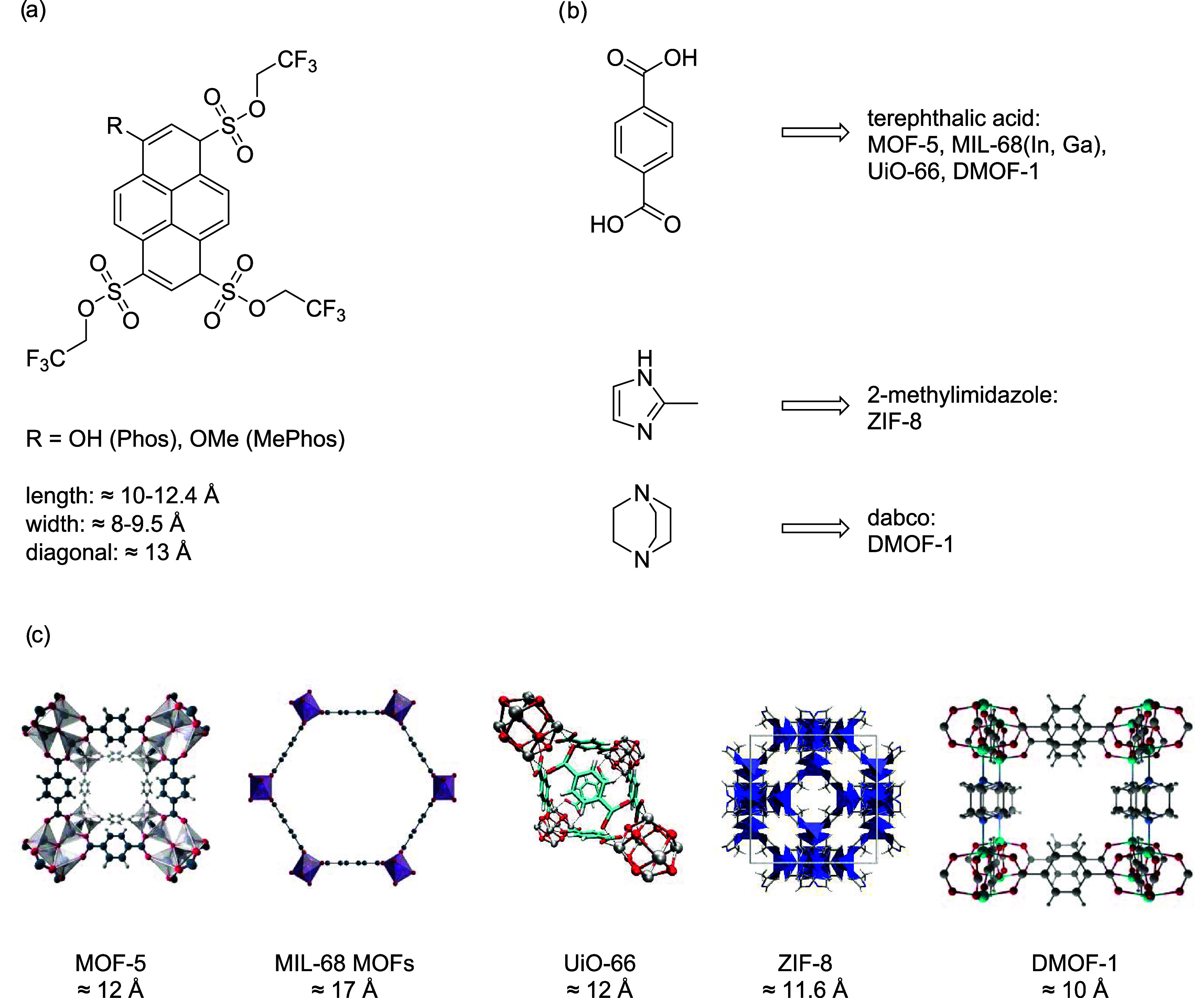
(a) Structures of Phos and MePhos with estimated molecular sizes; molecular sizes were estimated from ref (33). (b) Structures of the linker molecules with corresponding MOFs built up from these linker molecules (note: DMOF-1 is a mixed-linker MOF containing terephthalic acid and dabco). (c) MOF structures with estimated pore diameters in Å, crystal data were taken from refs (34−40). The structural motifs were visualized utilizing the program Diamond 4.4^41^ and the VMD program package.^42^

While most of these studies focused on perylene derivatives, organic photoacids have not been considered thus far and could find application in light-driven proton conduction.^27,28^ In the context of MOFs, light-driven proton conduction is also of interest, as MOFs offer a designable platform with high surface areas.^29^ Here, Stock and co-workers studied the influence of sulfonate groups and hydrogen bonding on the proton conduction of Mg-based coordination networks.^30^ Known for their ability to change pH upon light irradiation, pyranine-derived photoacids exhibit different emission maxima within various solvents depending on their polarity due to their fluorosolvatochromic^31^ behavior. Therefore, they have also been used to determine the polarity of various solvents.^32^ With the aim to realize both light-driven proton conduction within porous MOFs as carrier matrices as well as exploiting the solvatochromic response also in the solid state, the two pyrene compounds tris(2,2,2-trifluoroethyl) 8-methoxypyrene-1,3,6-trisulfonate (MePhos) and the tris(2,2,2-trifluoroethyl) 8-hydroxypyrene-1,3,6-trisulfonate (Phos) were chosen (see Figure 1a). While MePhos shows a methyl group at the R position, Phos has an OH group, which releases its proton upon irradiation.

Phos and MePhos were inserted into nine different MOFs exhibiting a large variety of metal nodes, linker molecules, and pore structures to account for both physicochemical but also spatial influence on the optical as well as acidic properties of the inserted guest molecules. Here, MOF-5,^34,35^ MIL-68(In),^36^ MIL-68(Ga),^36^ UiO-66,^37,38^ ZIF-8^39,43^ and DMOF-1^44^ were applied. Details on the structure and pore diameter are shown in Figure 1b,c. This variety of MOFs was chosen due to their intrinsic polarity, as has been recently reported for a phthalimide dye.^45^ Especially representatives of the MIL-68 family are of interest here, as they were found to beneficially contribute to the optical response of various photoactive dyes.^16,46−49^ Within the chosen porous hosts, both the light-driven change in pH and the optical behavior of Phos and MePhos are expected to be strongly related to the pore confinement in terms of size and structure. While for, e.g., MOF-5 “closed” pores are present with each pore being well-defined as a cube, MOFs of the MIL-family consist of channel-like structures. This structure can lead to cooperative effects between the embedded guest molecules themselves, resulting, for example, in the formation of *J*- or *H*-aggregates. Thus, the resulting properties of the overall material depend not only on the host–guest interactions but also on the guest–guest interactions.

## Experimental Section

### Chemicals

Commercially available terephthalic acid (Alfa Aesar), 1,4-diazabicyclo [2.2.2] octane (Sigma-Aldrich), chloroform (Carl Roth), gallium(III) nitrate hydrate (Thermo Fisher), indium(III) nitrate hydrate (Alfa Aesar), zinc(II) nitrate hydrate (Thermo Fischer), zirconium(IV)-chloride (Sigma-Aldrich), 2-methylimidazole (Sigma-Aldrich), *N*,*N*′-dimethylformamide (Fisher Scientific), triethylamine (Acros Organics), KBr (Sigma-Aldrich), zinc(II) acetate dihydrate (n.s.), pyrene (Sigma-Aldrich), Oleum (Sigma-Aldrich), acetic anhydride (VWR International), thionyl chloride (Fisher Scientific), 2,2,2-trifluoroethanol (Carbolution), and methyl iodide (Sigma-Aldrich) were used without further purification.

### Synthesis of MOF-5

MOF-5 was synthesized following the protocol given in the literature.^50^ 1.266 g of terephthalic acid (7.62 mmol) and triethylamine (2.13 mL) were dissolved in 100 mL of DMF. 4.25 g of Zn(OAc)_2_·2H_2_O (19.35 mmol) was dissolved in 125 mL of DMF. While stirring, the zinc salt solution was added dropwise to the organic solution over 15 min. The mixture was stirred for 2.5 h. The precipitate was filtered off and immersed in 62.5 mL of DMF overnight. It was then filtered off again and immersed in 87.5 mL of CHCl_3_. The solvent was exchanged 3 times over 7 days. Finally, the precipitate was decanted. To remove the solvent completely, the resulting powder was heated at 100 °C for 12 h under reduced pressure and stored under an argon atmosphere in a glovebox to prevent absorption of humidity and subsequent decomposition. The phase purity was confirmed by PXRD (see Figure S1).

### Synthesis of MIL-68(In)

**Figure 2:**
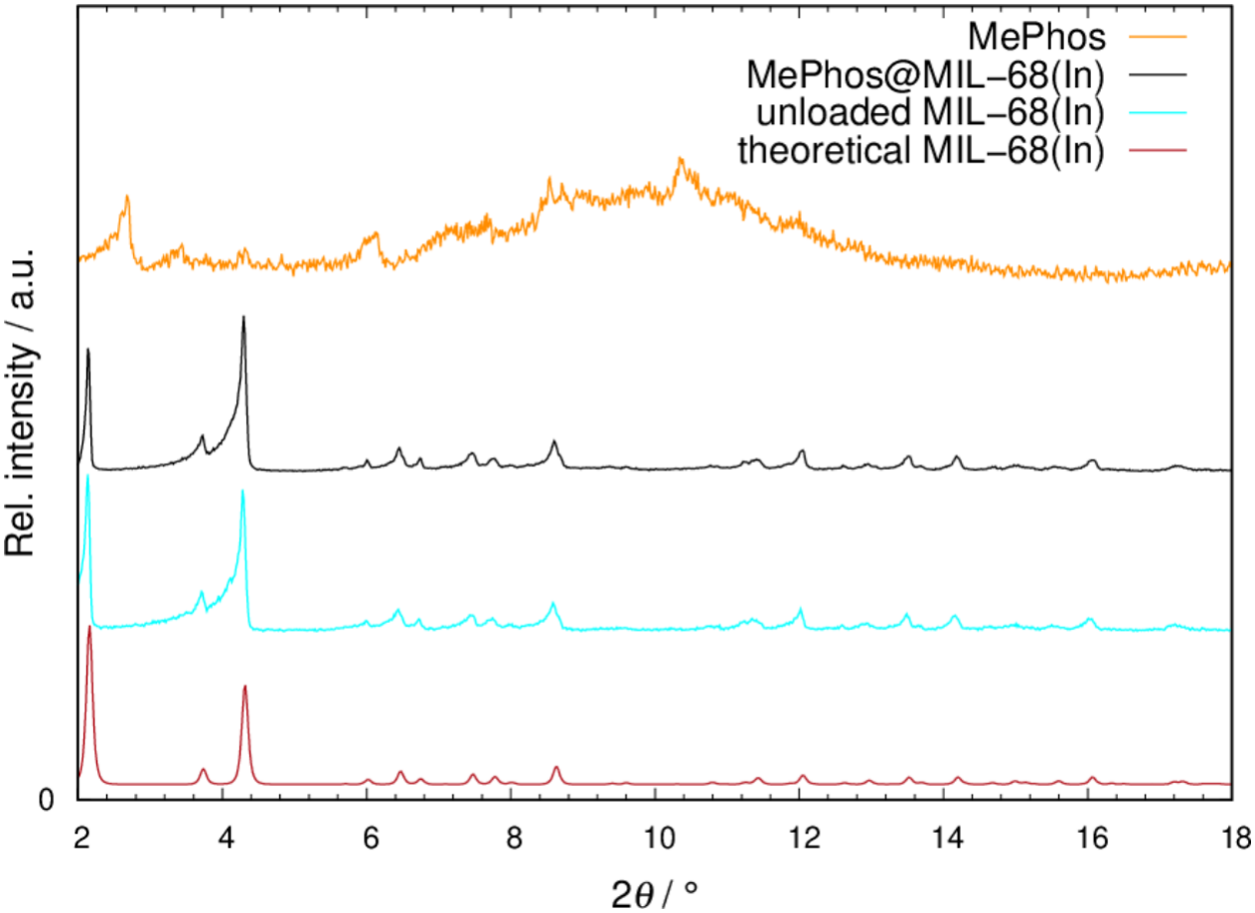
PXRD patterns of MePhos@MIL-68(In) (**8**) (black line) in comparison to pure MePhos (orange line), unloaded MOF (blue line), and the pattern generated from theoretical data (red line).

MIL-68(In) was synthesized following the protocol given in the literature.^36^ In(NO_3_)_3_·*x*H_2_O (408 mg, 1.04 mmol) and terephthalic acid (200 mg, 1.20 mmol) were added to 3.0 mL of DMF in a Teflon-lined autoclave. The vessel was heated at a rate of 20 °C h^–1^ to 100 °C, held for 48 h, and then cooled down to room temperature at a rate of 5 °C h^–1^. A white powder was recovered from the vessel and washed several times with DMF. Residual solvent was removed by first heating the sample to 200 °C in air for 12 h and then at 100 °C under reduced pressure for 1 h. The final product was stored under an argon atmosphere in a glovebox to prevent the absorption of moisture. The phase purity was confirmed by PXRD (see Figures 2 and S2).

### Synthesis of MIL-68(Ga)

MIL-68(Ga) was synthesized following the protocol given in the literature.^36^ Ga(NO_3_)_3_·*x*H_2_O (207 mg, 0.81 mmol) and terephthalic acid (100 mg, 0.60 mmol) were added to 3.0 mL of DMF in a Teflon-lined autoclave. The vessel was heated at a rate of 20 °C h^–1^ to 100 °C, held for 48 h, and then cooled down to room temperature at a rate of 5 °C h^–1^. A white powder was recovered from the vessel and washed with DMF several times. The residual solvent was removed by first heating the sample to 200 °C in air for 12 h and then at 100 °C under reduced pressure for 1 h. The final product was stored under an argon atmosphere in a glovebox to prevent absorption of humidity. The phase purity was confirmed by PXRD (see Figure S3).

### Synthesis of UiO-66

UiO-66 was synthesized following an adapted protocol given in the literature.^37^ ZrCl_4_ (0.212 g, 0.908 mmol) and terephthalic acid (0.136 g, 0.908 mmol) were dissolved in 120 mL of DMF at room temperature. The thus obtained mixture was sealed and placed in a pressure-stable glass vessel and heated to 120 °C for 24 h. Crystallization was carried out under static conditions. After slowly cooling down to room temperature (20 °C/h), the resulting solid was filtered, repeatedly washed with DMF, and stored afterward under an argon atmosphere in the glovebox to prevent absorption of humidity. The phase purity was confirmed by PXRD (see Figure S4).

### Synthesis of ZIF-8

ZIF-8 was synthesized following the protocol given in the literature.^51^ A solution of Zn(NO_3_)_2_·6H_2_O (1.174 g, 3.95 mmol) in 8 mL of DI water was added dropwise into a round flask to a solution of 2-methylimidazole (22.714 g, 276.66 mmol) in 80 mL of DI water. The mixture was stirred for about an hour. After that, the precipitate was decentrifuged. The liquid supernatant was decanted, and the solid residue was dried in an oven at 100 °C overnight. Afterward, the product was dried for 1 h at 100 °C and reduced pressure and stored under an argon atmosphere to prevent absorption of humidity. The phase purity was confirmed by PXRD (see Figure S5).

### Synthesis of DMOF-1

Zn(NO_3_)_2_·6 H_2_O (125.0 mg, 0.42 mmol), terephthalic acid (70.0 mg, 0.42 mmol), and dabco (1,4-diazabicyclo [2.2.2] octane) (20.0 mg, 21.0 mmol) were mixed with DMF (dimethylformamide) (3–5 mL) in an 8 mL Teflon-lined autoclave. The mixture was heated (120 °C, 2 days) in an oven and cooled down to room temperature afterward. The resulting colorless powder was filtered, then washed with a small amount of DMF, and dried on air overnight. To remove embedded DMF molecules, the residue was heated under reduced pressure (120 °C, 24 h) and stored under argon atmosphere. The phase purity was confirmed by PXRD (see Figure S6).

### Synthesis of Tris(2,2,2-trifluoroethyl)-8-hydroxypyrene-1,3,6-trisulfonate (Phos)

The unprotected photoacid was synthesized following the protocol given in the literature.^52^

### Synthesis of Tris(2,2,2-trifluoroethyl)-8-methoxypyrene-1,3,6-trisulfonate (MePhos)

Tris(2,2,2-trifluoroethyl) 8-hydroxypyrene-1,3,6-trisulfonate (90 mg, 0.13 mmol) was dissolved in 10 mL of acetone. After addition of methyl iodide (15.9 μL, 0.26 mmol) and potassium carbonate (44.7 mg, 0.26 mmol), the mixture was stirred for 48 h at room temperature. Solvent and excess methyl iodide were removed in vacuo. The residue was solved in ethyl acetate and washed with water (2 × 50 mL) and saturated sodium chloride solution (2 × 50 mL). The organic phase was separated and dried over sodium sulfate. The crude product was purified by column chromatography (PE/EE = 6:4).

### Synthesis of Phos@MOF and MePhos@MOF Systems

Phos and MePhos molecules were incorporated into the host lattices at low concentrations via a gas phase loading process at elevated temperatures under reduced pressure. In Tables S1 and S2 (for Phos) and Tables S3 and S4 (for MePhos), the weighed-in masses, quantities in mol, and insertion temperatures are listed. For all syntheses, a molar guest:host ratio of 0.125:1 was applied. The phase purity was confirmed by PXRD (see Figures S1–S11).

### Powder X-ray Diffraction (PXRD)

PXRD measurements were performed on a Stoe Stadi P diffractometer (Stoe, Darmstadt, Germany) in transmission geometry with Mo-Kα_1_-radiation (λ = 70.93 pm) utilizing a focusing Ge(111) primary beam monochromator and a Mythen 2 DCS4 detector. Data was collected in the 2θ range of 2.0–40.4° with a step size of 0.015°. Data were measured at 298 K (Stoe Stadi P: λ = 0.7093 Å). All PXRD patterns can be found in Figures 2 and S1–S11.

### Liquid-State Nuclear Magnetic Resonance (NMR) Spectroscopy

^1^H NMR spectra were collected on a 300 MHz Bruker Avance DPX NMR spectrometer equipped with a 5 mm broadband probe. The solvent served as an internal reference (δ_H_(DMSO-*d*_6_) = 2.50 ppm). Measurements were carried out at room temperature and processed with MestReNova 9.0.1-13254. For each measurement, the samples were dissolved in 0.5 mL of DMSO and 25 μL of DCl for decomposing the host framework.^53^^1^H NMR spectra of both the individual components and the (Me)Phos@MOF systems can be found in Figures S12–S28, and details on the compositions determination are listed in Table S5.

### Photoelectron Spectroscopy (XPS)

XPS measurements on Phos@UiO-66 and MePhos@UiO-66 were performed as follows: The respective powder was placed on an adhesive copper foil. Measurements were performed on a multichamber UHV system at a pressure of 5 × 10^–10^ mbar using a Phoibos 100 hemispherical analyzer (Specs). A Mg Kα anode was used as excitation source (*h* × *ν* = 1252.6 eV, probing depth ∼10 nm). As a result of charging effects during measurements, the binding energy scale was shifted by a few eV. Therefore, the binding energies were corrected such that adventitious carbon is positioned at 284.8 eV. Integrated peak areas of characteristic core level excitations were used to calculate the embedded amounts of Phos and MePhos inside UiO-66. The peak areas of F and Zr were evaluated and corrected by their relative sensitivity factors (RSF).^54^ XPS spectra with fits and details on the calculation process can be found in Figures S29 and S30 as well as in Table S6.

### Infrared (IR) Spectroscopy

IR spectroscopic measurements were carried out on a Bruker Alpha II FT-IR-Spectrometer under an argon atmosphere to exclusively elucidate host–guest and guest–guest interactions. Samples were prepared by diluting the thoroughly ground analyte powder by approximately one-third using KBr. The resulting homogeneous mixture was pressed to a thin transparent KBr pellet with a set pressure of ∼2 tons for 30 min. Scans were done in the range of 360–4000 cm^–1^ with a resolution of 2 cm^–1^ and 90 scans per sample. The background (a pure KBr pellet) was measured with the same instrument settings as those of the sample. All measurements were carried out at RT and evaluated with the program OPUS version 8.2 build 8, 2, 28(20190310) Copyright Bruker Optic GmbH. For the illumination of the hybrid materials, a Prizmatix PRI FC5-LED-WL (five high-power Fiberglas coupled LEDs output with a potentiometer for manual power control) was used. IR spectra can be found in Figures S31–S42.

### UV/Vis Diffuse Reflectance Spectroscopy (DRS)

Reflectance spectra of the pristine MOFs as well as the (Me)Phos@MOF systems were recorded by using an Agilent Cary 5000 UV–vis–NIR spectrophotometer except for UiO-66, where a PerkinElmer Lambda 750 spectrometer was used. Spectra were recorded in the range of 200–800 nm before and after irradiation. For the illumination of the hybrid materials, a Prizmatix PRI FC5-LED-WL (five high-power Fiberglas coupled LEDs output with potentiometer for manual power control) was used. The DRS spectra can be found in Figures S43–S54.

### Steady-State Spectroscopy

**Figure 3:**
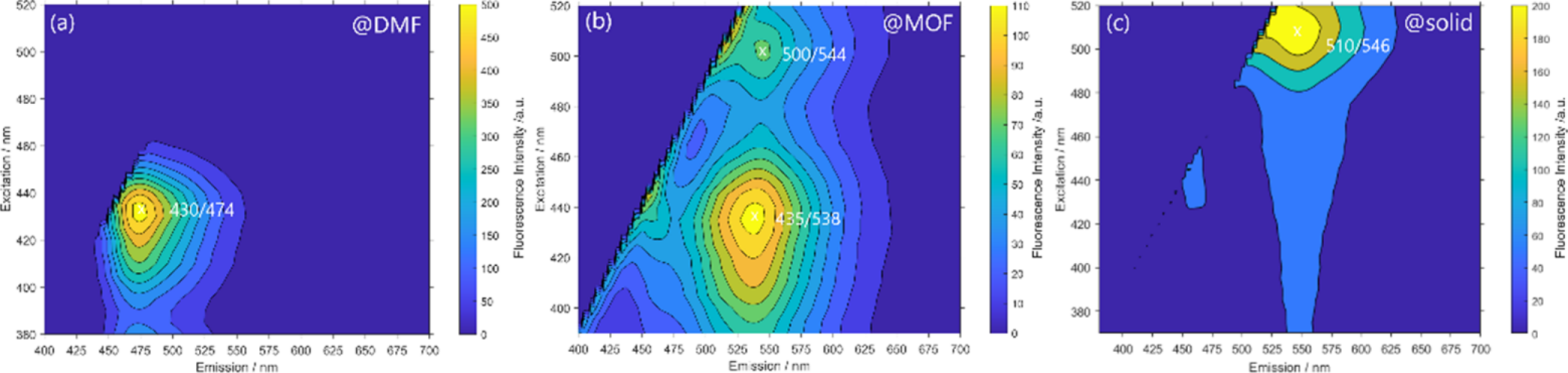
2D contour plot of emission-excitation fluorescence intensity. (a) MePhos in dimethylformamide, (b) MePhos@MIL-68(In) (**8**), and (c) solid-state spectra of MePhos.

**Figure 4:**
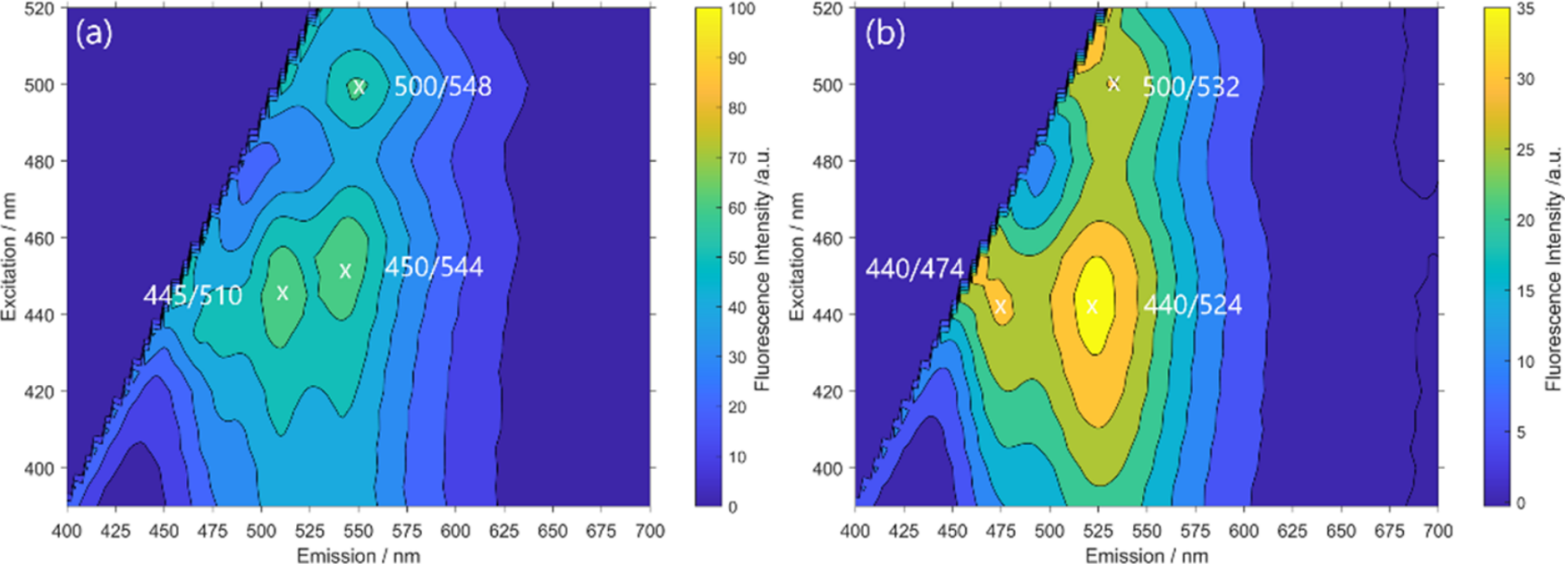
2D contour plot of emission-excitation fluorescence intensity of MePhos@DMOF-1 (**12**) (a) and MePhos@UiO-66 (**10**) (b).

The 2D fluorescence emission spectra were recorded with a Jasco Spectrofluorometer FP-6500 between λ_exc_ = 390–520 nm and λ_em_ = 400–700 nm. The quantum yields were measured by means of a commercial instrument (Quantaurus-QY C11347, Hamamatsu). All samples were transferred to capillary tubes (Superior Marienfeld, ⌀ = 1.15 mm) in both experiments. The 2D contour plots of emission-excitation fluorescence intensity can be found in Figures 3, 4, and S55–S57.

### Time-Correlated Single-Photon Counting (TCSPC)

**Figure 5:**
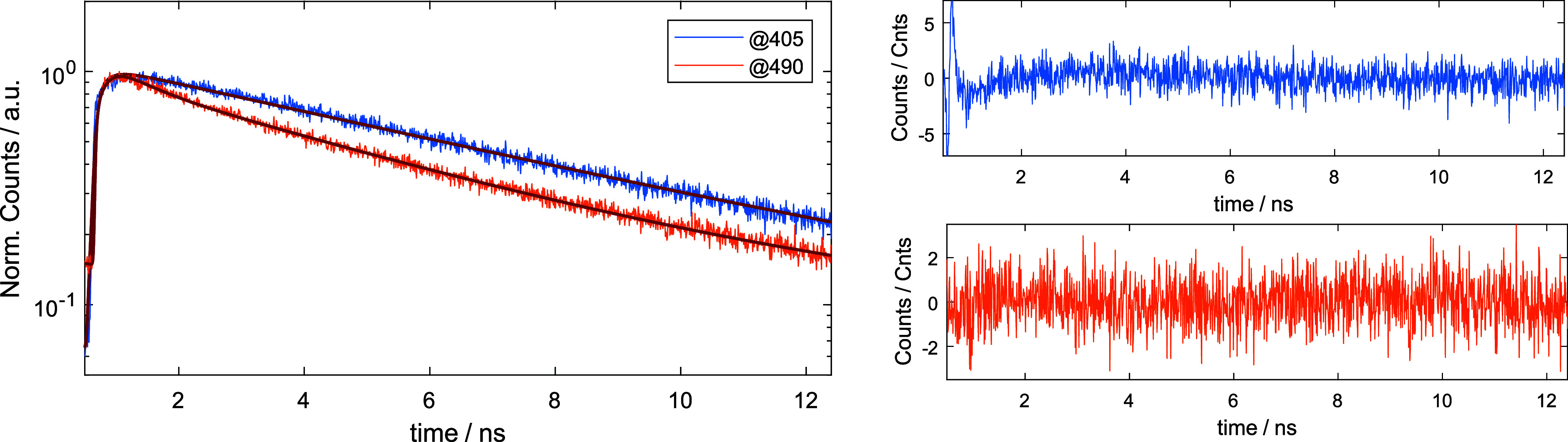
TCSPC histogram of MePhos@MIL-68(Ga) (**9**) sample at different excitation wavelengths (λ_exc_ = 405 nm/λ_det_ = 500–550 nm and λ_exc_ = 490 nm/λ_det_ = 525–645 nm) with the fit residuals for both traces (right).

The lifetime measurements were performed under magic angle conditions. The data were recorded by the following setup: A pulsed laser diode operating at λ_exc_ = 405 nm (LDH-PC-405, PicoQuant, 40 MHz, pulse duration <50 ps) or another pulsed laser (λ_exc_ = 490 nm, FemtoFiber pro TVIS, Toptica Photonics, 80 MHz, pulse duration < ps) were used as excitation source. For detection, a photocounting detector (PDM series, Micro Photon Devices) and a photocounting device (Pico Harp 300, PicoQuant) were used. The data were analyzed with commercial software (SymPhoTime, PicoQuant). To record the instrumental response function (IRF), a diluted colloidal silica solution (LUDOX TM-50, Sigma-Aldrich) was utilized, resulting in a fwhm of approximately 300 ps for the λ_exc_ = 405 nm laser diode and 65 ps for λ_exc_ = 490 nm. Lifetimes τ_fl_ below 0.1 ns are not considered in any further detail here, as these could be the result of scattering effects or a mismatched IRF. The TCSP histograms can be found in Figures 5 and S58–S65.

## Results and Discussion

Loading of Phos and MePhos was performed via a gas phase process to exclude any influence of residual solvent molecules on the optical characteristics of the compound materials.^47,48^ In total, 12 new hybrid systems were synthesized, which are listed in Table 1.

**Table 1:** List of Synthesized Phos@MOF and MePhos@MOF Systems Obtained via Gas Phase Loading at Elevated Temperatures

Phos@MOF systems	MePhos@MOF systems
Phos@MOF-5 (**1**)	MePhos@MOF-5 (**7**)
Phos@MIL-68(In) (**2**)	MePhos@MIL-68(In) (**8**)
Phos@MIL-68(Ga) (**3**)	MePhos@MIL-68(Ga) (**9**)
Phos@UiO-66 (**4**)	MePhos@UiO-66 (**10**)
Phos@ZIF-8 (**5**)	MePhos@ZIF-8 (**11**)
Phos@DMOF-1 (**6**)	MePhos@DMOF-1 (**12**)

Due to the molecular size of Phos and MePhos and the pore diameters of the chosen MOFs (see values given in Figure 1), a successful incorporation as noncovalently attached guest molecule of both dyes is potentially possible. We followed the successful incorporation by PXRD, where intensity modulations and the absence of dye-related reflections confirm the dye insertion (Figures 2 and S1–S11). Upon incorporation into the rigid framework, the electron density of the overall material changes, which leads to changes in the structure factor and therewith to modulations in reflection intensities for rigid MOFs. This has already been reported e.g., for azobenzenes,^48,49^ spiropyrans^47,,56^ or thioindigo dyes.^16^ Additional reflections, however, point to noninserted free crystalline dye. Therefore, we also recorded the PXRD patterns of Phos and MePhos to exclude this. In Figure 2, the diffraction patterns of MePhos@MIL-68(In) (**8**), the pure dye MePhos, the unloaded MIL-68(In), and the theoretical pattern of MIL-68(In) are compared. Here, the intensity modulations of the reflections become especially apparent for the first peak (at approximately 2° 2θ), where the intensity decreases when compared to the nonloaded MOF. For MOF-5, MIL-68(Ga), and ZIF-8 as MOF hosts, shifts to higher 2θ values are present indicating the presence of attractive host–guest interactions, which has been previously reported for other guest@MOF systems.^47,57^ Notably, a significant broadening of the reflections of UiO-66 occurs upon inclusion of Phos and MePhos inclusion. This broadening may be caused by a decrease in the crystallinity as a result of dye embedment.

The composition of the hybrid systems was determined by liquid-state NMR spectroscopy following the procedure reported by Benedict and co-workers^53^ and, in the case of nondigestible UiO-66, by XPS. Using the first method, the characteristic proton signals of the guest as well as the MOF linkers were related to each other (see Figures S19–S28 and Table S5). Details on the calculation process via liquid-state NMR can be found in the Supporting Information. For each sample, three different sample fractions were taken. Notably, we observed inhomogeneous loading for each sample, which has also been found for a DTE inside a MOF.^53^ For XPS, the characteristic core level values of the Zr metal-cation and the guest specific fluorine atom were analyzed and related to each other under consideration of the elemental and orbital specific relative sensitivity factors (RSF),^54^ details can be found in the Supporting Information (Figures S29 and S30 and Table S6). In Table 2, the obtained compositions of the Phos@MOF and MePhos@MOF systems are listed. Notably, there are small deviations in comparison to the weighed-in guest-to-host ratios for both the NMR and the XPS measurements. For the first method, these can be explained by the very small amounts being embedded and the signal-to-noise ratio, as well as inhomogeneous loading. For the latter, shielding of the metal nodes due to a positioning of the dyes near the highly charged SBU of UiO-66^16^ might also lead to higher values than weighed in. When working with such small sample quantities, variations in the determination of the composition may occur, and the values obtained should be understood as indicative.

**Table 2:** Compositions of the Phos@MOF and MePhos@MOF Systems Obtained by Liquid-State NMR and XPS* Measurements

	composition *n*(Phos)/*n*(MOF)	composition *n*(MePhos)/*n*(MOF)
MOF-5	0.09:1 (**1**)	0.09:1 (**7**)
MIL-68(In)	0.11:1 (**2**)	0.21:1 (**8**)
MIL-68(Ga)	0.10:1 (**3**)	0.12:1 (**9**)
UiO-66*	0.24:1 (**4**)	0.16:1 (**10**)
ZIF-8	0.07:1 (**5**)	0.08:1 (**11**)
DMOF-1	0.10:1 (**6**)	0.13:1 (**12**)

The interaction of both fluorophores with the respective MOF host can be traced via IR spectroscopic measurements. Changes in the band intensities and shifts play a particularly important role here. For a precise analysis of these interactions, we measured both the signatures of the individual components and the vibrational patterns of the entire system. Within the MOF host, the IR bands of MePhos and of the respective MOF host are still present and do not change their position, as is visible e.g., for the most prominent band at ∼1225 cm^–1^ (ν_as_ = C–O−). This is true for all studied systems (see Figures S31–S42), indicating an environment given within the MOF pores that is comparable to a solvated or gaseous state. The vibrational characteristics of the Phos and MePhos molecules do not experience any change upon incorporation into the MOF and therefore, these guests seem to be not localized at a specific position.

In the next step, UV/vis diffuse reflectance spectroscopy was utilized to determine the maximum interaction wavelength of both pure Phos and MePhos and the nonloaded MOF host on the one hand, and when embedded inside the different MOF hosts on the other. The respective spectra can be found in the Supporting Information (Figures S43–S54), the positions of the reflection minima are summarized in Table 3. Notably, none of the applied MOF hosts shows a reflection minimum in the visible region, and thus, the optical characteristics of the host itself do not interfere with the guest molecules.

**Table 3:** Positions of the Reflection Minima of the Phos@MOF and MePhos@MOF Systems

	reflection minima (nm) for Phos	reflection minima (nm) for MePhos
MOF-5	431/541 (**1**)	431 (**7**)
MIL-68(In)	434/547 (**2**)	430 (**8**)
MIL-68(Ga)	429/549 (**3**)	434 (**9**)
UiO-66	454/547 (**4**)	430 (**10**)
ZIF-8	560 (**5**)	430 (**11**)
DMOF-1	436/534 (**6**)	430 (**12**)

Within all studied MOFs, Phos and MePhos show broad reflection minima and host-dependent shifts in the reflection minimum position in comparison to the pristine dyes. These shifts are present but not visible to the naked eye. Based on this, we hypothesized that energetic levels of chromophores within MOFs could be heavily modified as a result of their interaction with the MOF scaffold. Previously, we characterized the chemical interactions in silica nanoparticles with pyrene derivatives in a similar way;^58^ these probe molecules should also serve to characterize the interactions in this study due to the very clear dependence of their transition energies on different solvent properties.^32^ It should be mentioned that the Phos and the Phos@MOF samples show no significant measurable fluorescence (see Figures S55 and S56), and the quantum yields are also below 1%. The pronounced red shift of the electronic spectra (Table 3) compared to MePhos suggests fluorescence quenching in all Phos@MOF samples due to the energy gap law.^29^ In contrast, the MePhos@MOF solids show yellowish-green fluorescence in all cases with quantum yields between 3.5 and 16%, which is comparable to the quantum yields observed in the pure MePhos solid with approximately 5%. It would appear that in some MOFs fluorescence is enhanced, whereas in others, it is reduced. Therefore, only the MePhos@MOF samples are considered in the following discussion. Furthermore, MePhos@MOF-5 is not further detailed due to stability issues during the measurements. The pure MOFs were also analyzed using fluorescence spectroscopy (see Figure S57). Only ZIF-8 shows an emission maximum around λ_exc_/λ_em_ = 425/458 nm, but this is not seen in the MePhos@ZIF-8 (Figure S55b). Also, the quantum yield of ZIF-8 is less than 1%, and hence is insignificant in this context.

Following the idea that energetic levels of chromophores within MOFs could be heavily modified as a result of their interaction with the MOF scaffold, the optical properties of our probe molecules in different MOF structures were characterized by steady-state and time-resolved fluorescence spectroscopy. We used 2D spectra, as these are superior compared to ordinary excitation and emission spectra as heterogeneities can be resolved, but the information on one-dimensional spectra can be easily recovered. Spectra of the pure dye in polar solvent and in the solid state were always measured for comparison. In agreement with previous findings,^32^ our probe molecule MePhos in dimethylformamide shows one band *at* λ_exc_/λ_em_ = 430/474 nm (Figure 3a). In contrast, the solid-state spectrum of the bulk material shows a red-shifted band at λ_exc_/λ_em_ = 510/546 nm (Figure 3c). The pronounced bathochromic shifts are, presumably, due to aggregation of the methoxy derivate and point, therefore, to a *J*-aggregate-like behavior.

The two-dimensional excitation–emission spectra of prototypical MePhos@MIL-68(In) (**8**) are depicted in Figure 3b. At first glance, the global maximum (λ_exc_/λ_em_ = 435/538 nm) of the 2D map roughly corresponds to the excitation maximum of the free dye and the emission maximum of the aggregate. A more detailed inspection of the 2D map, however, shows, first, that the emission originates from two distinct excitation wavelengths and, second, that the position of the emission maxima slightly differs. The most red-shifted excitation wavelength maximum is found at λ_exc_ = 500 nm and λ_em_ = 544 nm. These data are close to those of the pure solid dye and differ by only a few nanometers. We therefore conclude that aggregation takes place in MOFs, although with a slightly modified packing directed by the specific MOF pore structure. For MOFs of the MIL-series, this *J*-aggregation behavior is found and assumed to be the result of the channel-like pore structure.

The short excitation wavelength maximum is located at λ_exc_ = 435 nm, slightly red-shifted to the reflection minima (see Table 3). We assume based on these findings that isolated MePhos molecules can be found within the MOF scaffold, as the loading fraction of the structures is significantly below 1 (see Table 2). In this case, the excitation leads to an emission at λ_em_ = 538 nm, only blue-shifted *y* < 10 nm compared to the aggregate. The associated emission is bathochromically shifted by 64 nm in comparison to the maximum observed for the methylated dye in dimethylformamide (λ_em_ = 474 nm), which agrees with the conclusion that an energy transfer from the isolated molecules to aggregates is possibly occurring.

Similar results were obtained for the other systems (see Figure S55) except for DMOF-1 (**12**) and UiO-66 (**10**) host. Here, in the 2D spectra (Figure 4), an additional weaker maximum at λ_exc_ = 445 nm and λ_exc_ = 440 nm is detected, respectively. The corresponding emission is hypsochromically shifted relative to the presumed energy transfer band by tens of nanometers (34 nm (**12**), 50 nm (**10**)). Although the emission maxima still suggest some aggregation, the exact geometry is hardly predictable only based on spectroscopic steady-state data.

Similarly, no correlation between the intensity ratios between the two overall maxima and the loading was noticed.

Furthermore, the photostability of the samples was also evaluated. The absolute values of the fluorescence intensity are difficult to determine. Although the absolute intensity values may be interpreted with care, photobleaching was noticed for all samples (see Figures S66–S68). The data therefore do not support any photostability increase in the MOF structures, but the quantification of this phenomenon is beyond the scope of this study.

To study the MePhos@MOF samples and the distinct maxima in more detail, we measured the fluorescence lifetime by time-correlated single-photon counting (TCSPC).

Figure 5 shows a prototypical TCSPC emission decay of MePhos@MIL-68(Ga) (**9**) at two different excitation wavelengths with the aim to excite specifically the two different detected bands. Fluorescence filters enable further selection (Figures S58–S65). None of the observed fluorescence decays follow a monoexponential pattern. This indicates that more than one process influences the lifetime of the MePhos@MOF samples and might give a hint to heterogeneity in the samples, which was noticed already in the 2D spectra (see Table S7).

The fluorescence lifetime τ_*fl*_ measured with λ_exc_ = 490 nm was detected at λ_det_ = 525–645 nm and yields as longest component a lifetime that ranges between 4.34 and 6.57 ns. This value is not far from the lifetime of the pure solid dye (τ_fl_ = 5.79 ns), and we assume that this represents an optically allowed transition. It can be further assumed that the variation of the lifetime τ_fl_ is due to the different environments in each individual MOF structure. However, considerable quenching must take place as distinctly shorter lifetimes are found here as well with large amplitudes. Therefore, the average lifetime τ_fl_ is always below 2 ns.

However, when excited at λ_exc_ = 405 nm, two cases must be distinguished. By detection at λ_det_ = 500–550 nm or for the MePhos@DMOF-1 (**12**) at λ_det_ = 565–615 nm, that is covering the emission of the putative aggregation band, longer lifetimes are measured than with λ_exc_ = 490 nm (see Figures S62–S65). This is true for both the average lifetime and the longest lifetime component. A more extended range of τ_fl_ between 6.67 and 10.8 ns is observed for the longest lifetime component which is considerably larger than that of the free dye in the solid state. We therefore conclude that here the aggregation favors a geometry where the transition is less allowed. In terms of aggregation phenomena, the character of this transition is more *H*-like compared with the more red-shifted transition. However, given a more *H*-like aggregation structure, we cannot exclude that the shorter wavelength excitation maximum (which we assigned based on steady-state spectra to an energy transfer band) corresponds to the upper excitonic state which is only completely forbidden in a perfect *J*-aggregate. For MePhos@DMOF-1 (**12**) and MePhos@UiO-66 (**10**), which show a third band in the two-dimensional (2D) spectrum, TCSPC data were determined at λ_det_ = 450–490 nm (**12**) or λ_det_ = 417–477 nm (**10**), selecting the short emission band. The respective measured longest lifetimes τ_*fl*_ are 4.33 and 4.73 ns, but the average lifetime indicates strong quenching.

To summarize, the measured longest lifetimes τ_fl_ at λ_exc_ = 490 nm, which presumably indicate aggregation, are shorter than those observed in the putative energy transfer band after λ_exc_ = 405 nm, as for the latter, the longest lifetime component is larger than that for the free dye. We assign a more pronounced *H*-character to this transition, while the long wavelength transitions are more *J*-like. This explanation would also rationalize the larger red shift as a result of a larger Davydov splitting.^59−61^ The aggregation characteristics found are related to the MOF pore structure, which is channel-like (MIL-68 MOF derivatives) with a large pore diameter in the case of the more *J*-aggregate behavior, while *H*-aggregate behavior is found for MOF with small pore diameters and closed pores (ZIF-8, DMOF-1, and UiO-66). The reason for this is assumed to be of a sterical nature, as the *J*-aggregates require more space than the *H*-aggregates, which in turn explains why infinite channels within the MIL-68 MOFs enable this type of aggregation to occur. In Table 4, the average fluorescence lifetimes and quantum yields of the studied MePhos@MOF samples are listed.

**Table 4:** Average Fluorescence Lifetimes and Quantum Yields of the MePhos@MOF Samples

MePhos@MOF samples	τ_fl_, λ_exc_ = 405 nm (ns)	τ_fl_, λ_exc_ = 490 nm (ns)	excitation/emission maxima (nm) for MePhos	QY (%)
MePhos@MIL-68(In) (**8**)	3.29^a^	1.55^e^	435/538, 500/544	13
MePhos@ZIF-8 (**11**)	2.58^a^	0.94^e^	455/542, 500/548	3.5
MePhos@DMOF-1 (**12**)	1.96^d^	1.69^e^	450/544, 500/548	6.6
	0.43^b^		445/510	
MePhos@MIL-68 (Ga) (**9**)	6.80^a^	1.96^e^	440/510, 490/512	16
MePhos@UiO-66 (**10**)	0.79^a^	0.70^e^	445/524, 500/532	9.5
	0.30^c^		445/574	

a λ_det_, nm: 525/50.

b λ_det_, nm: 470/40.

c λ_det_, nm: 447/60.

d λ_det_, nm: 590/50.

e λ_det_, nm: 585/120.

## Conclusions

Within this work, we have demonstrated the successful combination of two pyrene compounds tris(2,2,2-trifluoroethyl) 8-methoxypyrene-1,3,6-trisulfonate (MePhos) and tris(2,2,2-trifluoroethyl) 8-hydroxypyrene-1,3,6-trisulfonate (Phos) with several MOF host frameworks to form the first hybrid pyrene-derived compound@MOF systems. On the one hand, the Phos@MOF systems were expected to exhibit light-driven proton release with Phos being the photoactive moiety. However, due to the aggregation effects of Phos within each host matrix applied, this could not be realized. For MePhos on the other hand, the solvatochromic response was expected as a function of the local environment. Although solvatochromic effects are typically observed in liquids, similar effects might arise due to the spatial freedom and the local chemical environment within the MOF. This environment could influence the electron density of the MePhos molecule, causing a red shift in the emission. For MePhos within the various MOFs, we found a prominent red shift, which is not comparable to any emission characteristics found in any solvent so far. Utilizing steady-state fluorescence spectroscopy and lifetime measurements, several aggregation geometries were found for the incorporated fluorophore, which are distinguishable by their fluorescent properties: On the one hand, more *H*-like (but still predominantly of *J*-type) aggregates are traceable due to π–π stacking, which is visible in a slightly less splitting of the emission maxima and longer fluorescence lifetimes, and on the other, *J*-like aggregates are present, which are visible in a prominent red shift and shorter fluorescence lifetimes.

The emission characteristics do, thus, depend not only on the given MOF pore environment, but the confined spaces can also induce cooperative effects between the guest molecules themselves to form either *J*- or *H*-aggregates. For applications, e.g., in LED technology or light-driven proton conduction, smaller MOF geometries, where photostable fluorophores with detrimental, strong aggregation tendencies are forced to align in *J*-aggregates, could be an alternative to other encapsulation methods and might pave the way for MOF in optical technologies.

## Supporting Information Available

PXRD patterns of Phos@MOF systems and MePhos@MOF systems in comparison to measured and simulated patterns of the unloaded MOF host and the pristine guest molecule; ^1^H NMR spectra of Phos and MePhos of the pure MOFs and the (Me)Phos@MOF systems; XPS spectra of the Phos@UiO-66 and MePhos@UiO-66 with fits of the characteristic core level and N 1s peak; IR spectra of pure (Me)Phos compared to the respective MOF host as well as the corresponding (Me)Phos@MOF systems; diffuse reflectance spectra of pure (Me)Phos compared to the respective MOF host as well as the corresponding (Me)Phos@MOF systems; 2D contour plot of emission-excitation fluorescence intensity of Phos@MOF and MePhos@MOF systems; TCSPC histograms of MePhos@MOF systems; relevant spectra for the determination of photostability; weighed-in masses, quantities in mol, and temperatures for the gas phase synthesis of the (Me)Phos@MOF systems; relevant values for the determination of composition of (Me)Phos@MOF by NMR; relevant values for the determination of composition of (Me)Phos@UiO-66 materials by XPS; and fluorescence lifetimes of the MePhos@MOF systems. (PDF)
