# Supplementary material for: Confinement-Driven Aggregate Formation of Photoacids within Porous Metal–Organic Frameworks
Source: ACS Omega. 2025 Jan 30;10(5):4711–21. doi: 10.1021/acsomega.4c09621 (PMC11822499; doi:10.1021/acsomega.4c09621)
Supplement: Supplementary file 1 — ao4c09621_si_001.pdf [file ao4c09621_si_001.pdf]

Supporting Information

Confinement driven aggregate formation of photoacids within porous metal-organic frameworks

Markus Rödl[a,§], Viktoria Kiefer[b, §], Selina Olthof[c], Klaus Meerholz[c], Gregor Jung[b], and Heidi A. Schwartz[a,*]

[a] *Institute of General, Inorganic and Theoretical Chemistry, Universität Innsbruck, Innrain 80-82, A-6020 Innsbruck,*

[b] *Biophysical Chemistry, Saarland University, Campus, Building B2 2, D-66123 Saarbrücken, Germany,*

[c] *Department of Chemistry, University of Cologne, Greinstraße 4-6, D-50939 Cologne, Germany,*

Corresponding author:

Heidi A. Schwartz

Email: heidi.schwartz@uibk.ac.at

[§] *M. R. and V. K. contributed equally to this work*

Content

Figure S1 to S11. PXRD patterns of Phos@MOF systems (**1-6**) and MePhos@MOF systems (**7**, **9-12**) in comparison to measured and simulated patterns of the unloaded MOF host und the pristine guest molecule.

Figures S12 to S28. ^{1}H NMR spectra of Phos and MePhos, of the pure MOFs and the (Me)Phos@MOF systems.

Figures S29 and S30. XPS spectra of the Phos@UiO-66 and MePhos@UiO-66 with fits of the characteristic core level and N 1s peak.

Figures S31 to S42. IR spectra of pure (Me)Phos compared to the respective MOF host as well as the corresponding (Me)Phos@MOF systems.

Figures S43 to S54. Diffuse reflectance spectra of pure (Me)Phos compared to the respective MOF host as well as the corresponding (Me)Phos@MOF systems.

Figures S55 to S57. 2D-contour plot of emission-excitation fluorescence intensity of Phos@MOF and MePhos@MOF systems.

Figure S58 to S65. TCSPC histograms of MePhos@MOF systems.

Figure S66 to S68. Relevant spectra for the determination of photostability.

Tables S1 to S4. Weighed-in masses, quantities in mol, and temperatures for the gas phase synthesis of the (Me)Phos@MOF systems.

Table S5. Relevant values for the determination of composition of (Me)Phos@MOF by NMR.

Table S6. Relevant values for the determination of composition of (Me)Phos@UiO-66 materials by XPS.

Table S7. Fluorescence lifetimes of the MePhos@MOF systems.

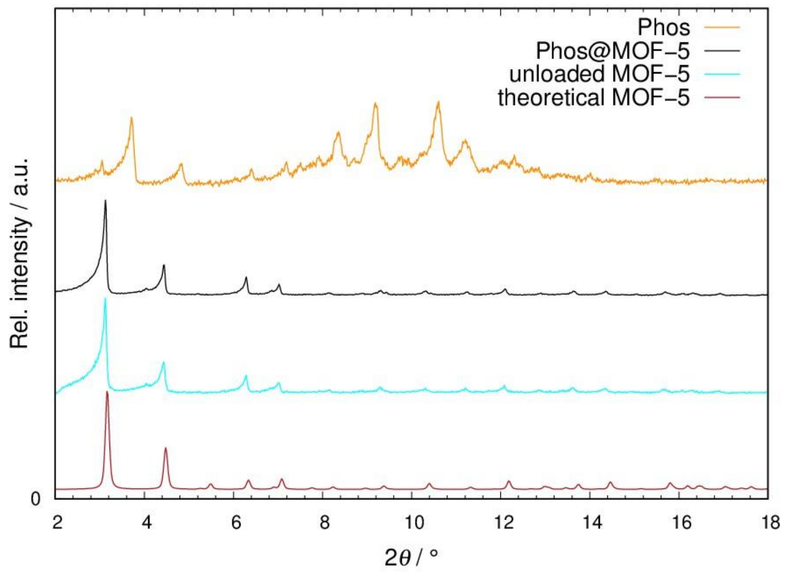

Figure S1. PXRD patterns of Phos@MOF-5 (**1**) (black line) in comparison to pure Phos (orange line), the unloaded MOF (blue line) and the pattern generated from theoretical data (red line).

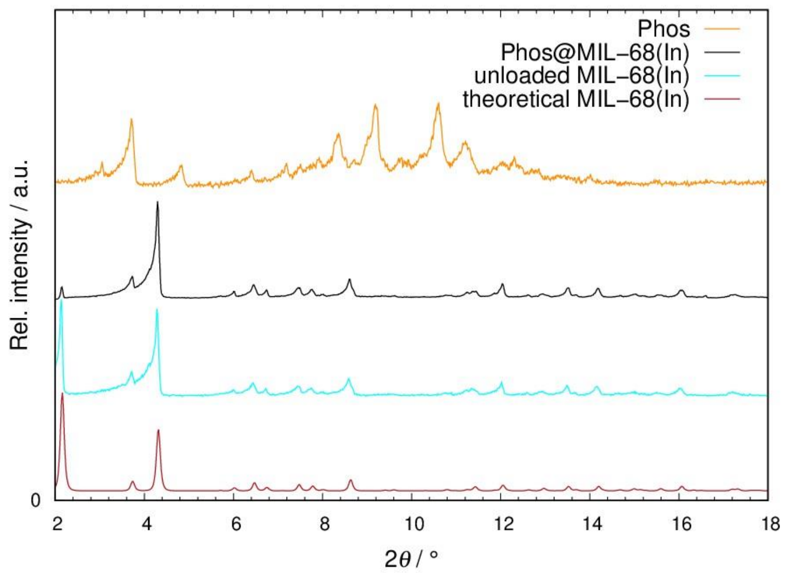

Figure S2. PXRD patterns of Phos@MIL-68(In) (**2**) (black line) in comparison to pure Phos (orange line), the unloaded MOF (blue line) and the pattern generated from theoretical data (red line).

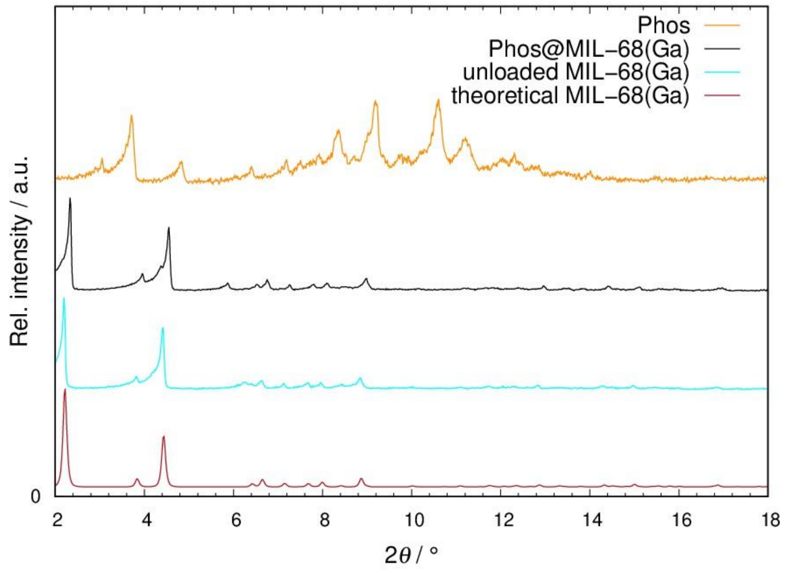

Figure S3. PXRD patterns of Phos@MIL-68(Ga) (**6**) (black line) in comparison to pure Phos (orange line), the unloaded MOF (blue line) and the pattern generated from theoretical data (red line).

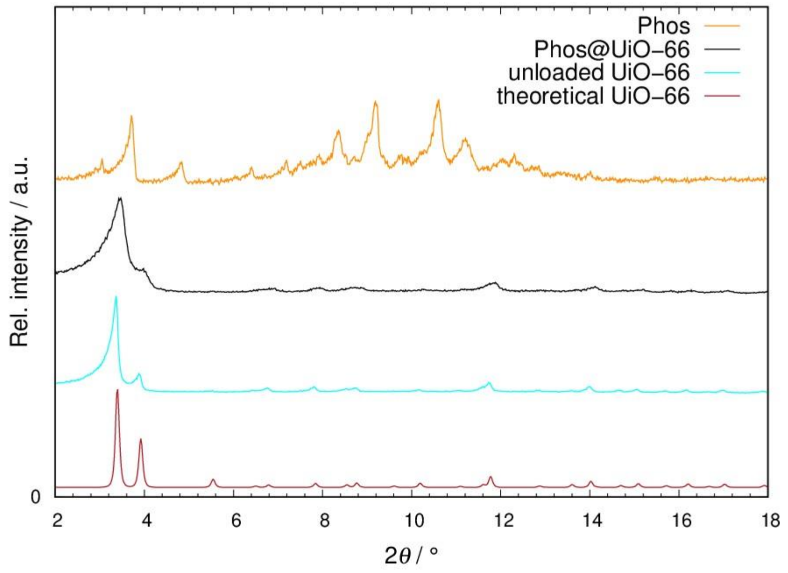

Figure S4. PXRD patterns of Phos@UiO-66 (**7**) (black line) in comparison to pure Phos (orange line), the unloaded MOF (blue line) and the pattern generated from theoretical data (red line).

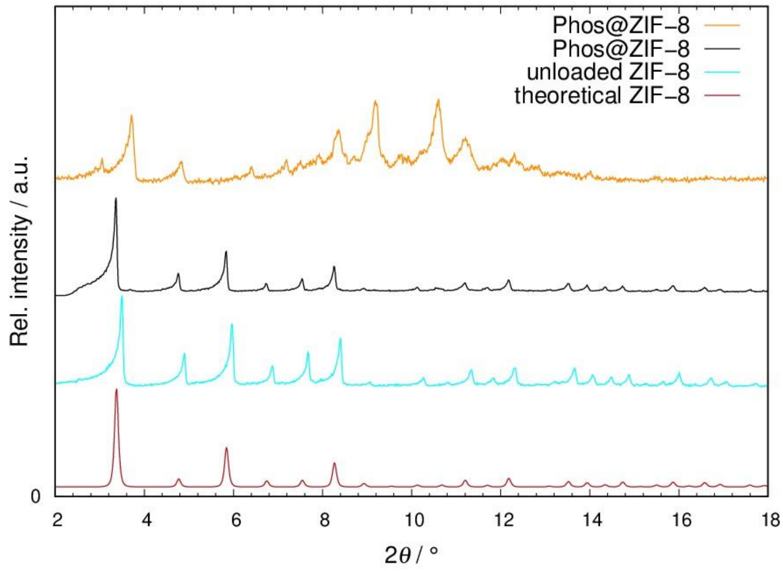

Figure S5. PXRD patterns of Phos@ZIF-8 (**8**) (black line) in comparison to pure Phos (orange line), the unloaded MOF (blue line) and the pattern generated from theoretical data (red line).

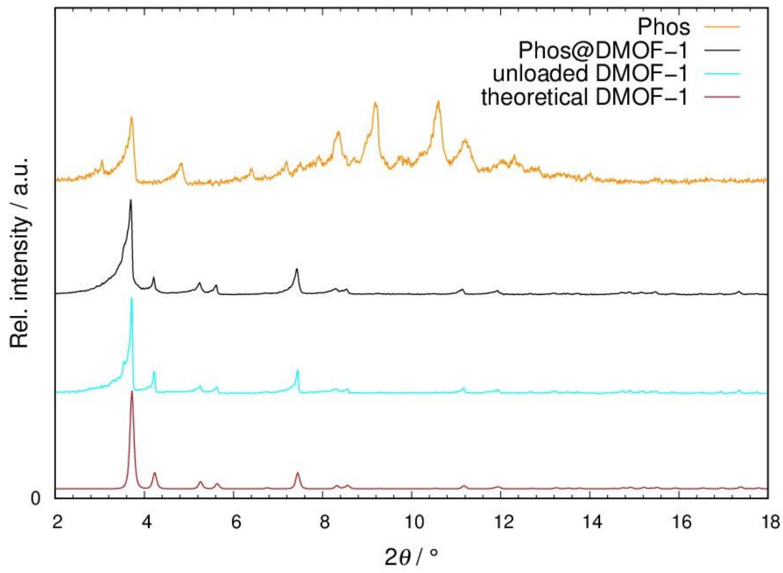

Figure S6. PXRD patterns of Phos@DMOF-1 (**9**) (black line) in comparison to pure Phos (orange line), the unloaded MOF (blue line) and the pattern generated from theoretical data (red line).

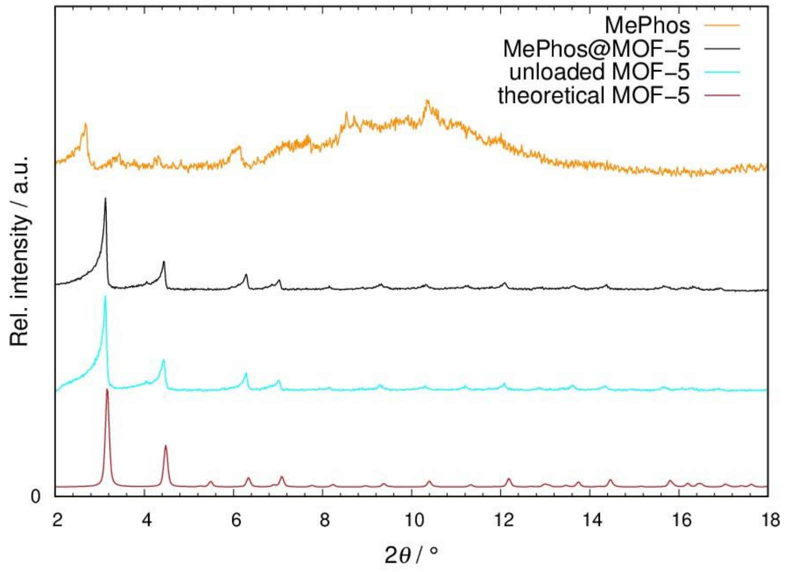

Figure S7. PXRD patterns of MePhos@MOF-5 (**10**) (black line) in comparison to pure MePhos (orange line), the unloaded MOF (blue line) and the pattern generated from theoretical data (red line).

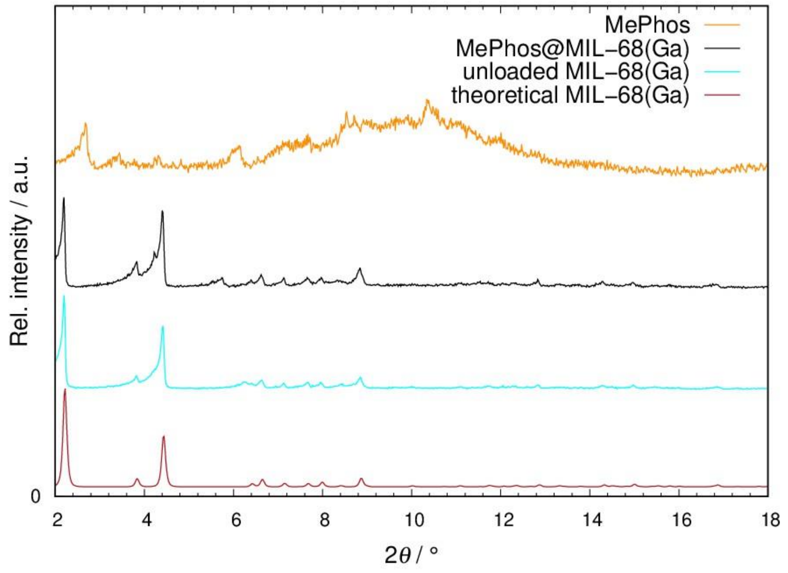

Figure S8. PXRD patterns of MePhos@MIL-68(Ga) (**15**) (black line) in comparison to pure MePhos (orange line), the unloaded MOF (blue line) and the pattern generated from theoretical data (red line).

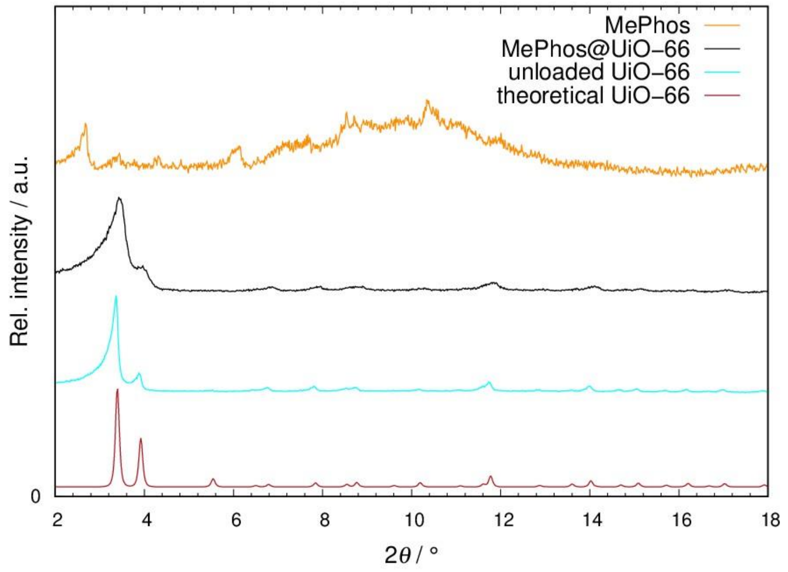

Figure S9. PXRD patterns of MePhos@UiO-66 (**16**) (black line) in comparison to pure MePhos (orange line), the unloaded MOF (blue line) and the pattern generated from theoretical data (red line).

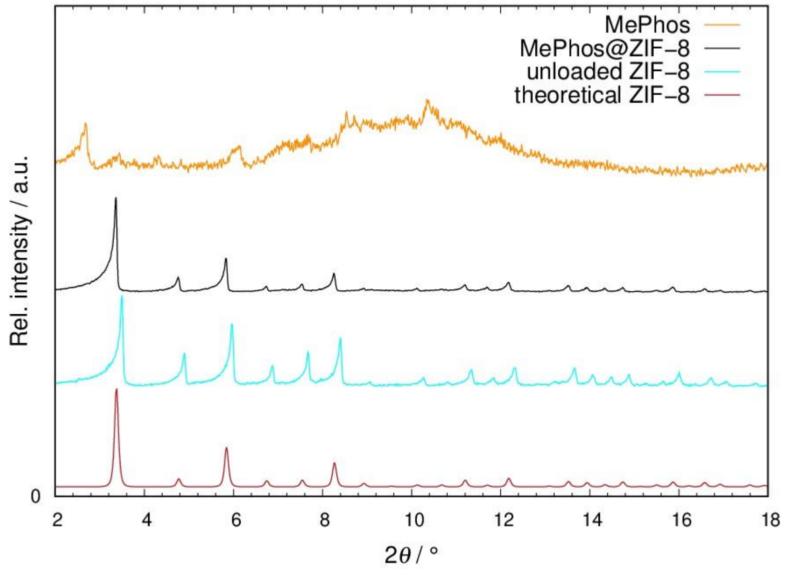

Figure S10. PXRD patterns of MePhos@ZIF-8 (**17**) (black line) in comparison to pure MePhos (orange line), the unloaded MOF (blue line) and the pattern generated from theoretical data (red line).

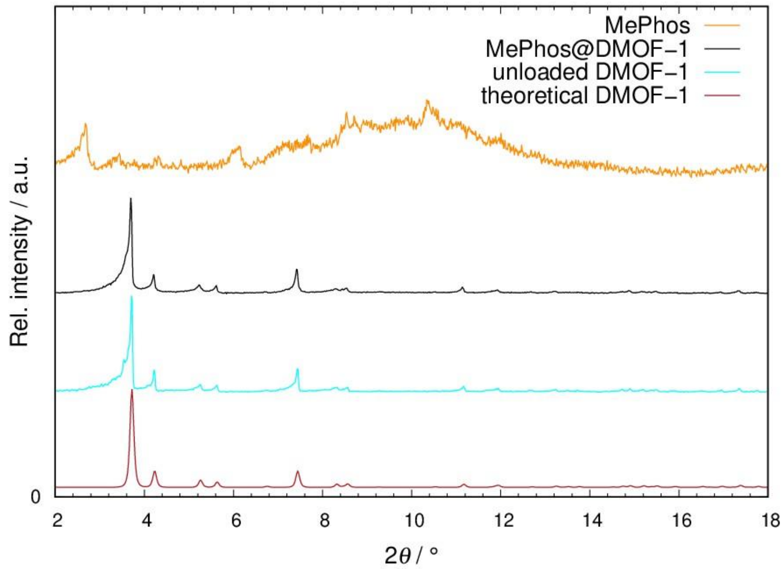

Figure S11. PXRD patterns of MePhos@DMOF-1 (**18**) (black line) in comparison to pure MePhos (orange line), the unloaded MOF (blue line) and the pattern generated from theoretical data (red line).

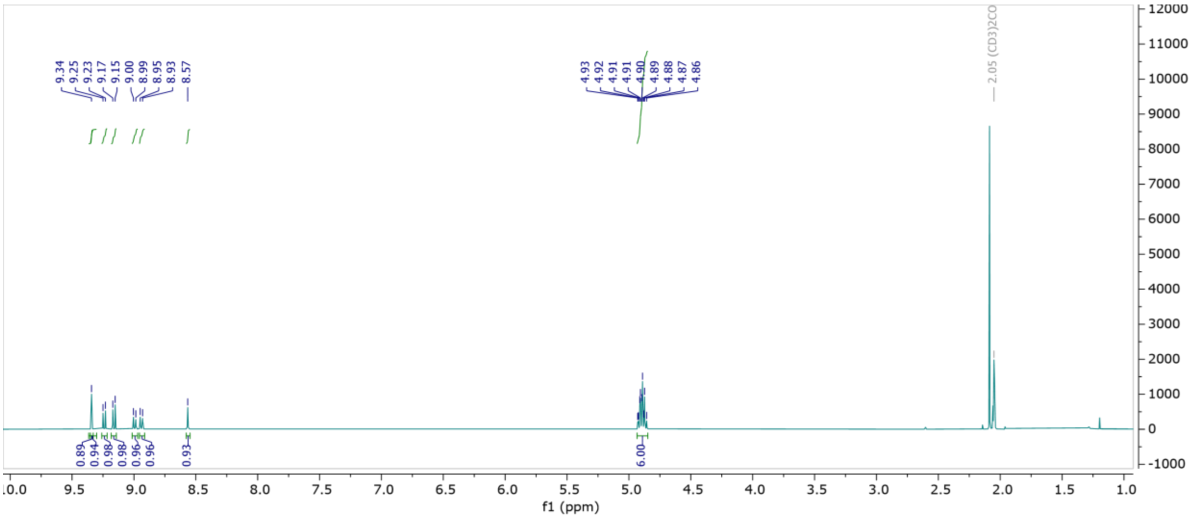

Figure S12. ^{1}H NMR spectrum of Phos in Acetone-d_6.

^{1}H NMR (500 MHz, Acetone) δ 9.34 (s, 1H), 9.24 (d, *J*= 9.7 Hz, 1H), 9.16 (d, *J*= 9.6 Hz, 1H), 8.99 (d, *J*= 9.5 Hz, 1H), 8.94 (d, *J*= 9.7 Hz, 1H), 8.57 (s, 1H), 4.93 – 4.85 (m, 6H).

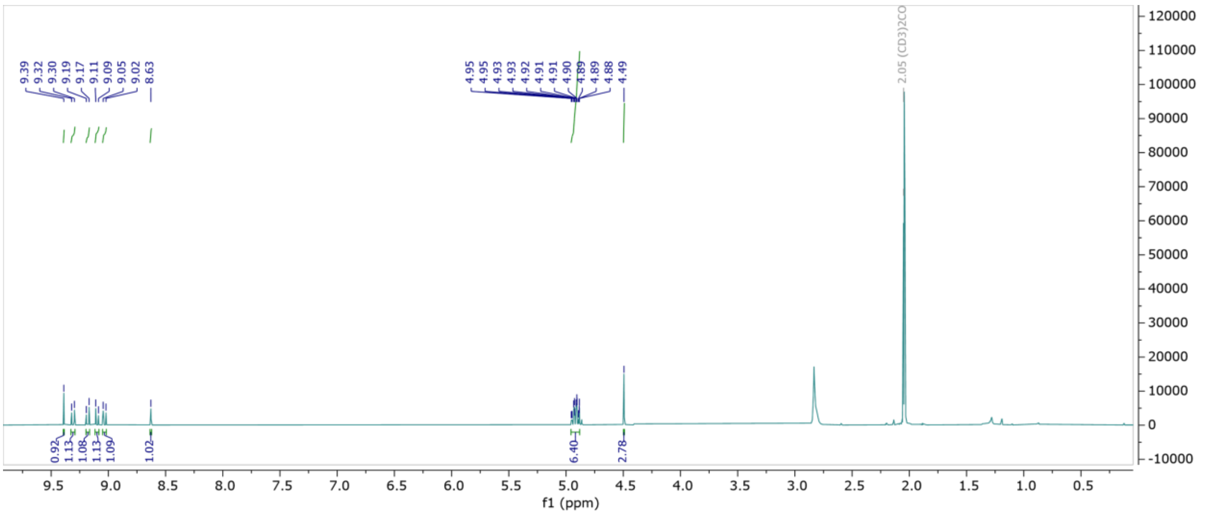

Figure S13. ^{1}H NMR spectrum of MePhos in Acetone-d_6.

^{1}H NMR (400 MHz, Acetone) δ 9.39 (s, 1H), 9.31 (d, *J*= 9.8 Hz, 1H), 9.18 (d, *J*= 9.6 Hz, 1H), 9.10 (d, *J*= 9.6 Hz, 1H), 9.03 (d, *J*= 9.8 Hz, 1H), 8.63 (s, 1H), 4.98 – 4.81 (m, 6H), 4.49 (s, 3H).

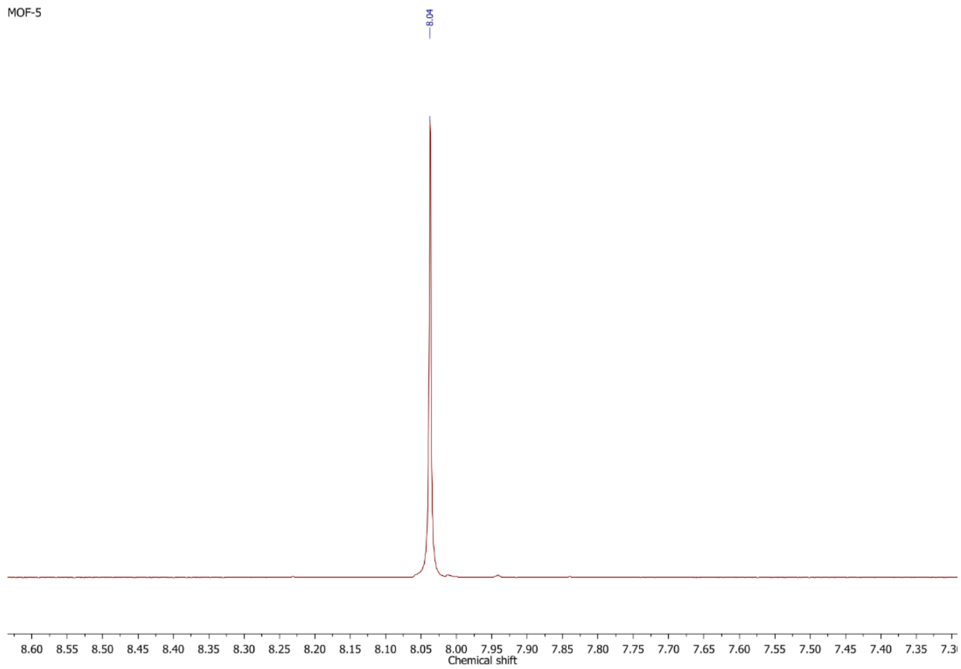

Figure S14. ^{1}H NMR spectrum of MOF-5 digested in DMSO-d_6 and DCl.

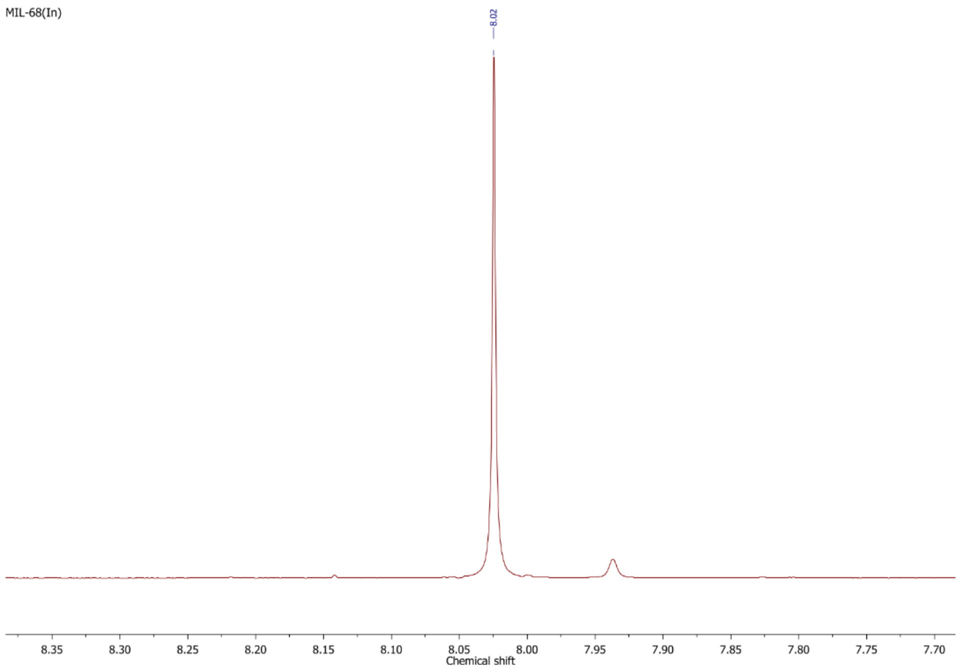

Figure S15. ^{1}H NMR spectrum of MIL-68(In) digested in DMSO-d_6 and DCl.

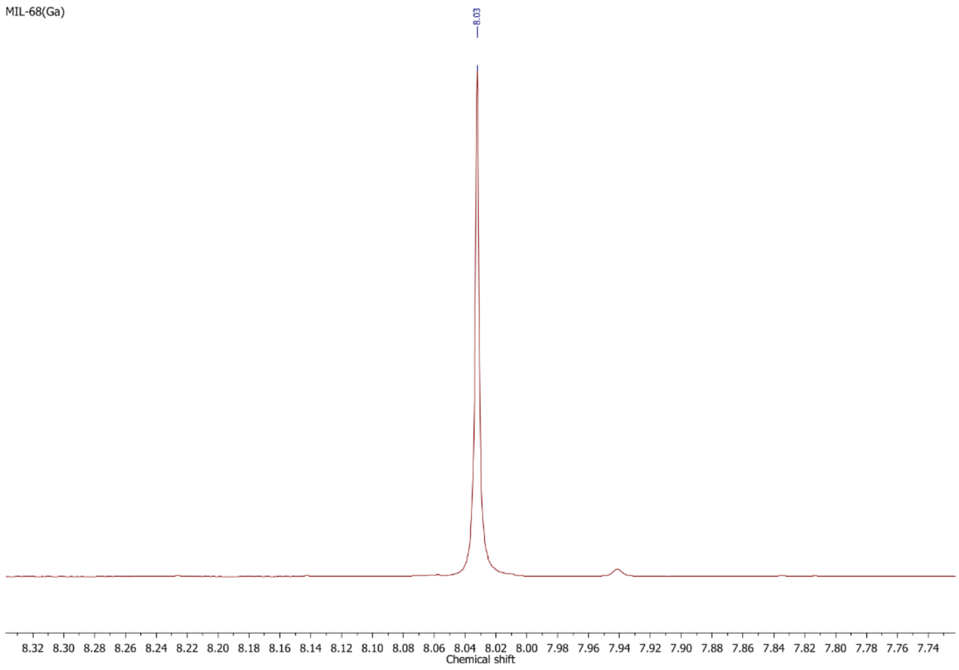

Figure S16. ^{1}H NMR spectrum of MIL-68(Ga) digested in DMSO-d_6 and DCl.

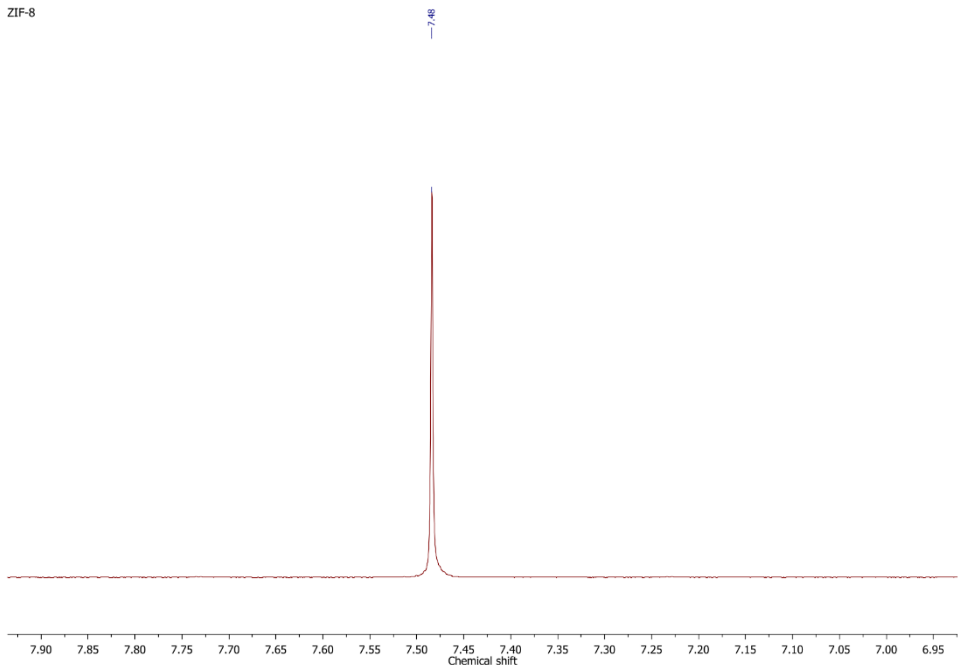

Figure S17. ^{1}H NMR spectrum of ZIF-8 digested in DMSO-d_6 and DCl.

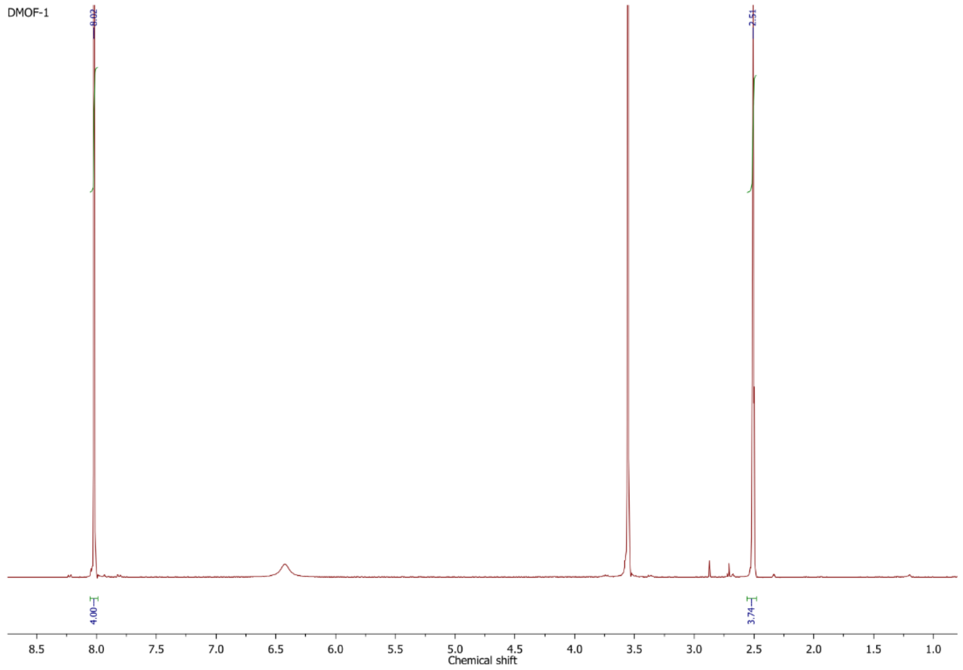

Figure S18. ^{1}H NMR spectrum of DMOF-1 digested in DMSO-d_6 and DCl.

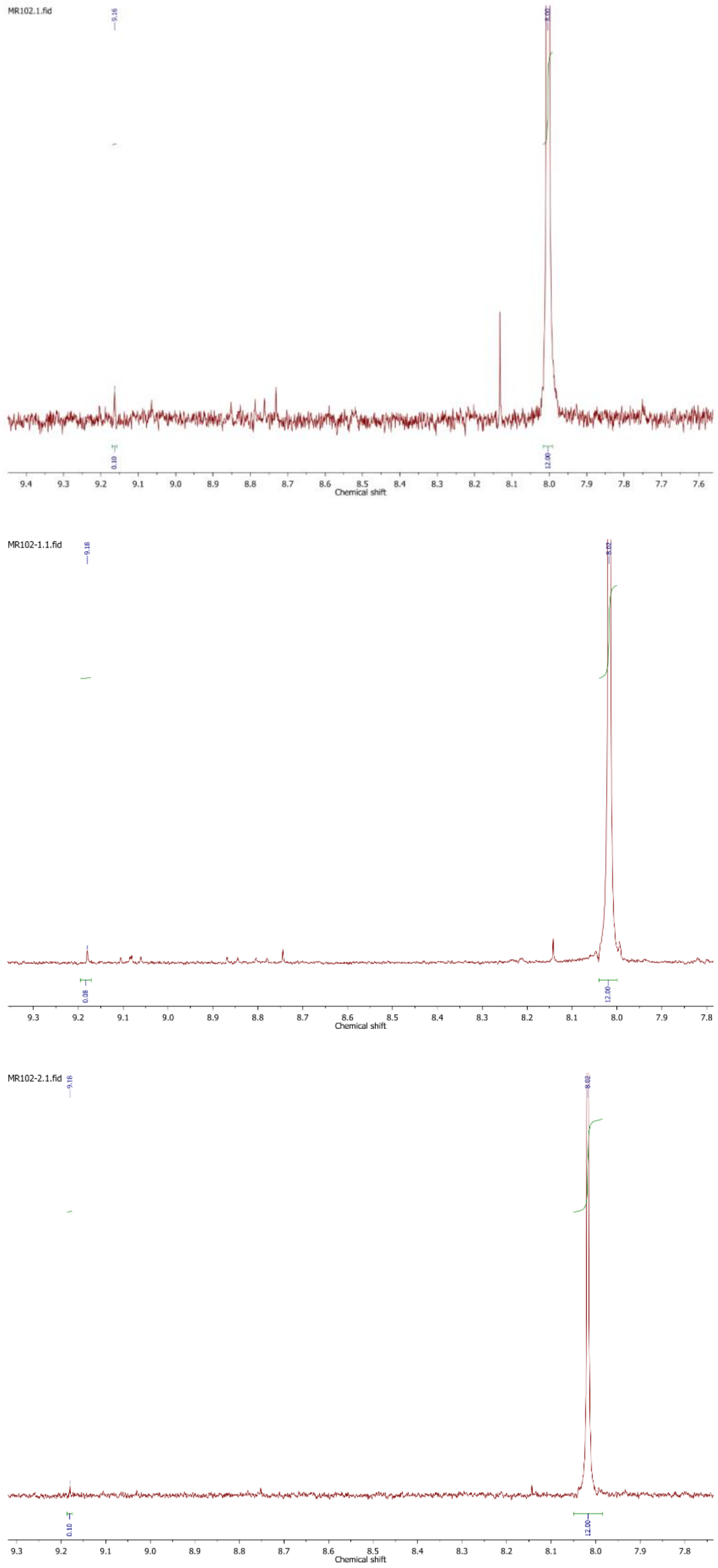

Figure S19. ^{1}H NMR spectrum of Phos@MOF-5 (**1**) digested in DMSO-d_6 and DCl.

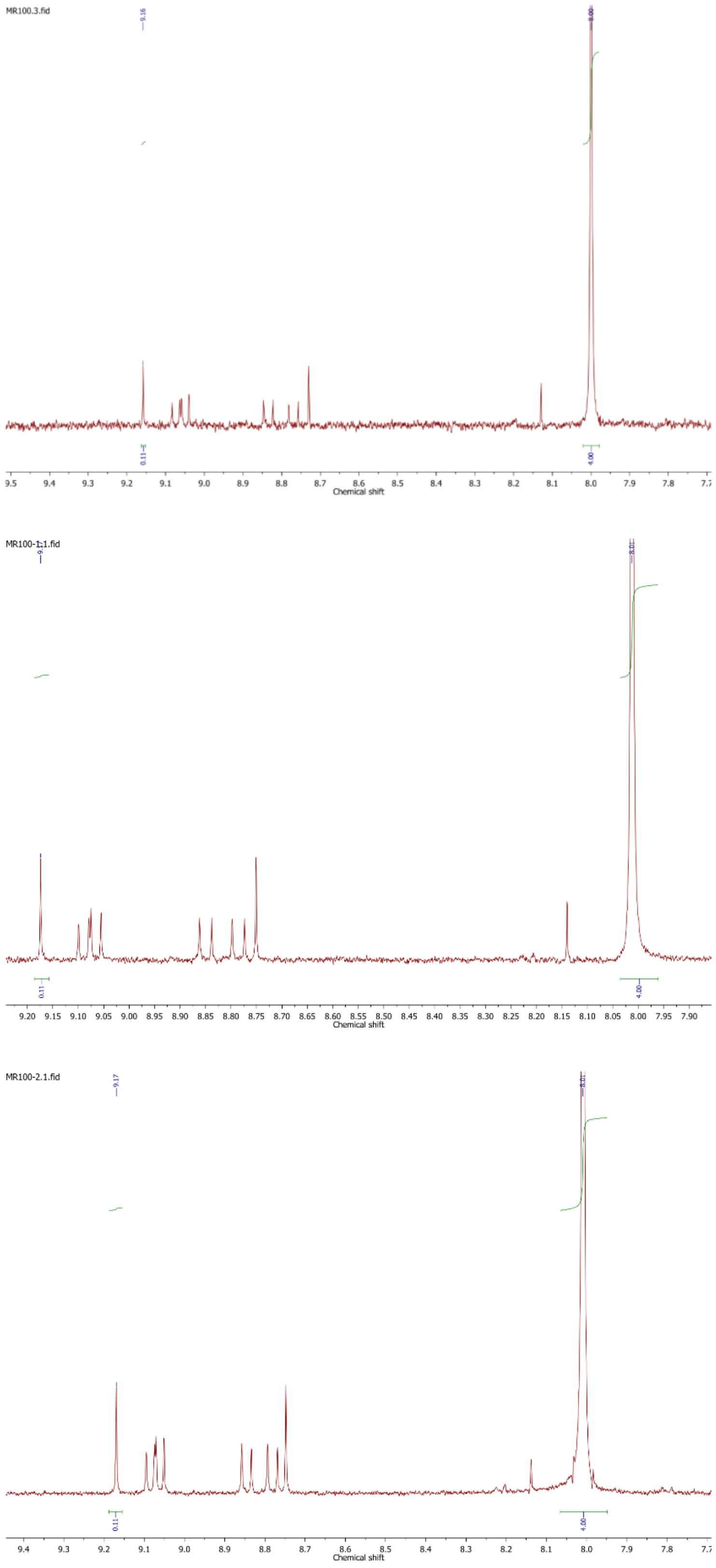

Figure S20. ^{1}H NMR spectrum of Phos@MIL-68(In) (**2**) digested in DMSO-d_6 and DCl.

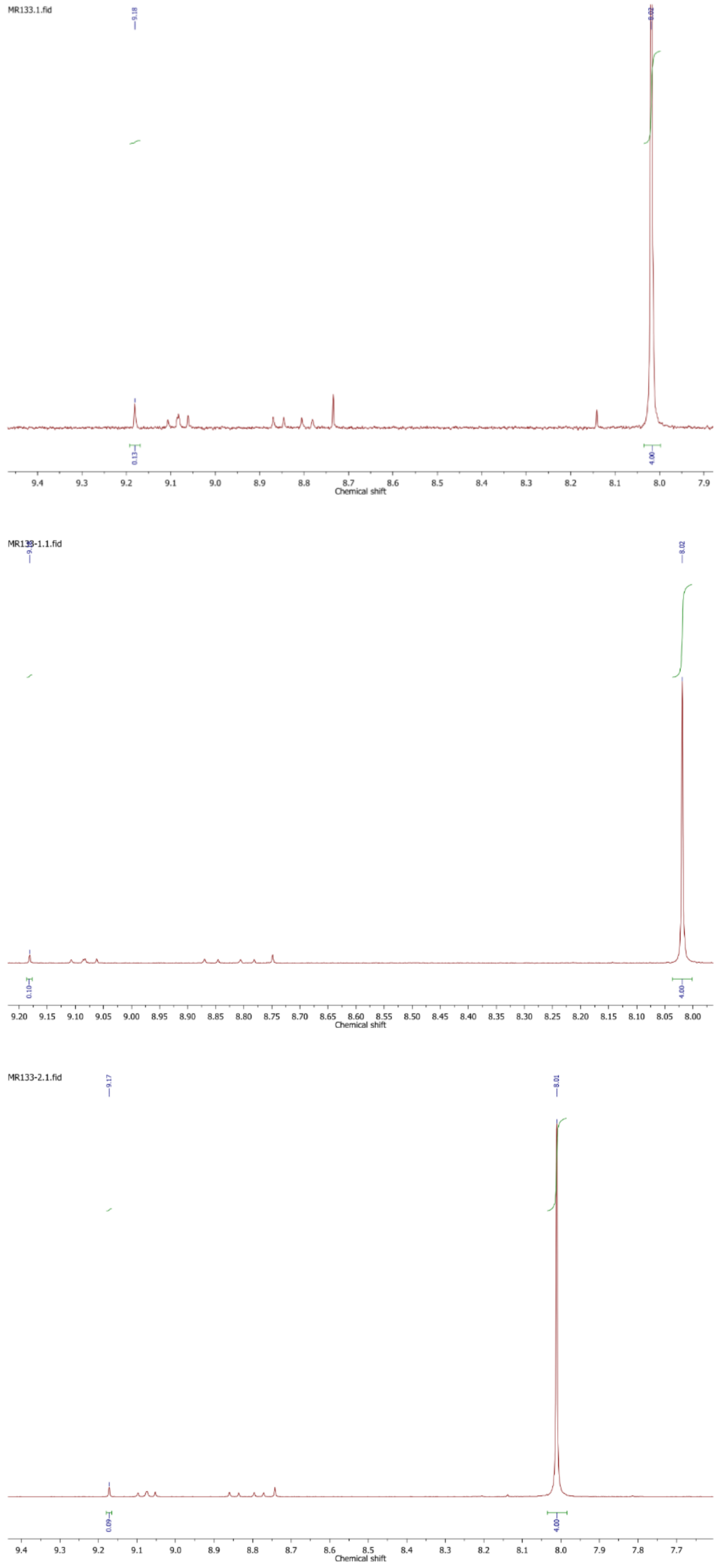

Figure S21. ^{1}H NMR spectrum of Phos@MIL-68(Ga) (**6**) digested in DMSO-d_6 and DCl.

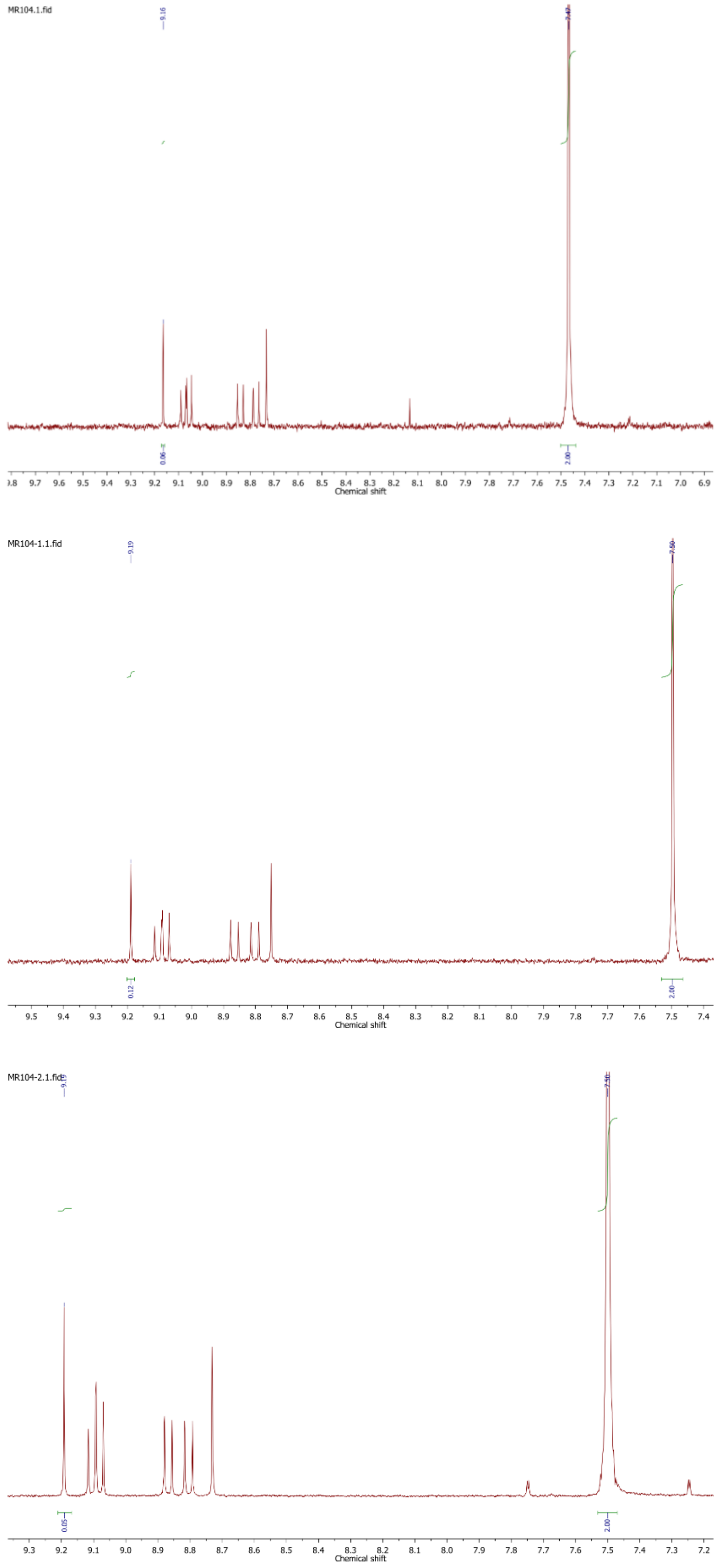

Figure S22. ^{1}H NMR spectrum of Phos@ZIF-8 (**8**) digested in DMSO-d_6 and DCl.

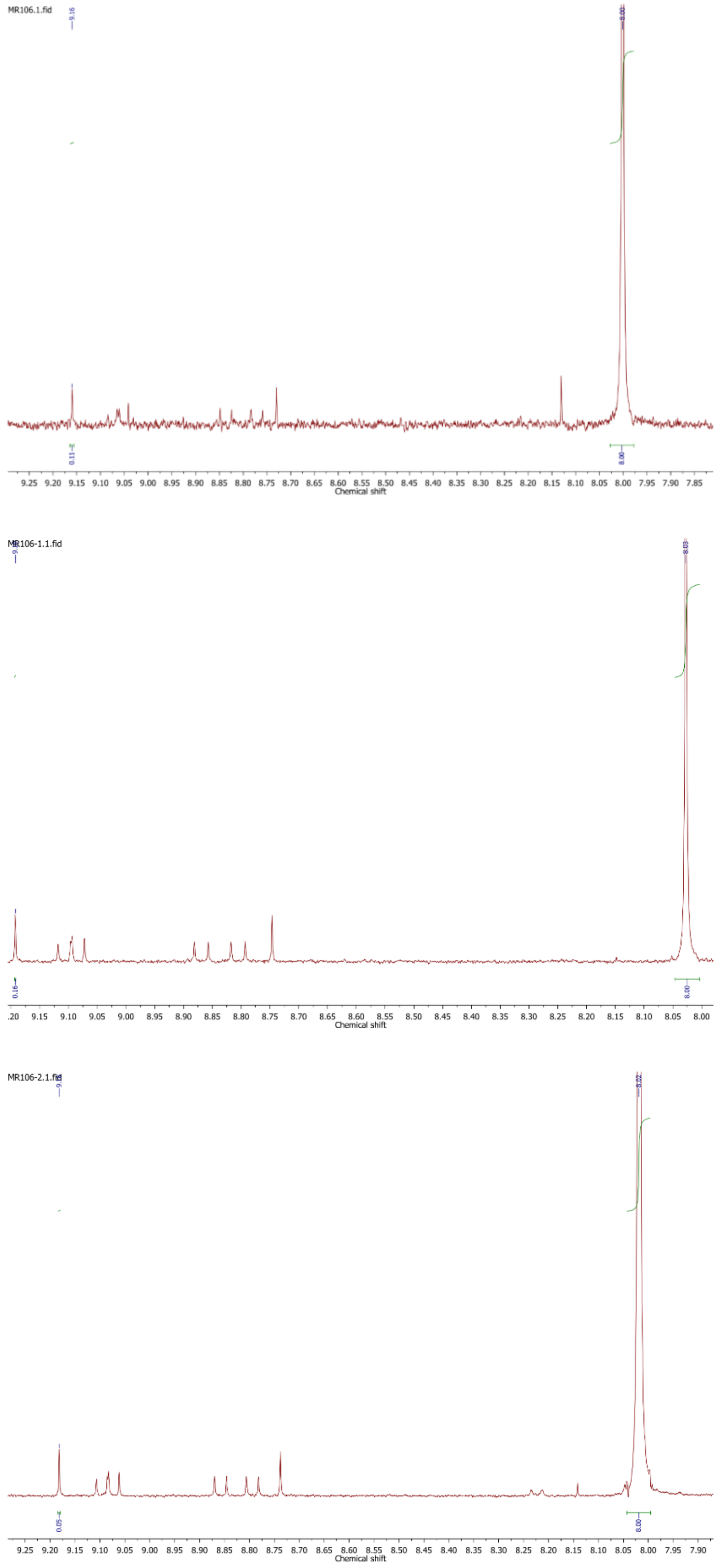

Figure S23. ^{1}H NMR spectrum of Phos@DMOF-1 (**9**) digested in DMSO-d_6 and DCl.

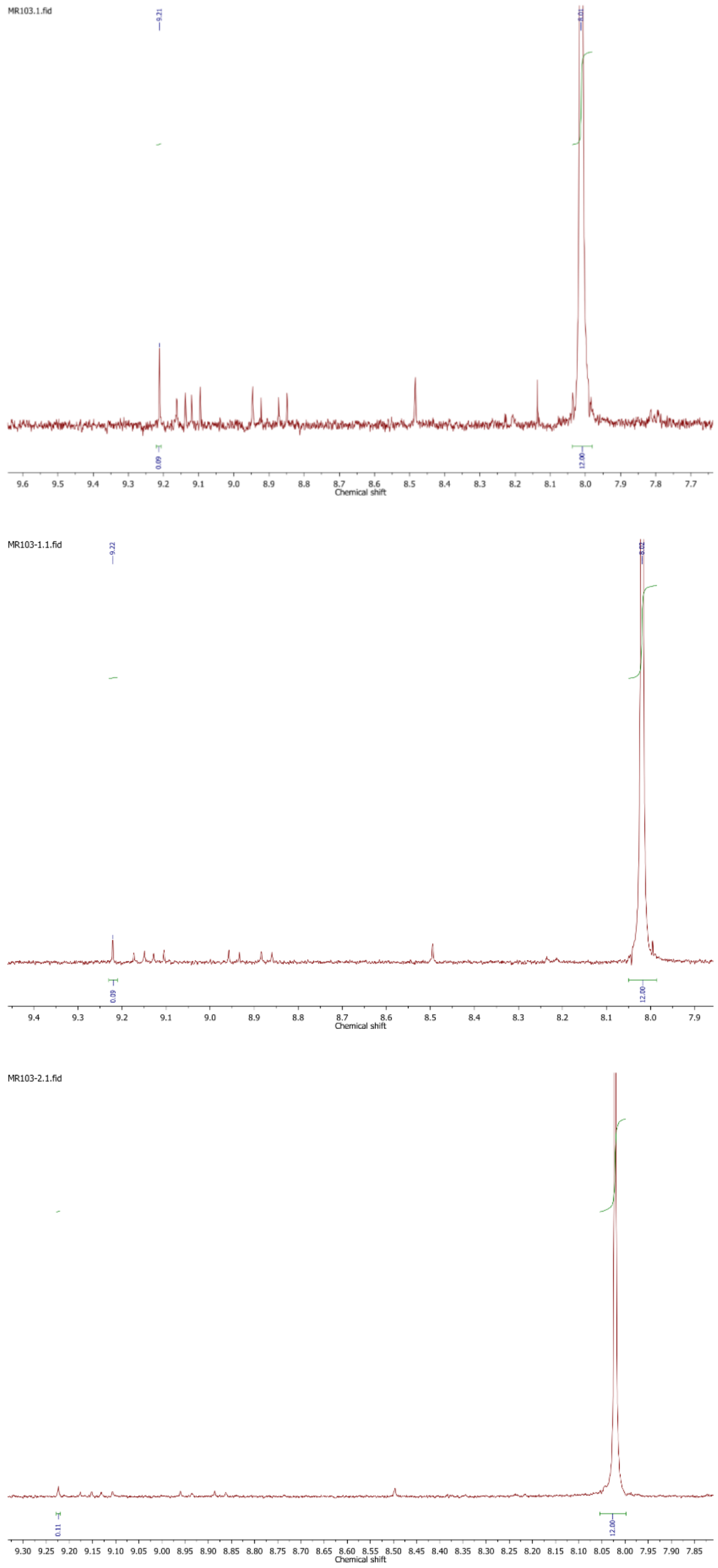

Figure S24. ^{1}H NMR spectrum of MePhos@MOF-5 (**10**) digested in DMSO-d_6 and DCl.

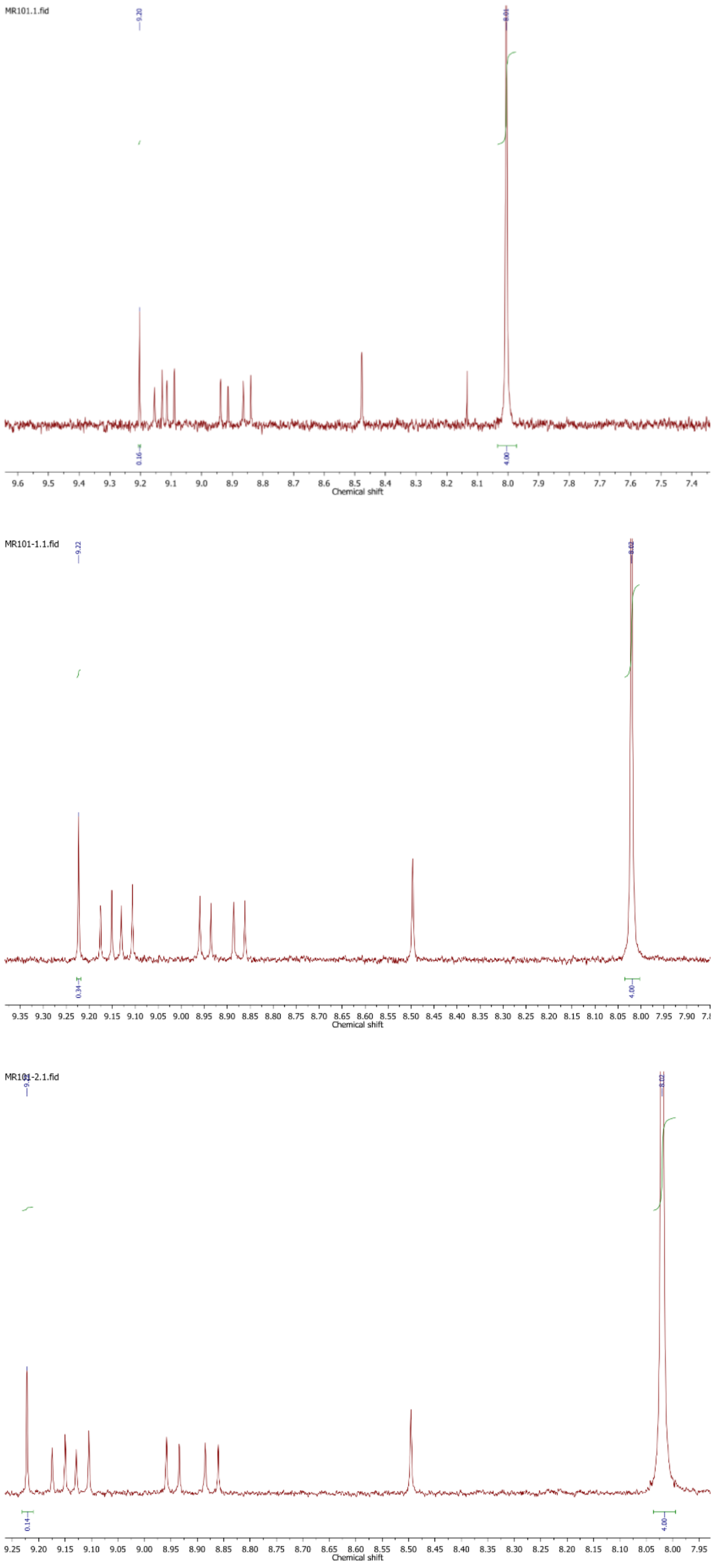

Figure S25. ^{1}H NMR spectrum of MePhos@MIL-68(In) (**11**) digested in DMSO-d_6 and DCl.

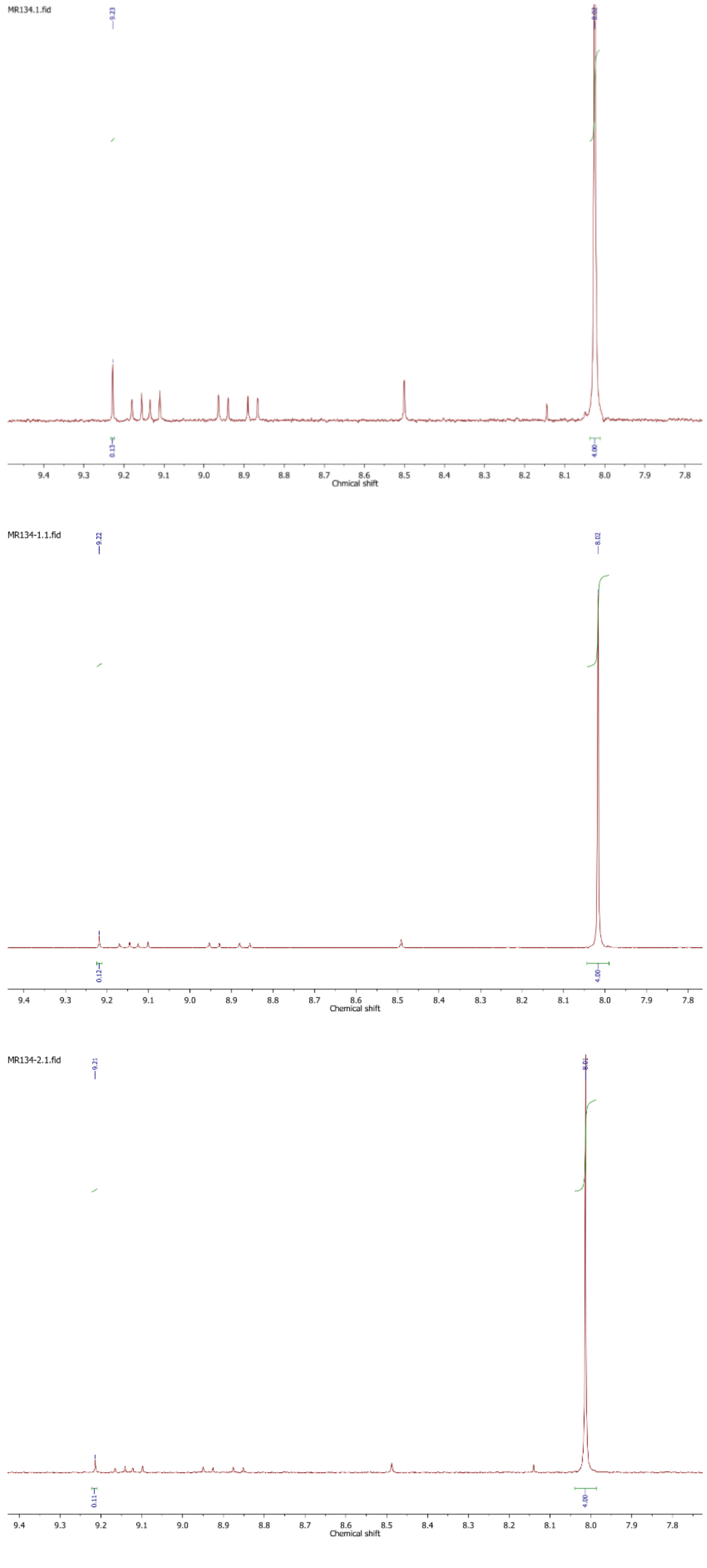

Figure S26. ^{1}H NMR spectrum of MePhos@MIL-68(Ga) (**15**) digested in DMSO-d_6 and DCl.

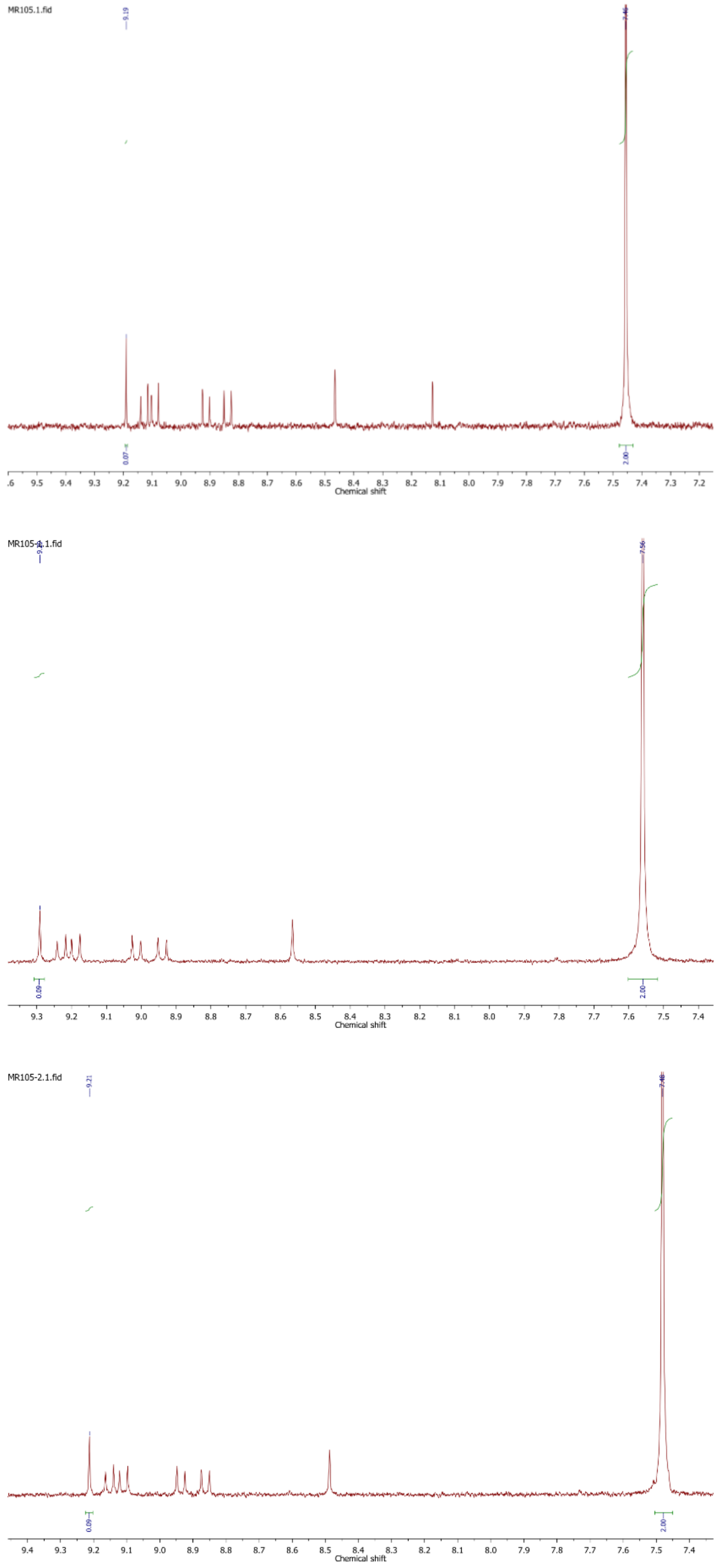

Figure S27. ^{1}H NMR spectrum of MePhos@ZIF-8 (**17**) digested in DMSO-d_6 and DCl.

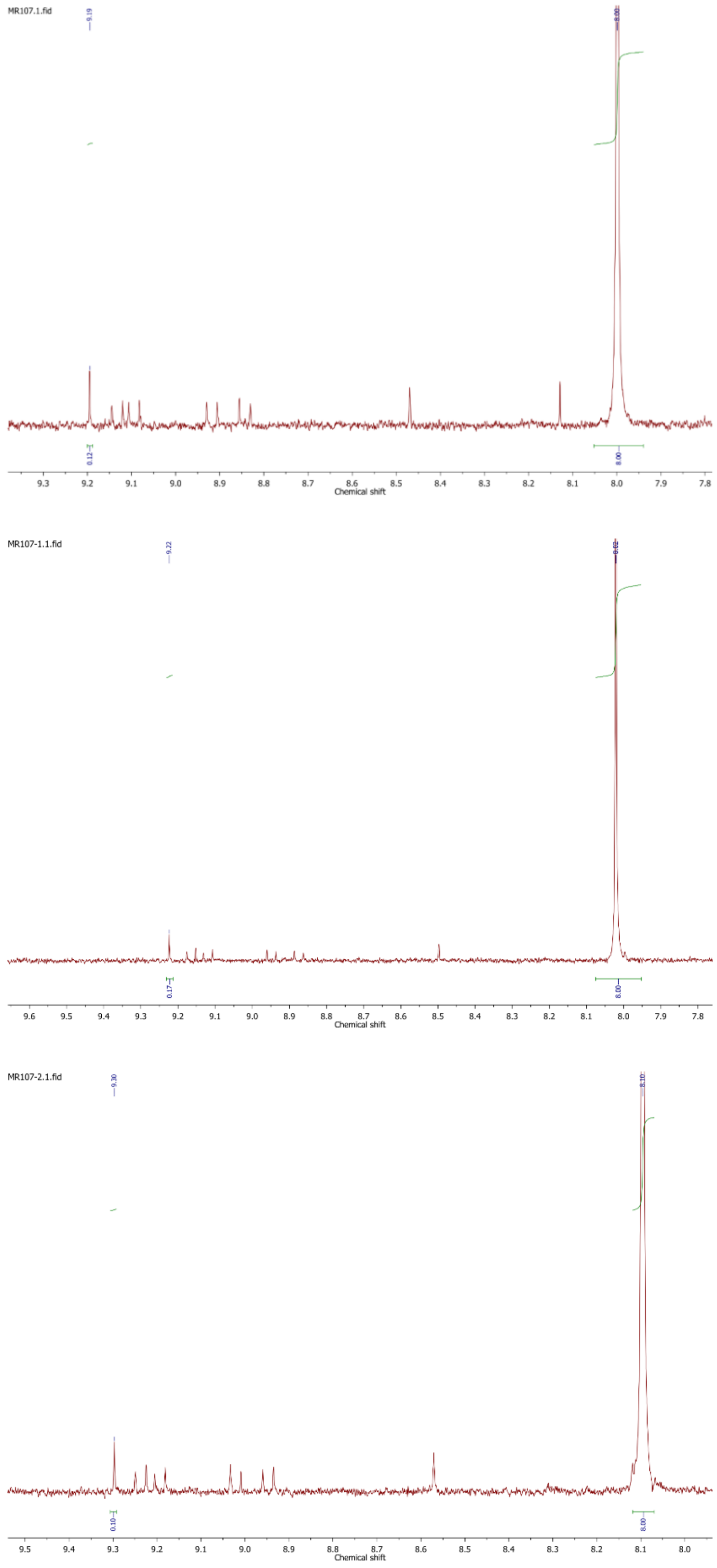

Figure S28. ^{1}H NMR spectrum of MePhos@DMOF-1 (**18**) digested in DMSO-d_6 and DCl.

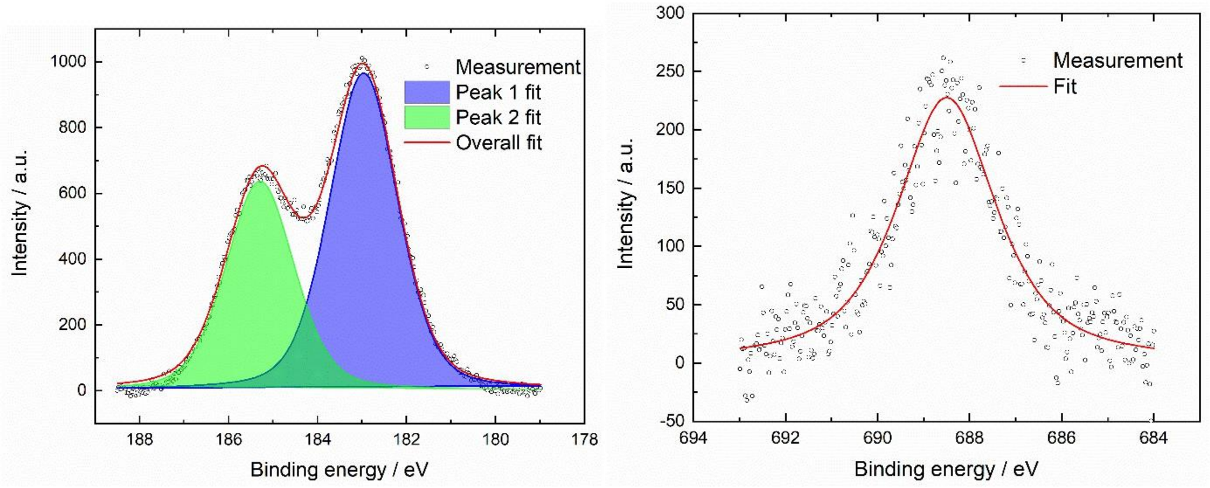

Figure S29. XPS spectrum of the Zr $3d_{5/2}$ (left, blue area), Zr $3d_{3/2}$ (left, green area) and F 1s peak (right) of Phos@UiO-66 (**8**) with experimental data (black dots) and fit (red line).

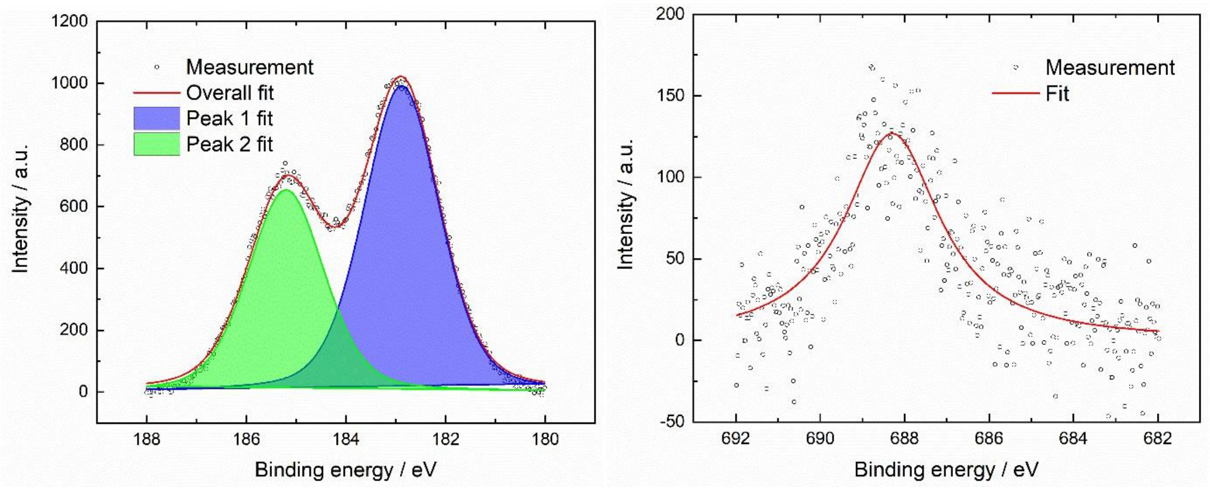

Figure S30. XPS spectrum of the Zr $3d_{5/2}$ (left, blue area), Zr $3d_{3/2}$ (left, green area) and F 1s peak (right) of MePhos@UiO-66 (**18**) with experimental data (black dots) and fit (red line).

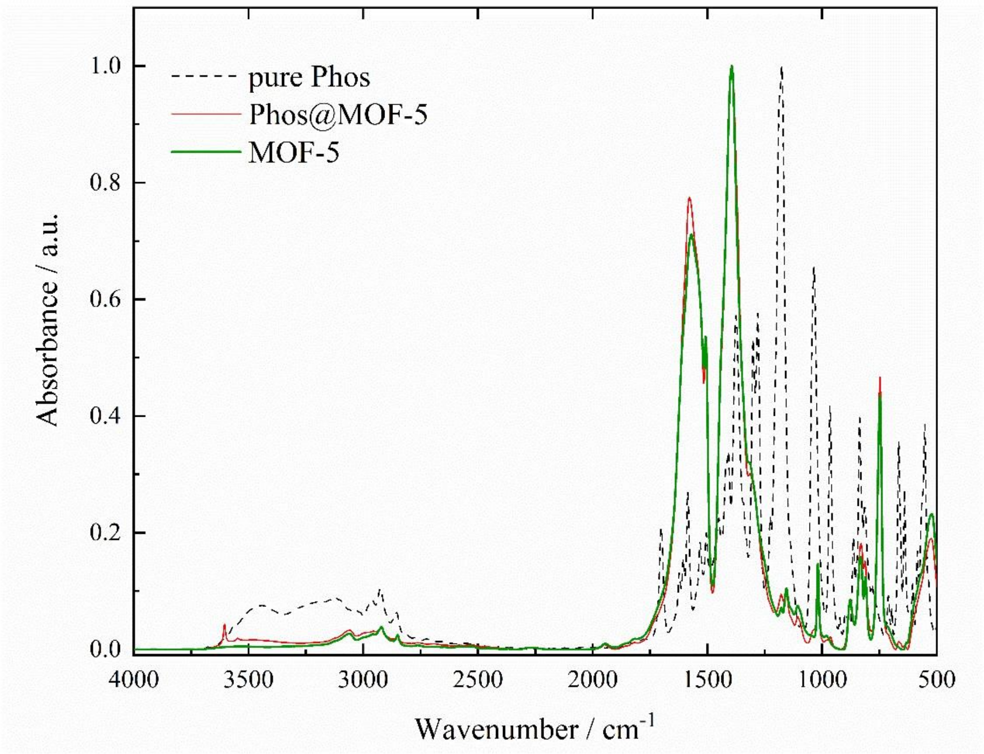

Figure S31. IR spectra of pure Phos (dashed black line), Phos@MOF-5 (red line) and MOF-5 (green line).

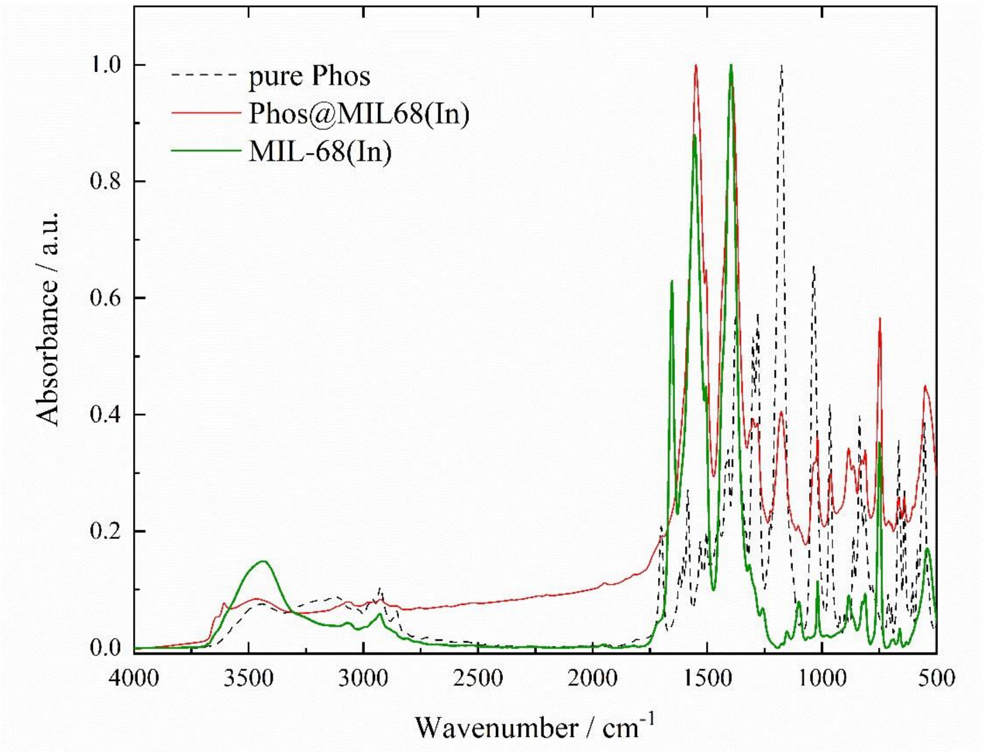

Figure S32. IR spectra of pure Phos (dashed black line), Phos@MIL-68(In) (red line) and MIL-68(In) (green line).

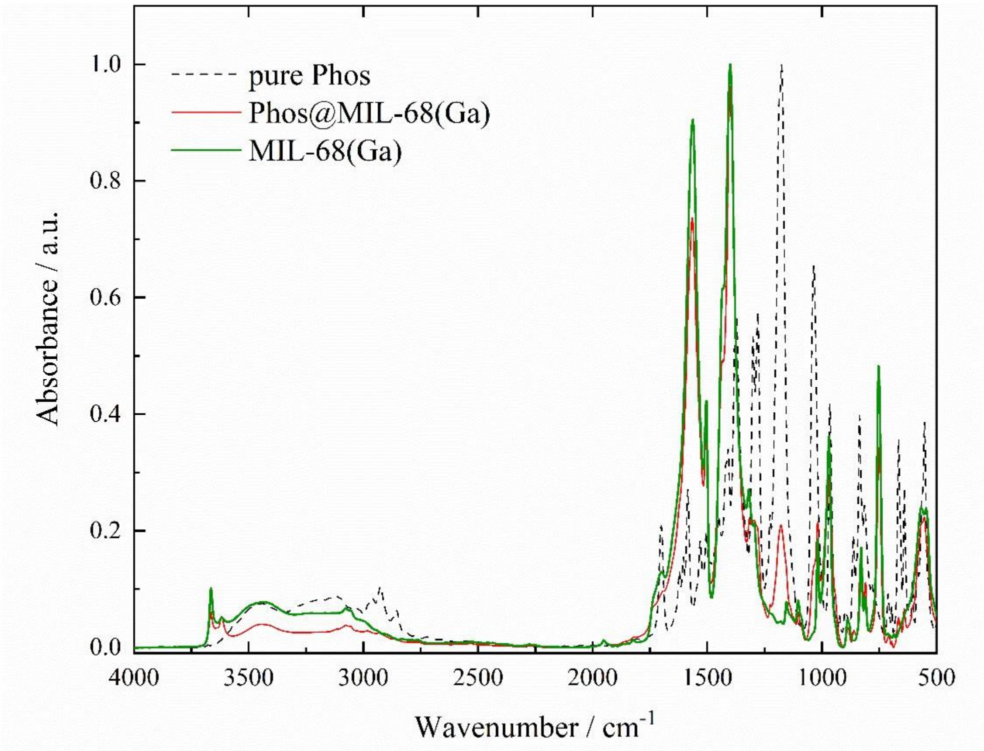

Figure S33. IR spectra of pure Phos (dashed black line), Phos@MIL-68(Ga) (red line) and MIL-68(Ga) (green line).

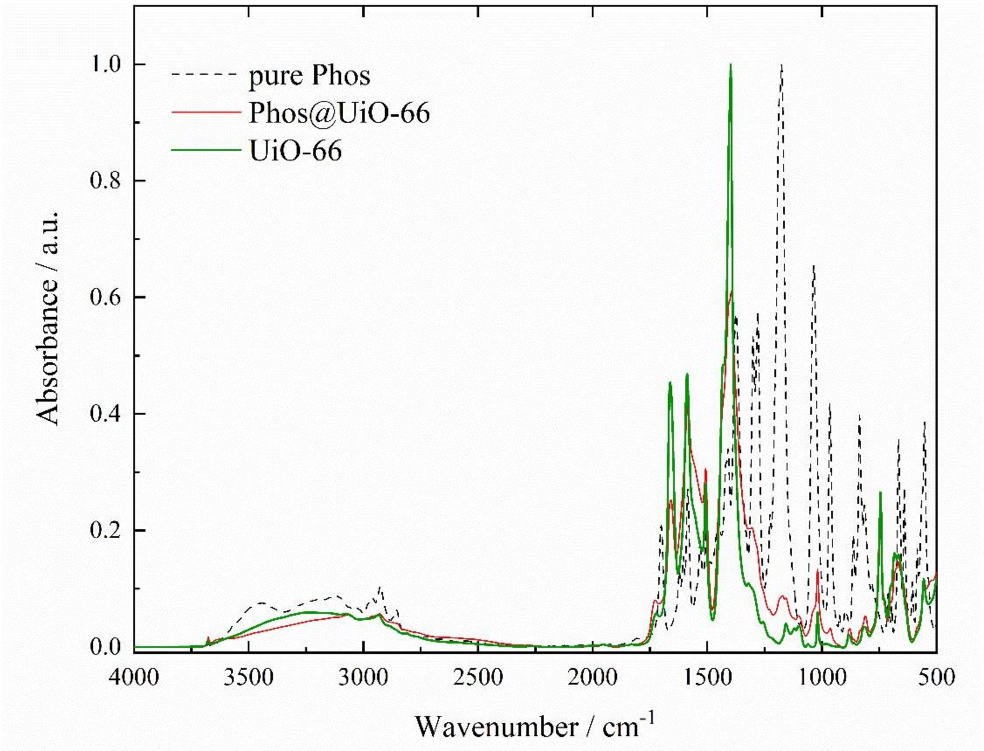

Figure S34. IR spectra of pure Phos (dashed black line), Phos@UiO-66 (red line) and UiO-66 (green line).

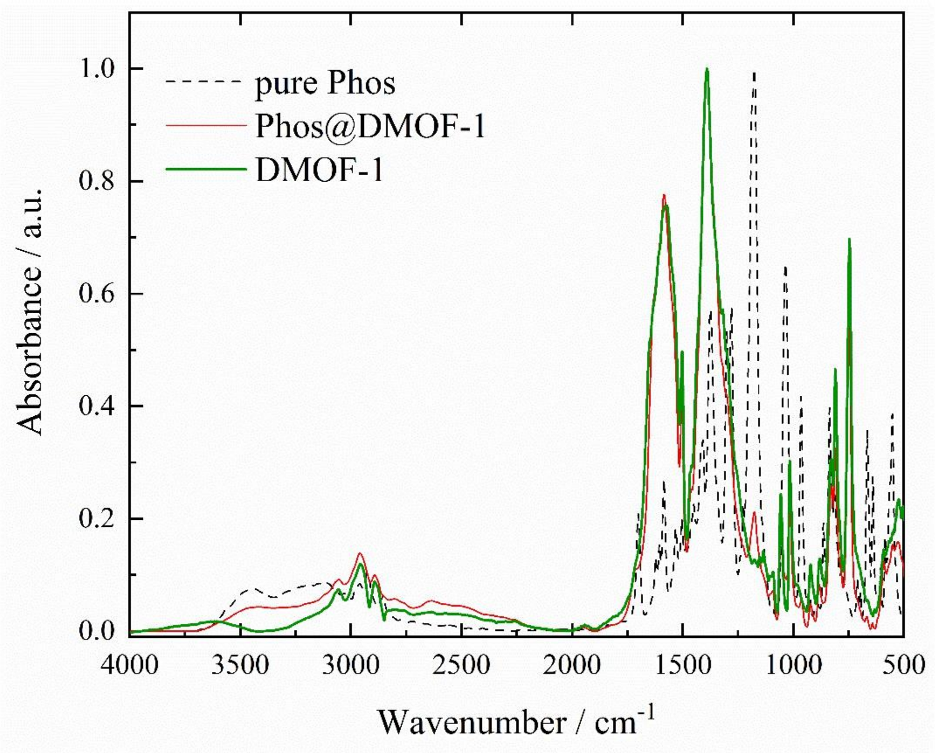

Figure S35. IR spectra of pure Phos (dashed black line), Phos@DMOF-1 (red line) and DMOF-1 (green line).

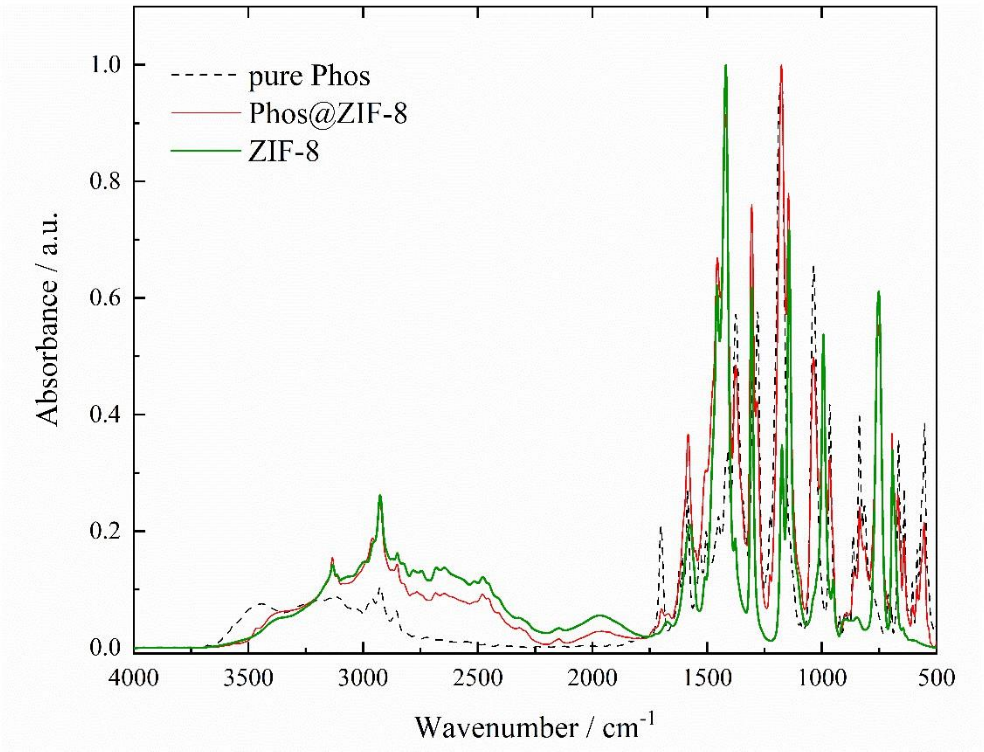

Figure S36. IR spectra of pure Phos (dashed black line), Phos@ZIF-8 (red line) and ZIF-8 (green line).

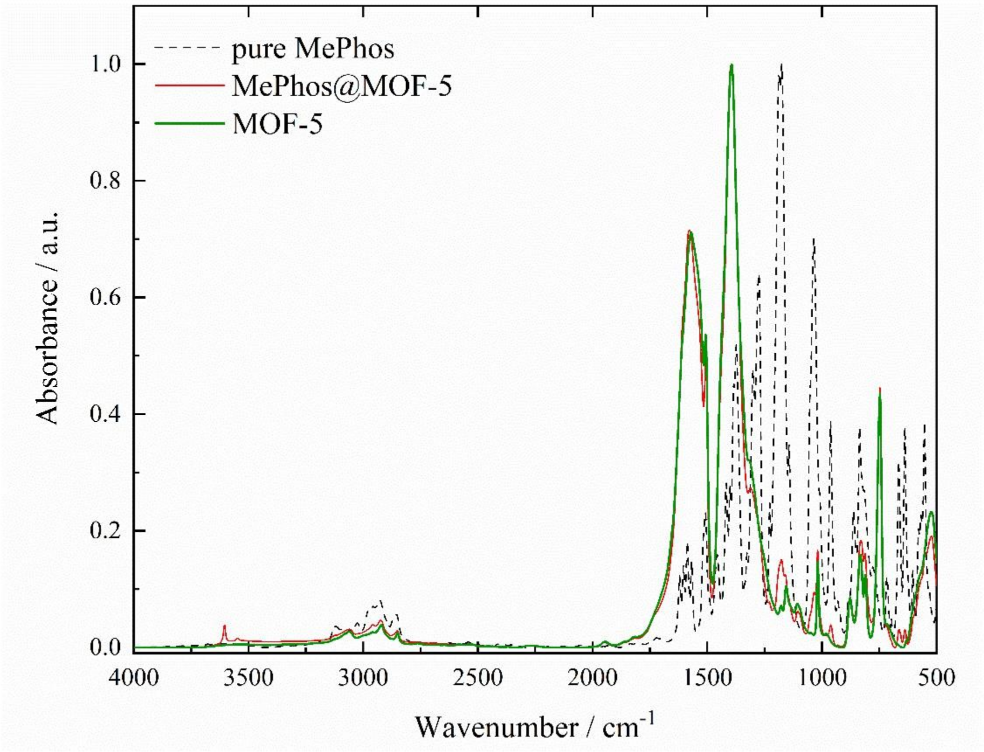

Figure S37. IR spectra of pure MePhos (dashed black line), MePhos@MOF-5 (red line) and MOF-5 (black line).

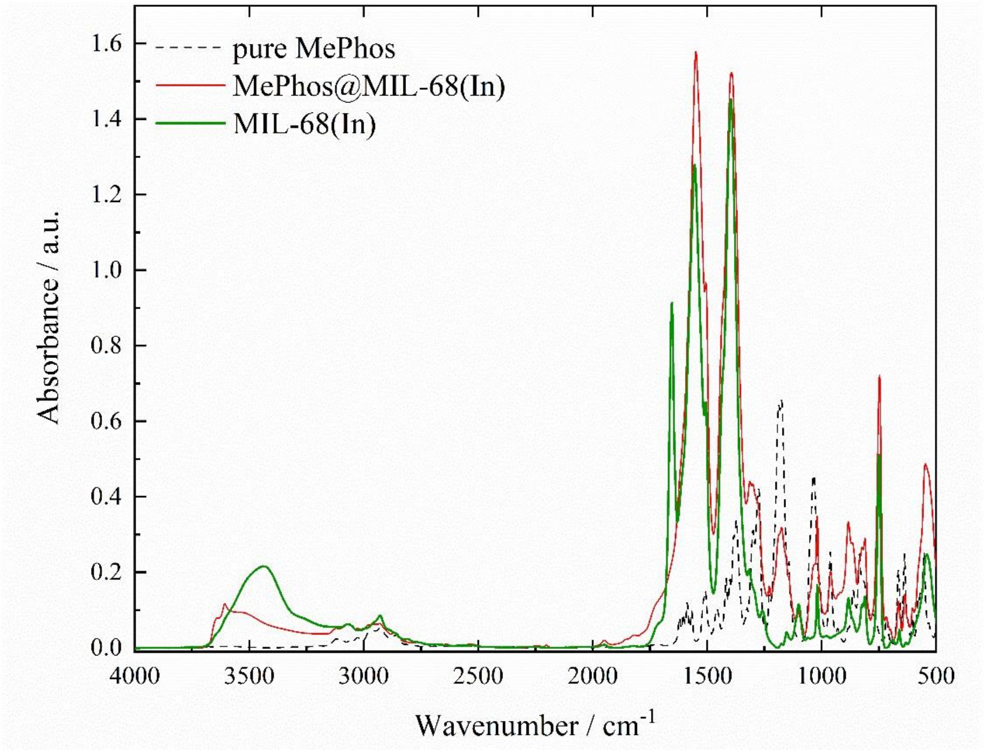

Figure S38. IR spectra of pure MePhos (dashed black line), MePhos@MIL-68(In) (**11**) (red line) and MIL-68(In) (black line).

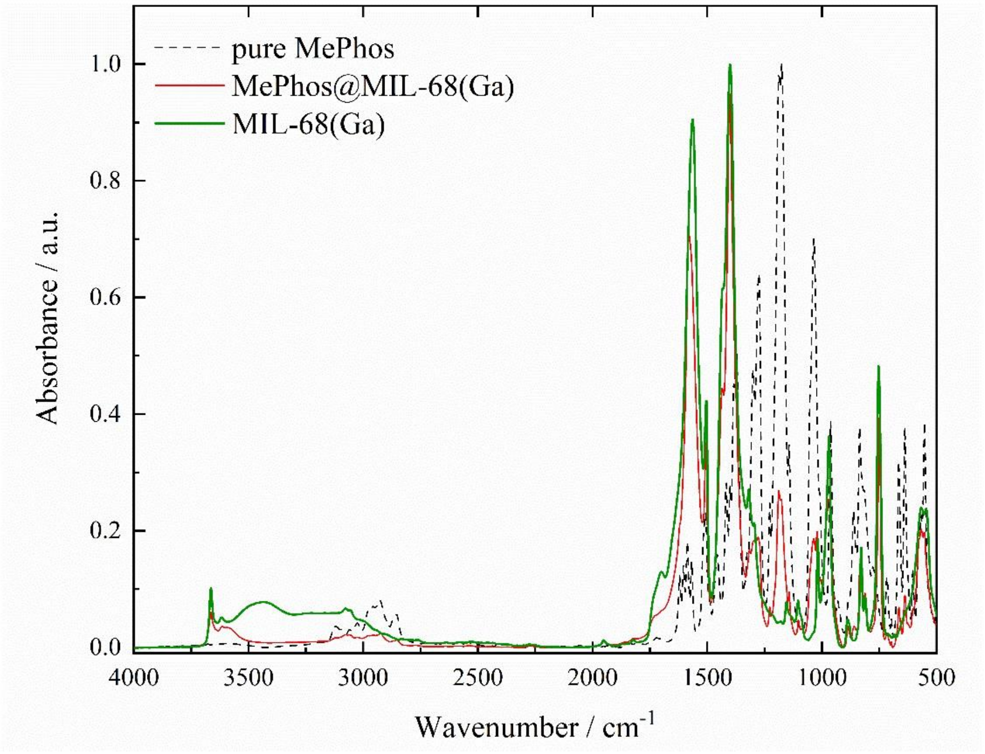

Figure S39. IR spectra of pure MePhos (dashed black line), MePhos@MIL-68(Ga) (red line) and MIL-68(Ga) (green line).

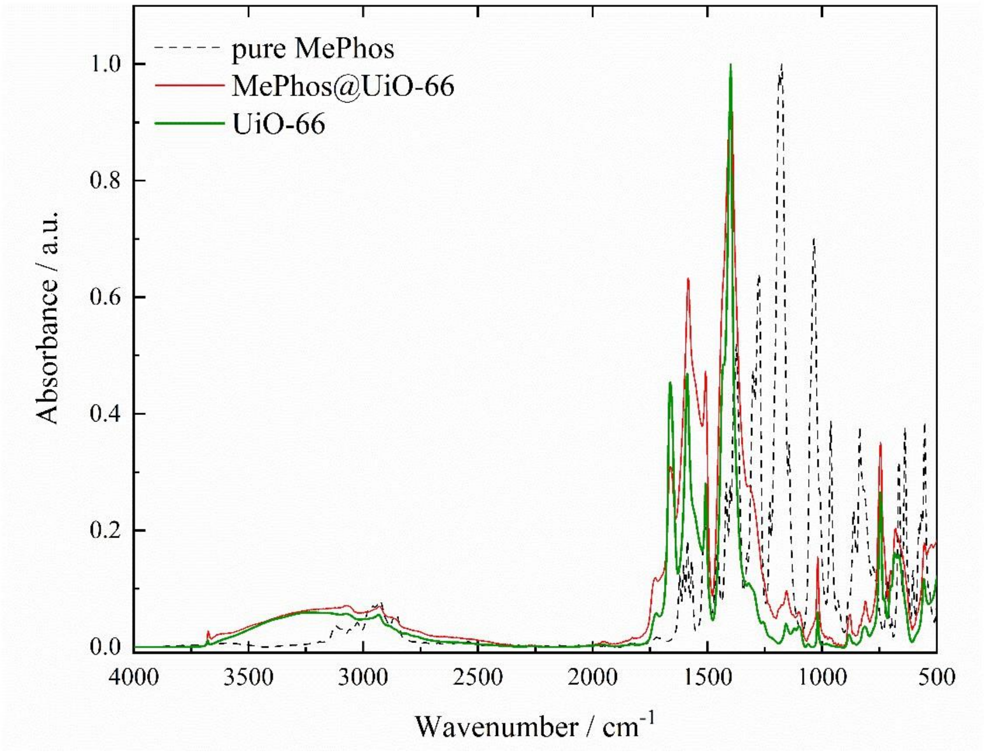

Figure S40. IR spectra of pure MePhos (dashed black line), MePhos@UiO-66 (red line) and UiO-66 (green line).

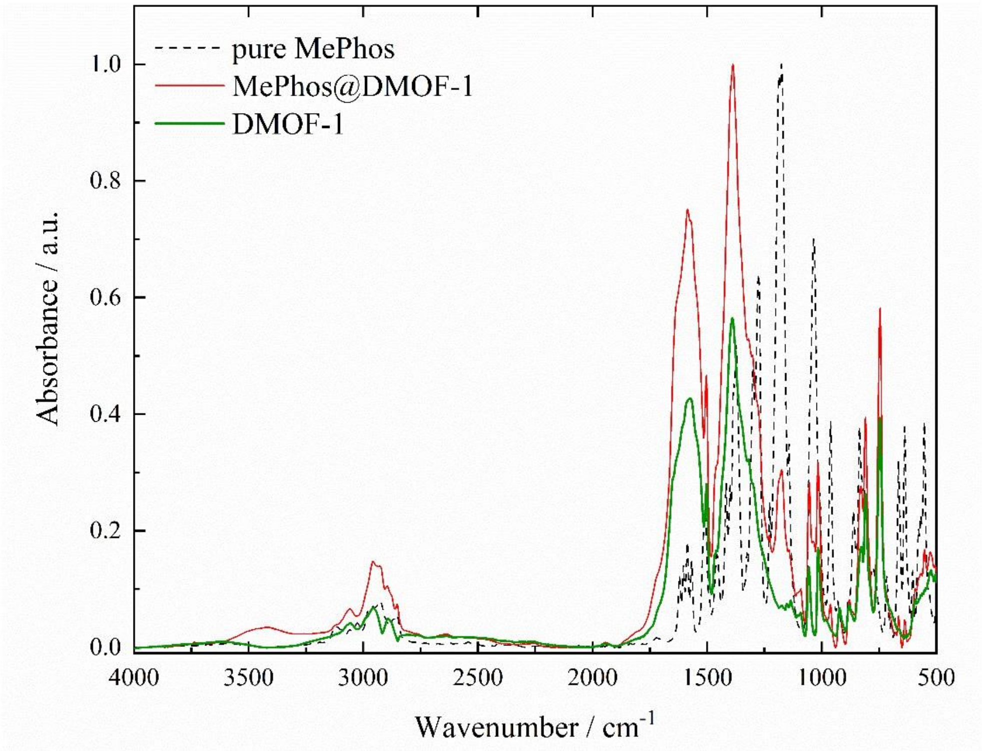

Figure S41. IR spectra of pure MePhos (dashed black line), MePhos@DMOF-1 (red line) and DMOF-1 (green line).

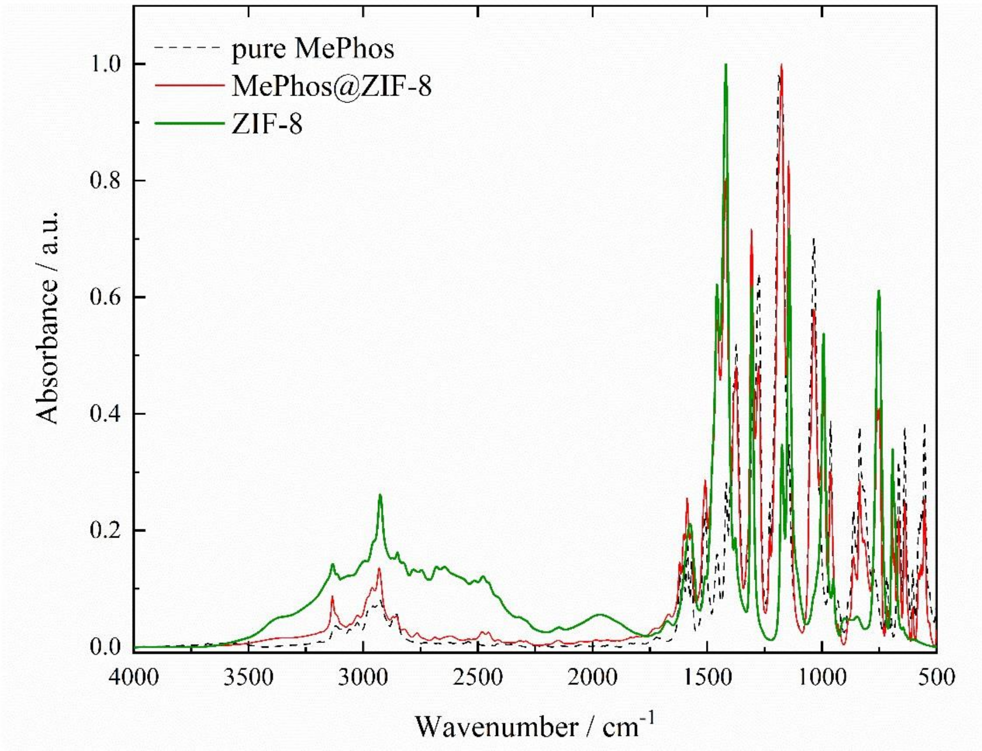

Figure S42. IR spectra of pure MePhos (dashed black line), MePhos@ZIF-8 (red line) and ZIF-8 (green line).

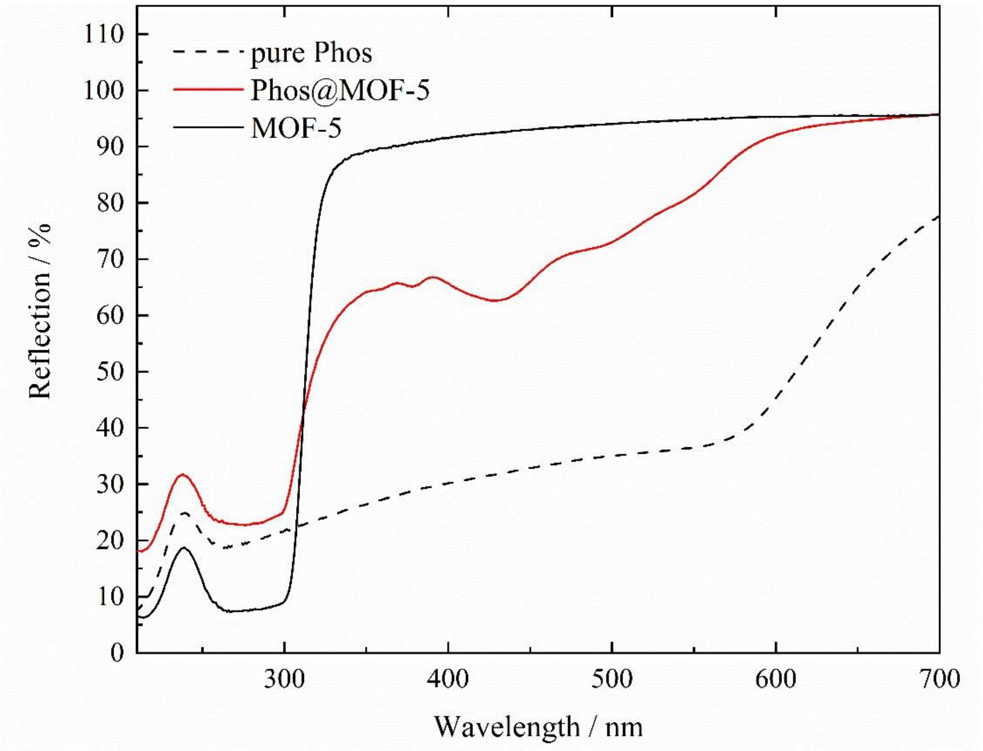

Figure S43. DRS spectra of pure Phos (dashed black line), Phos@MOF-5 (red line) and MOF-5 (black line).

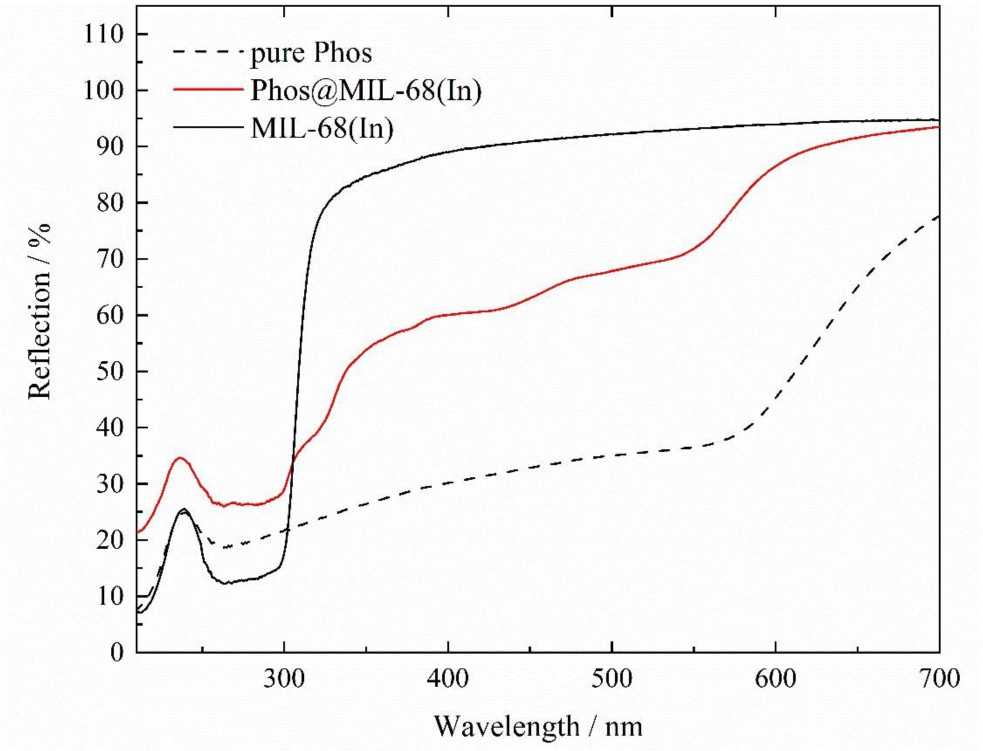

Figure S44. DRS spectra of pure Phos (dashed black line) and Phos@MIL-68(In) (red line).

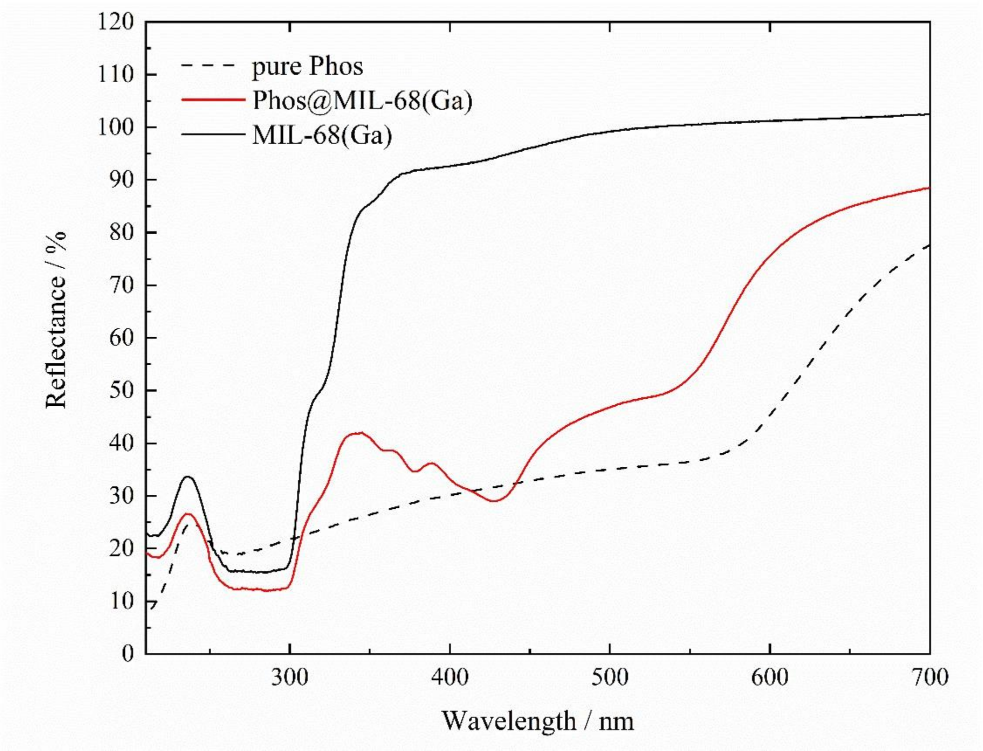

Figure S45. DRS spectra of pure Phos (dashed black line), Phos@MIL-68(Ga) (red line) and MIL-68(Ga) (black line).

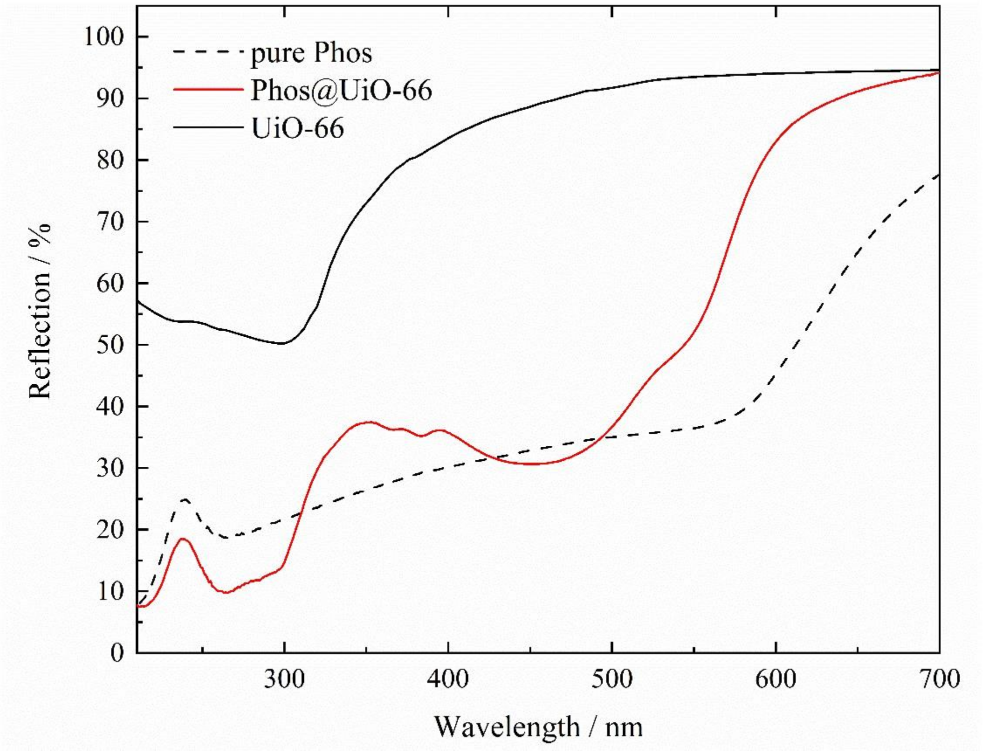

Figure S46. DRS spectra of pure Phos (dashed black line), Phos@UiO-66 (red line) and UiO-66 (black line).

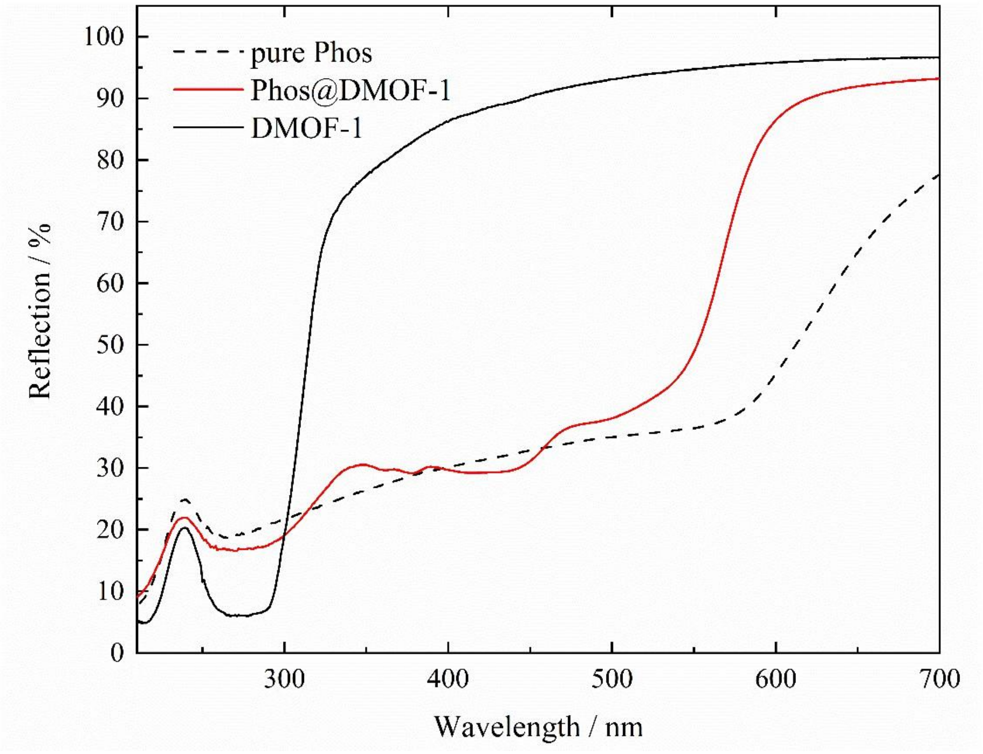

Figure S47. DRS spectra of pure Phos (dashed black line), Phos@DMOF-1 (red line) and DMOF-1 (black line).

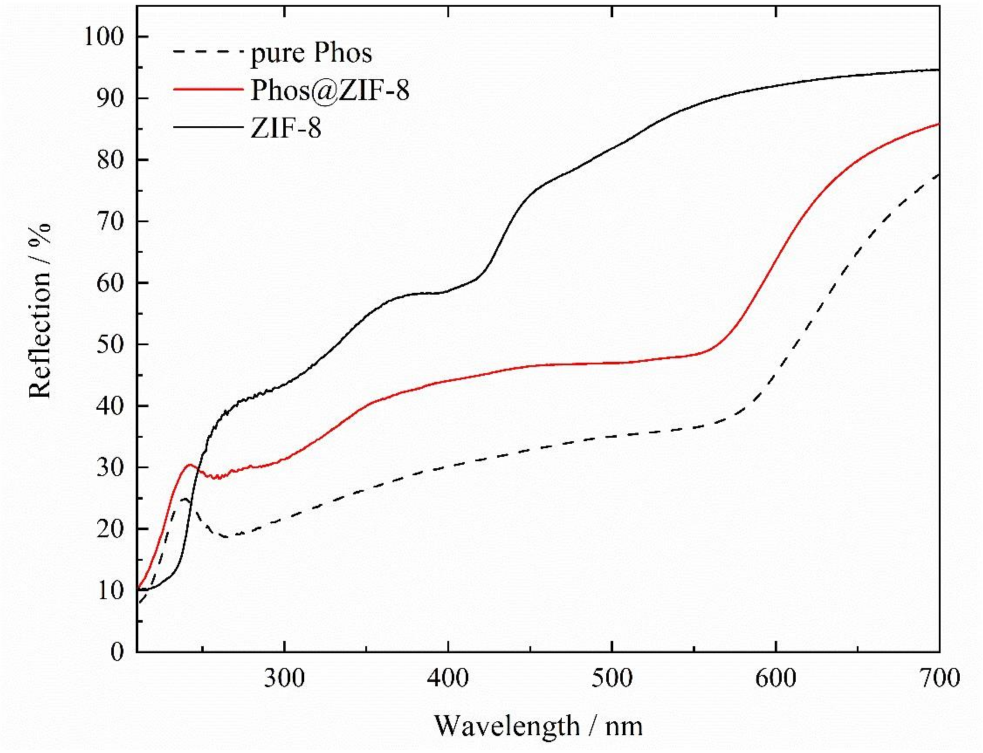

Figure S48. DRS spectra of pure Phos (dashed black line), Phos@ZIF-8 (red line) and ZIF-8 (black line).

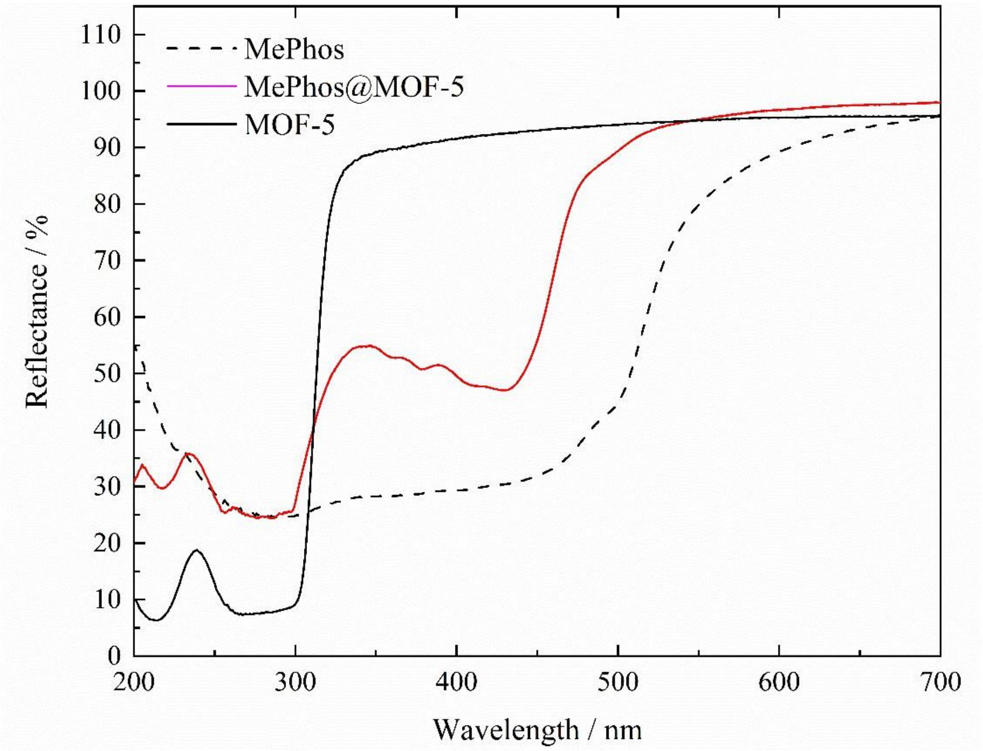

Figure S49. DRS spectra of pure MePhos (dashed black line), MePhos@MOF-5 (red line) and MOF-5 (black line).

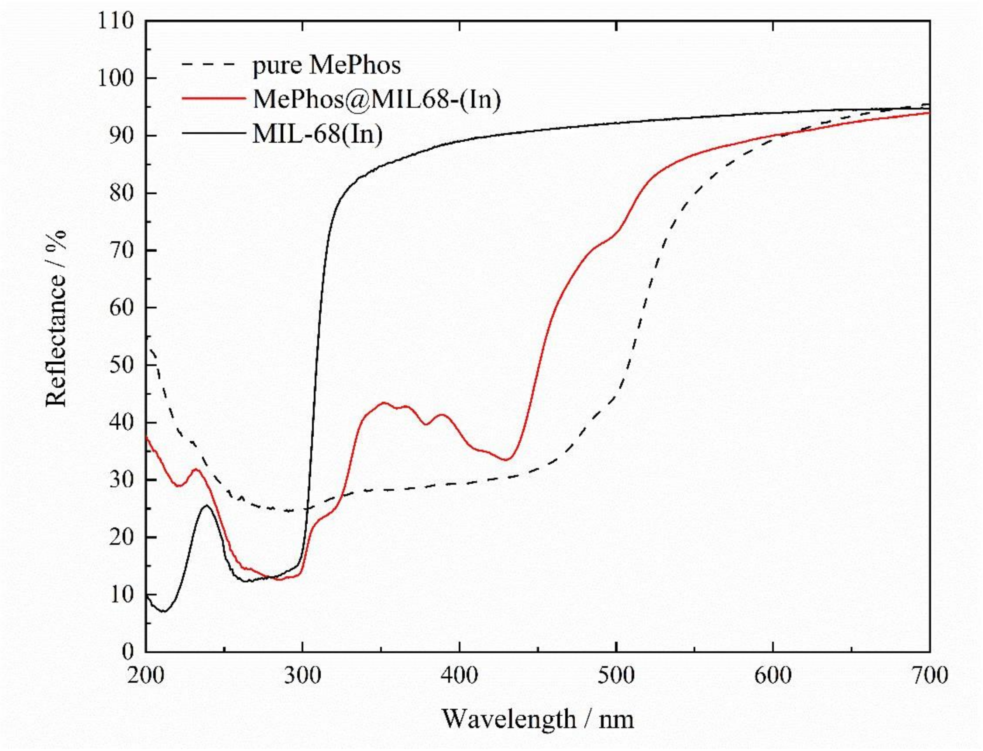

Figure S50. DRS spectra of pure MePhos (dashed black line), MePhos@MIL-68(In) (red line) and MIL-68(In) (black line).

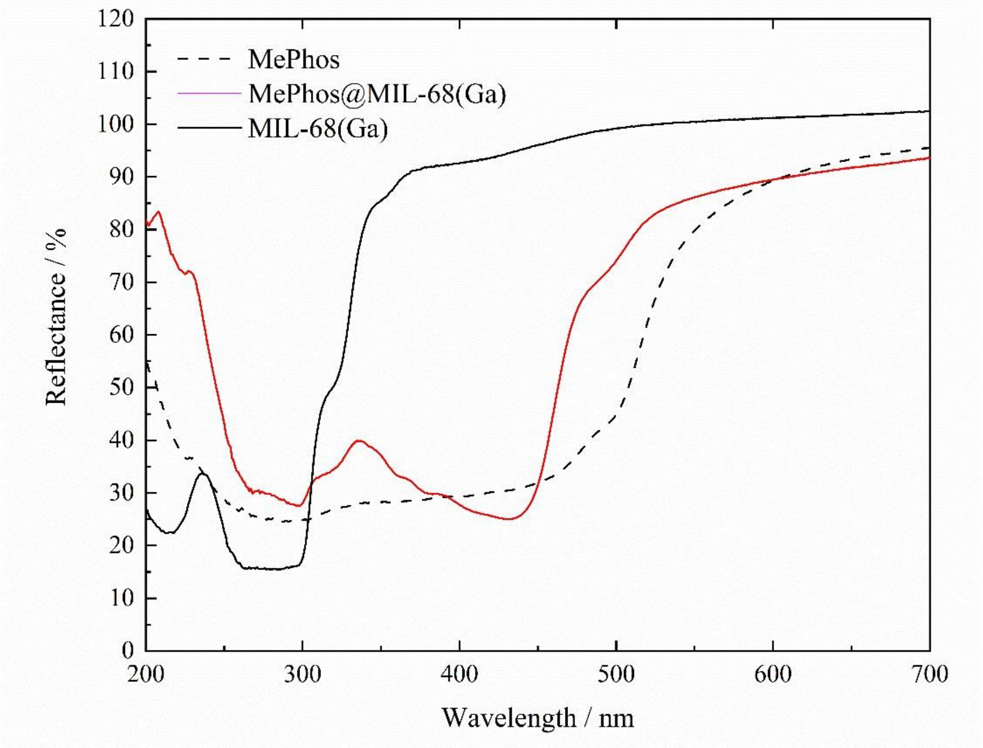

Figure S51. DRS spectra of pure MePhos (dashed black line), MePhos@MIL-68(Ga) (red line) and MIL-68(Ga) (black line).

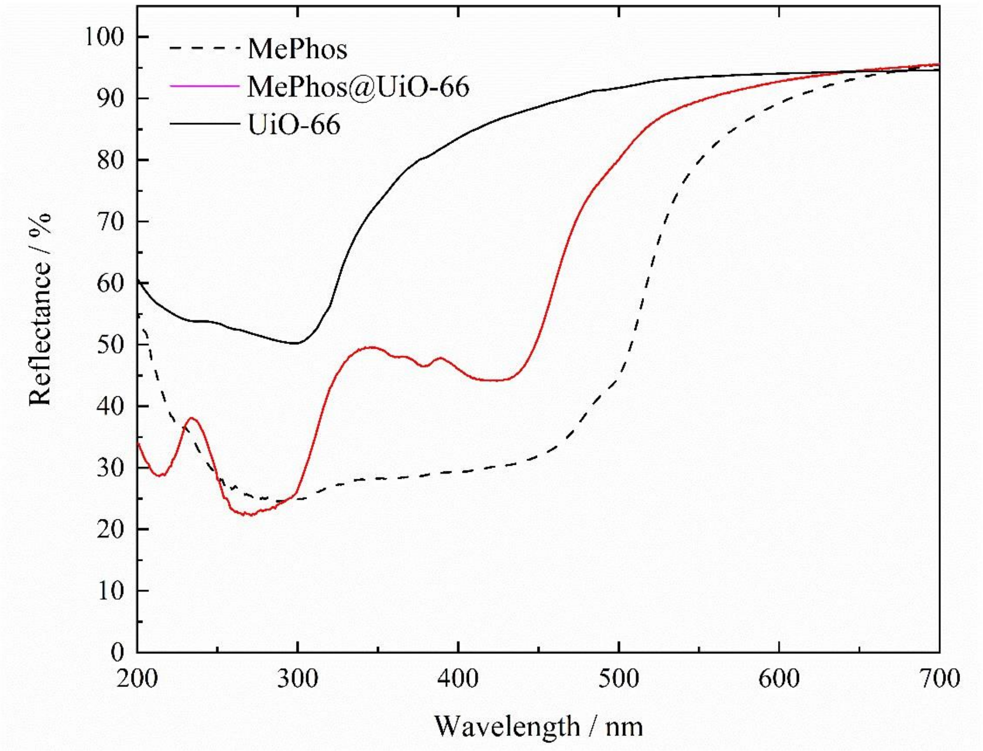

Figure S52. DRS spectra of pure MePhos (dashed black line), MePhos@UiO-66 (red line) and UiO-66 (black line).

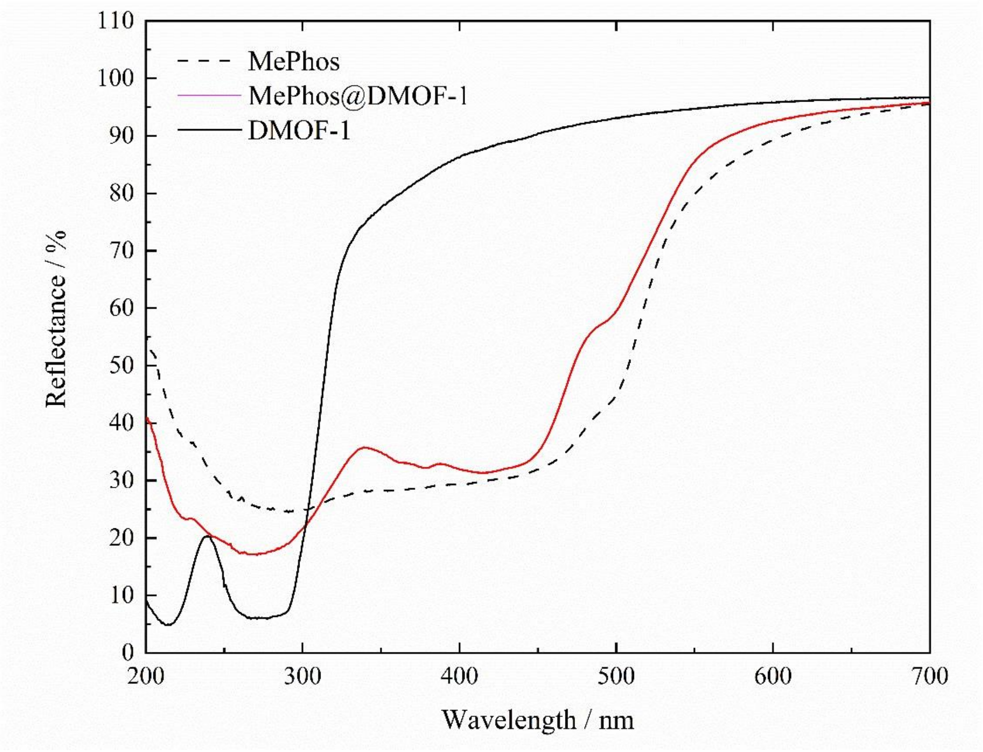

Figure S53. DRS spectra of pure MePhos (dashed black line), MePhos@DMOF-1 (red line) and DMOF-1 (black line).

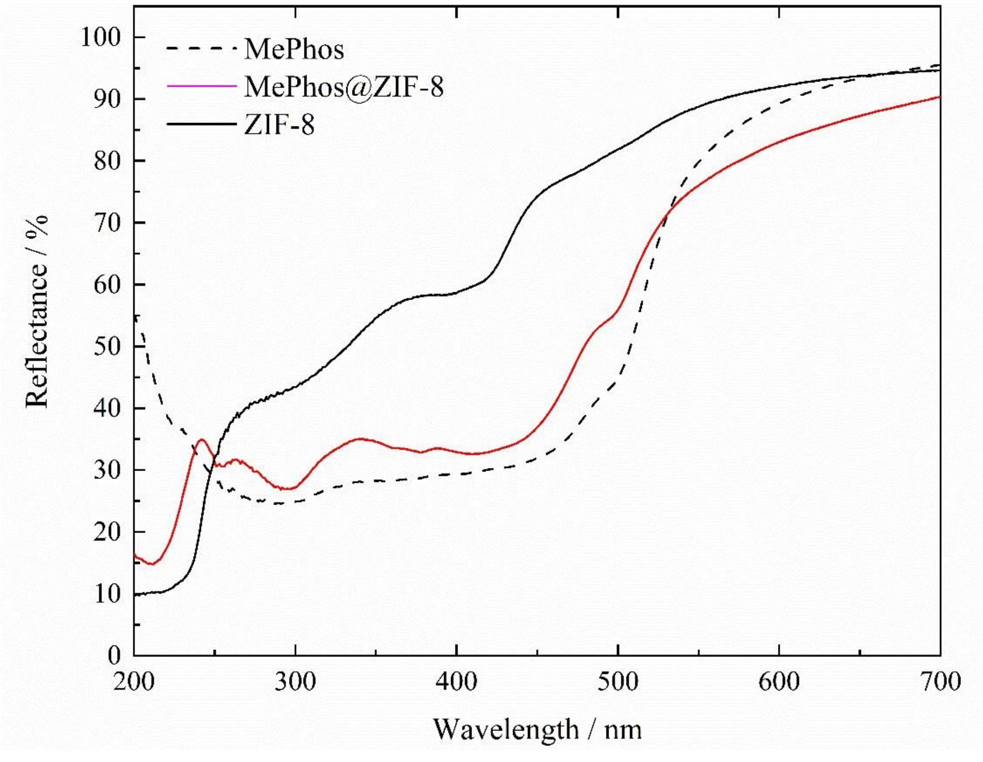

Figure S54. DRS spectra of pure MePhos (dashed black line), MePhos@ZIF-8 (red line) and ZIF-8 (black line).

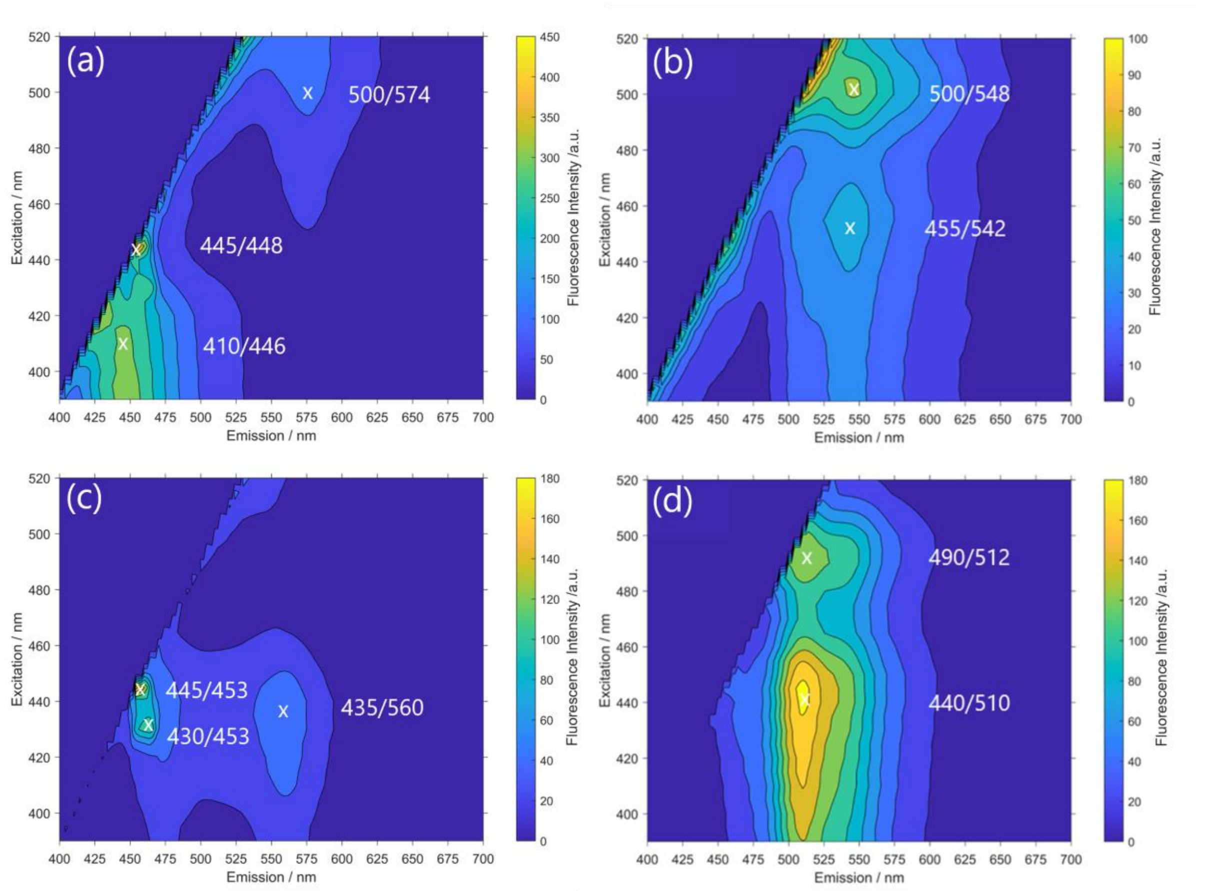

Figure S55. 2D-contour plot of emission excitation fluorescence intensity of (a) Phos@ZIF-8, (b) MePhos@ZIF-8,(c) Phos@MIL-68(Ga), (d) MePhos@MIL-68(Ga).

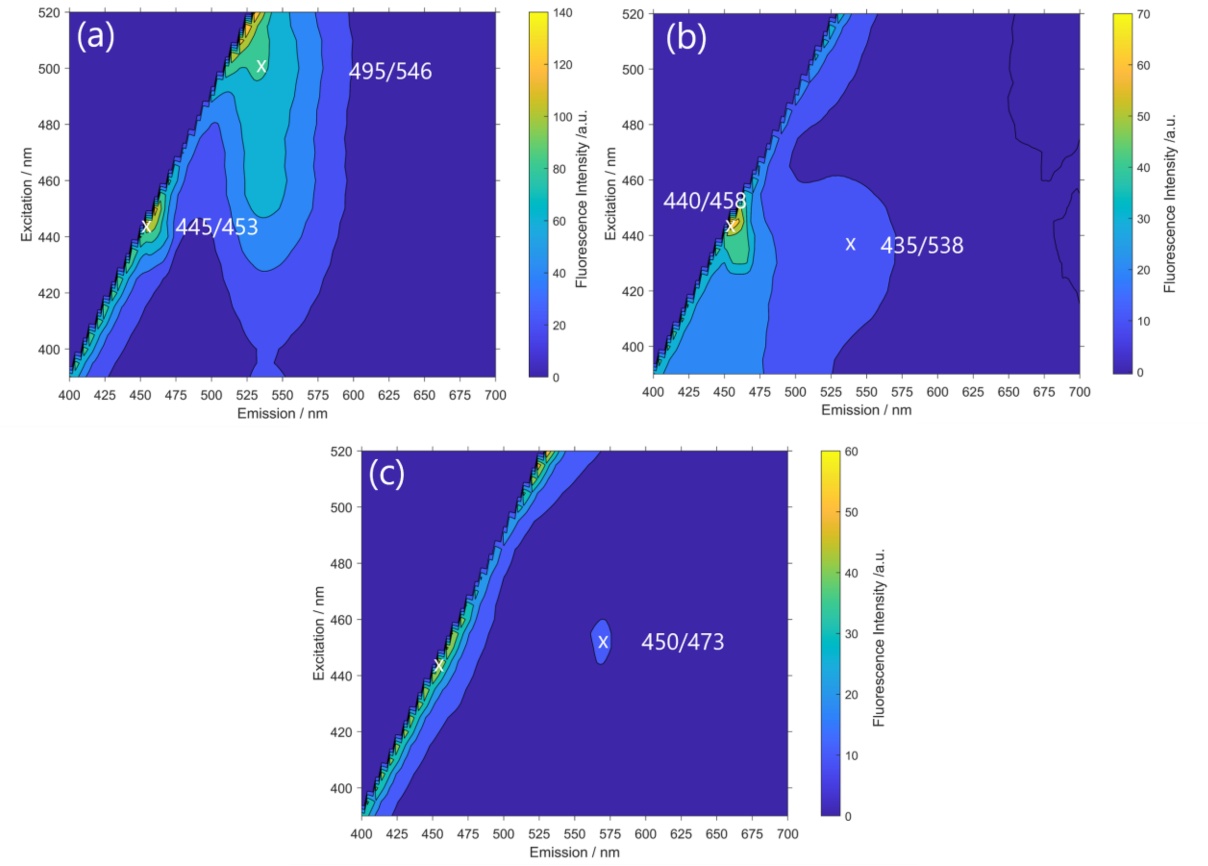

Figure S56. 2D-contour plot of emission excitation fluorescence intensity of (a) Phos@UiO-66, (b) Phos@MIL-68(In), (c) Phos@DMOF-1.

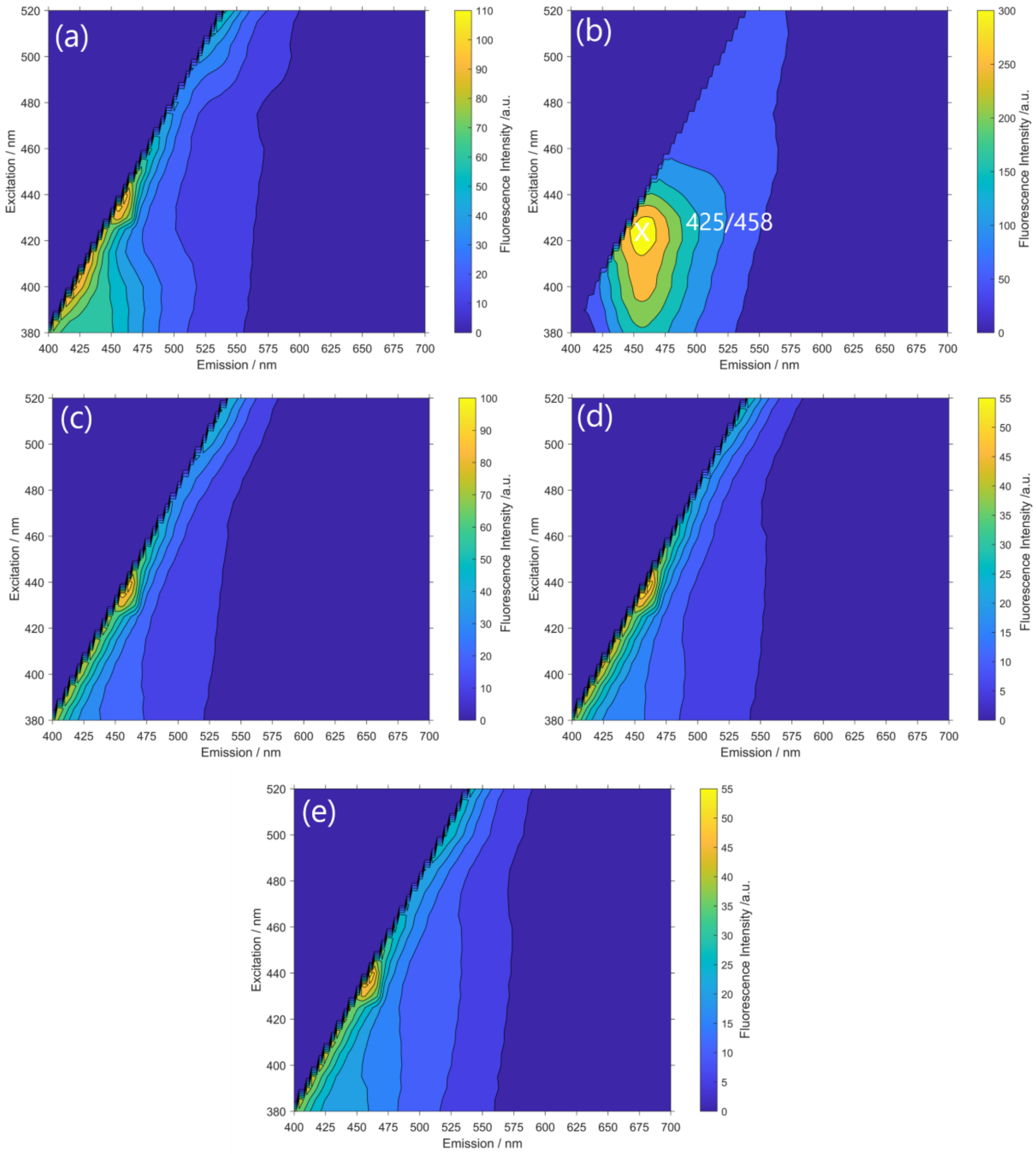

Figure S57. 2D-contour plot of emission excitation fluorescence intensity of pure MOFs. (a) MIL-68(In), (b) ZIF-8, (c) DMOF-1, (d) MIL-68(Ga), (e) UiO-66.

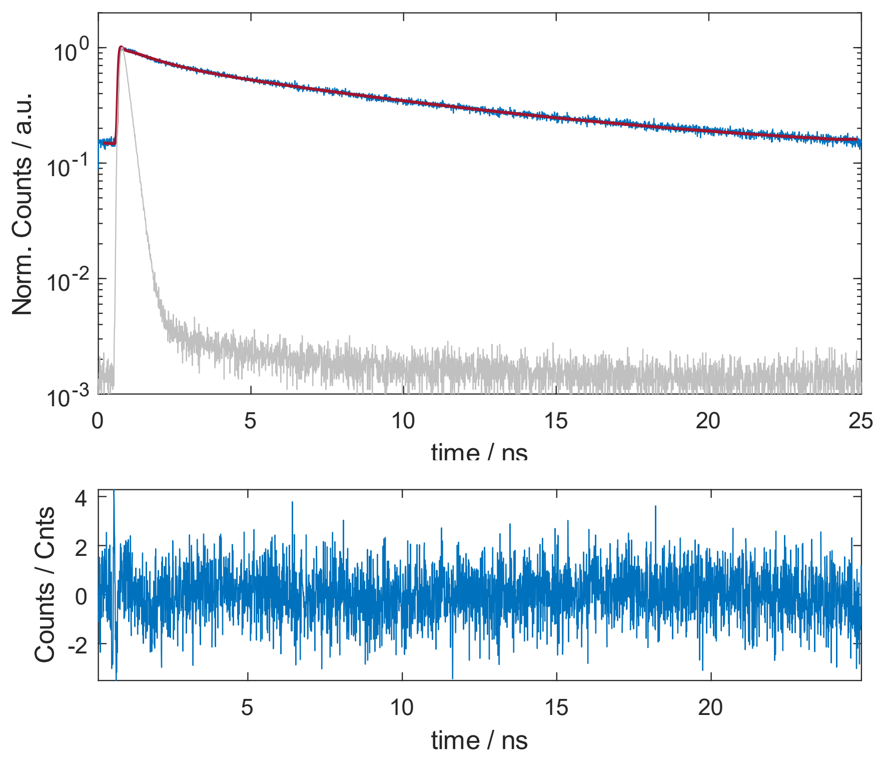

Figure S58. TCSPC histogram of MePhos@MIL-68(In) at λ_{exc} = 405 nm with λ_{det} = 500-550 nm.

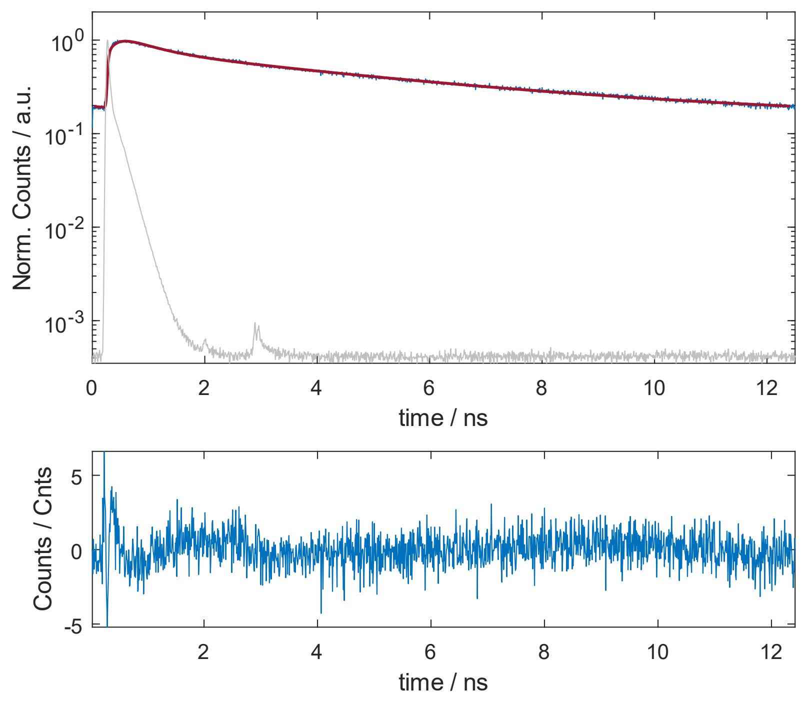

Figure S59. TCSPC histogram of MePhos@MIL-68(In) at λ_{exc} = 490 nm with λ_{det} = 525-645 nm.

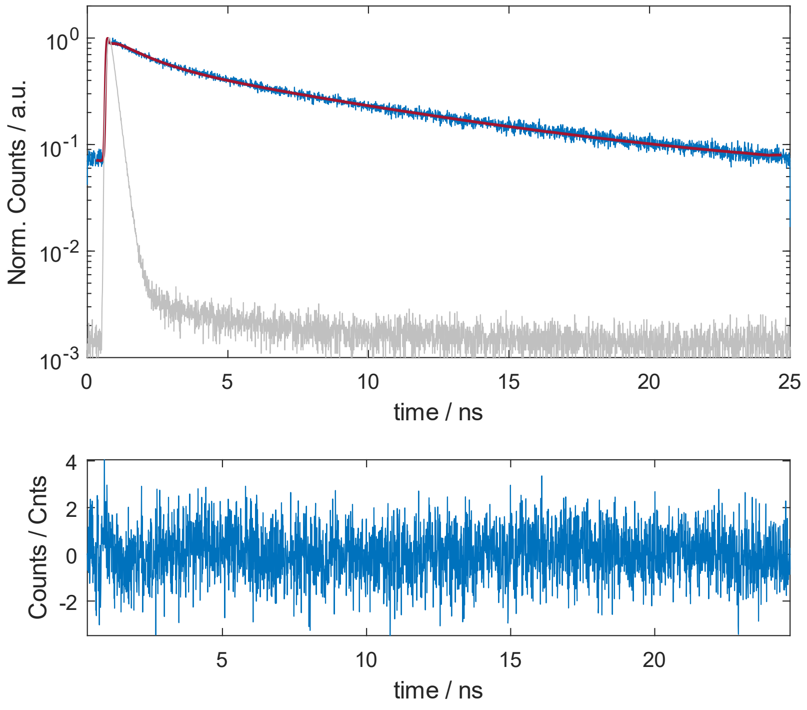

Figure S60. TCSPC histogram of MePhos@ZIF-8 at λ_{exc} = 405 nm with λ_{det} = 500-550 nm.

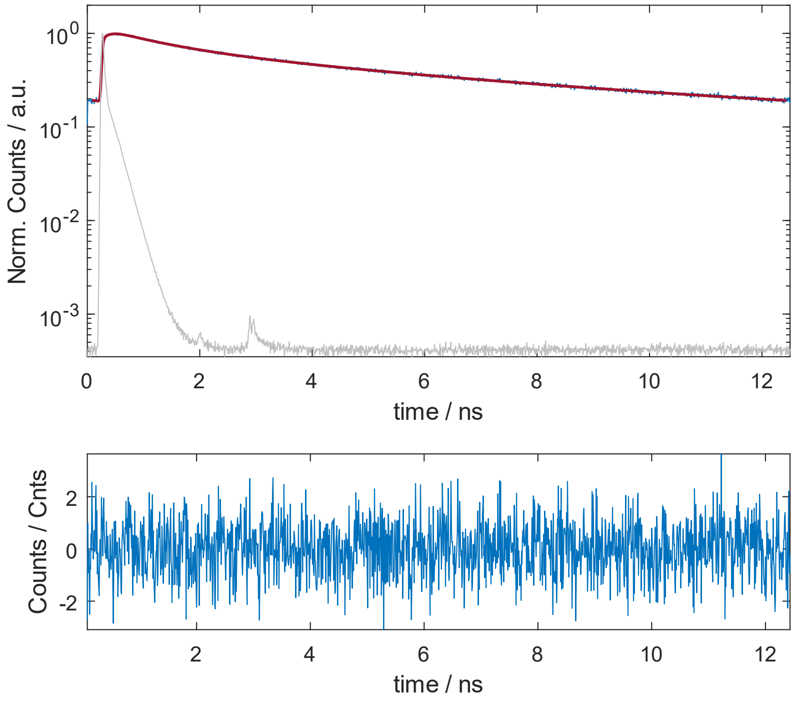

Figure S61. TCSPC histogram of MePhos@ZIF-8 at λ_{exc} = 490 nm with λ_{det} = 525-645 nm.

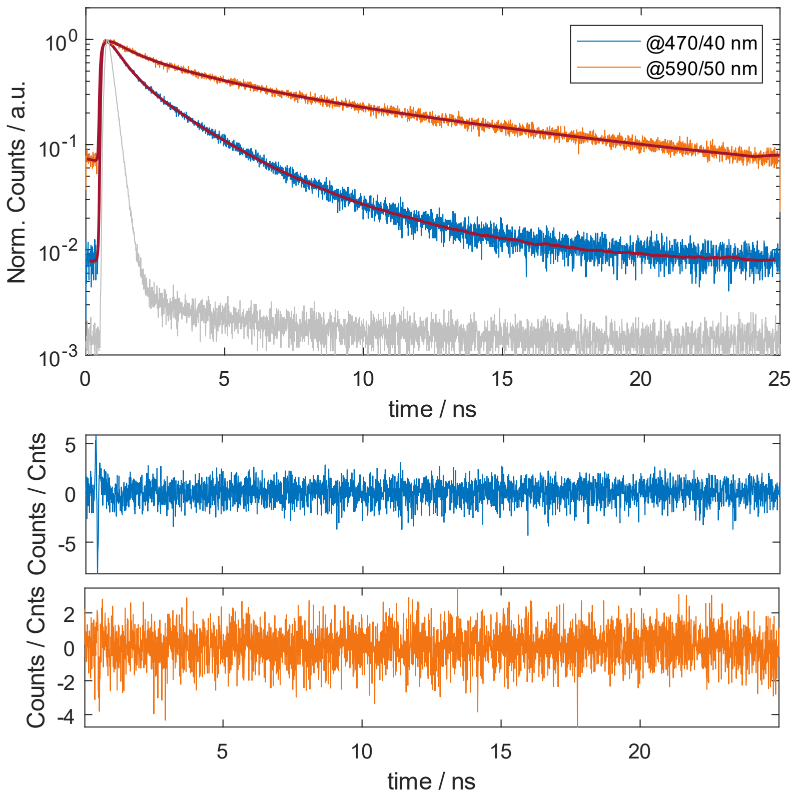

Figure S62. TCSPC histogram of MePhos@DMOF-1 at λ_{exc} = 405 nm with λ_{det} = 450-490 nm (blue) and λ_{det} = 565-615 nm (orange).

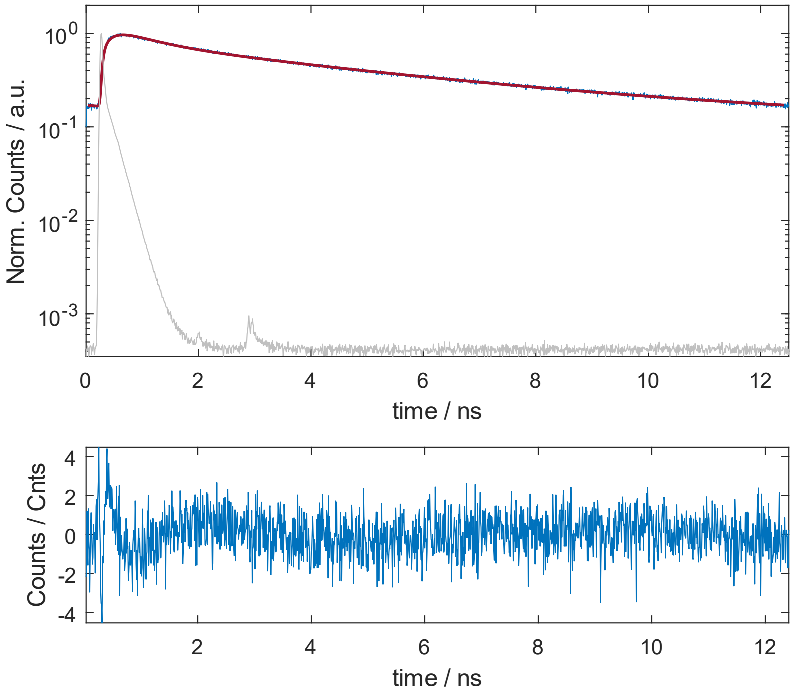

Figure S63. TCSPC histogram of MePhos@DMOF-1 at λ_{exc} = 490 nm with λ_{det} = 525-645 nm.

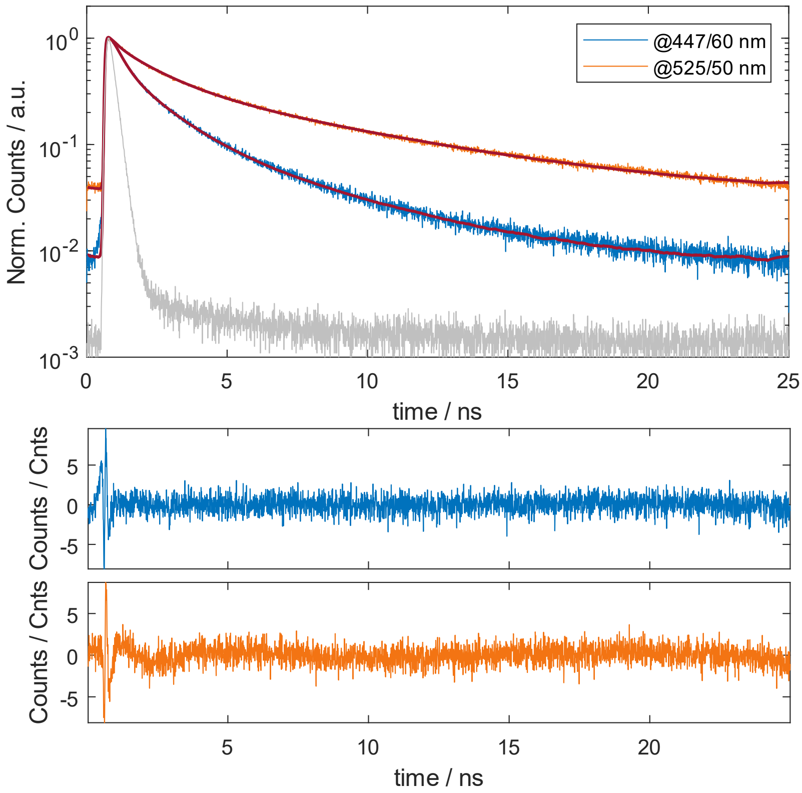

Figure S64. TCSPC histogram of MePhos@UiO-66 at λ_{exc} = 405 nm with λ_{det} = 417-477 nm (blue) and λ_{det} = 500-550 nm (orange).

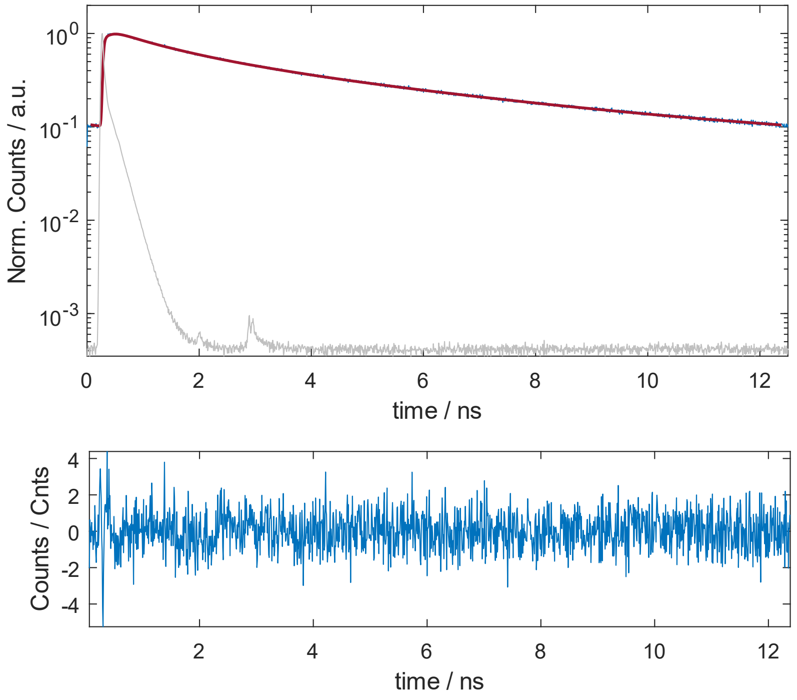

Figure S65. TCSPC histogram of MePhos@UiO-66 at λ_{exc} = 490 nm with λ_{det} = 525-645 nm.

Optical photostability. An LED chamber was used for photostability experiments. The samples were irradiated by a 450 nm LED (UHP-T-450-EP and UHP-T-LED-Controller, Prizmatix) with 64 mW cm^{-2} for up to 180 min. Before and after radiation, the fluorescence intensities were measured.

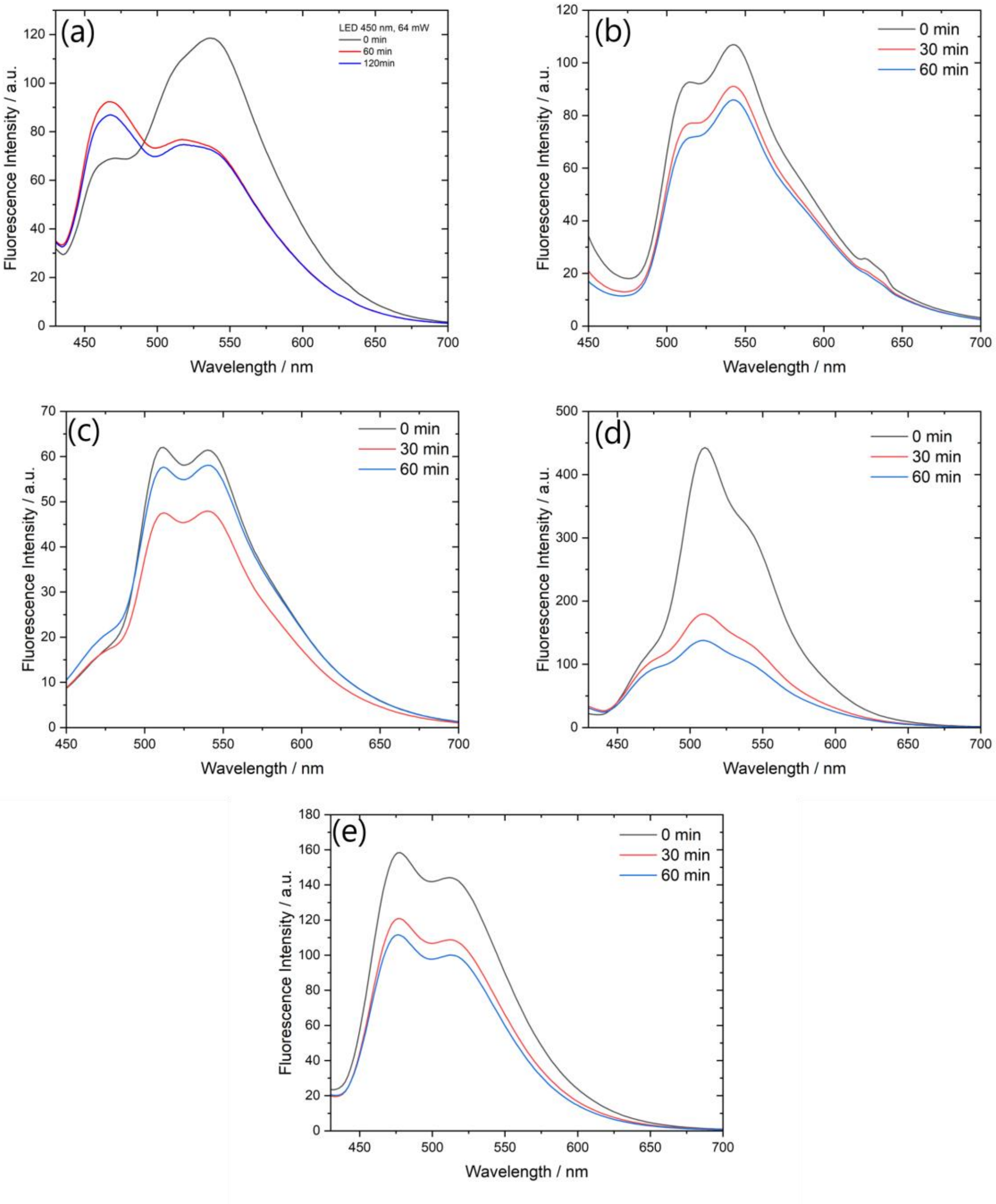

Figure S66. Emission spectra at λ_{exc}= 420 nm, before and after irradiation with 450 nm LED 64 mW cm^{-2}. (a) MePhos@MIL-68(In), (b) MePhos@ZIF-8, (c) MePhos@DMOF-1, (d) MePhos@MIL-68(Ga), (e) MePhos@UiO-66.

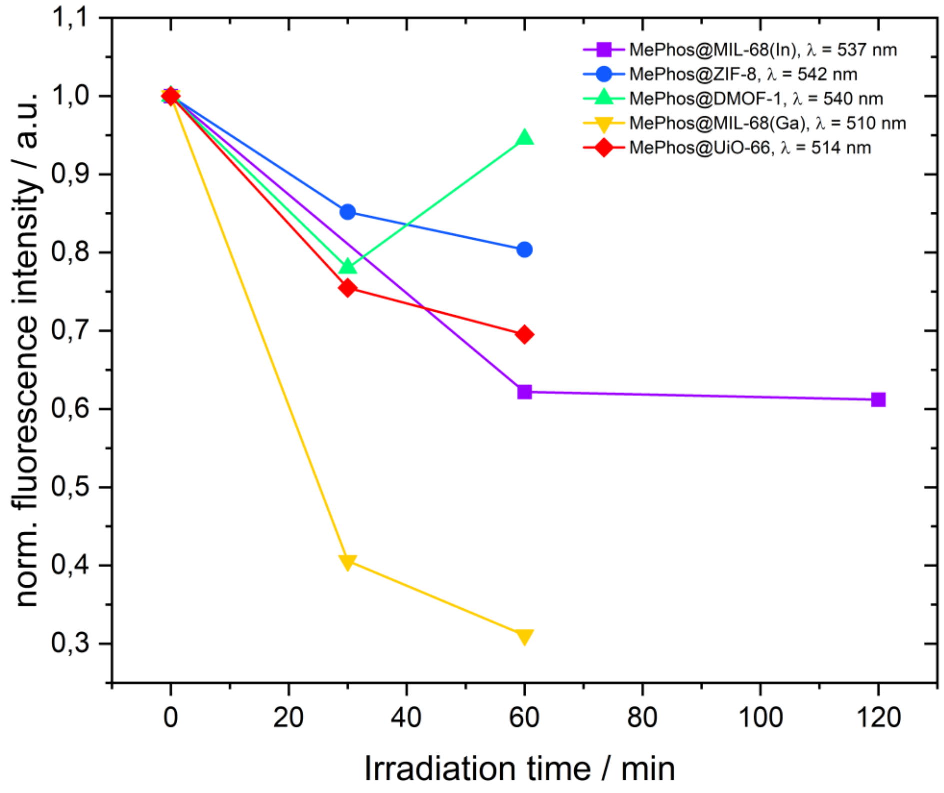

Figure S67. Correlation between fluorescence intensity and irradiation time at wavelength maximum.

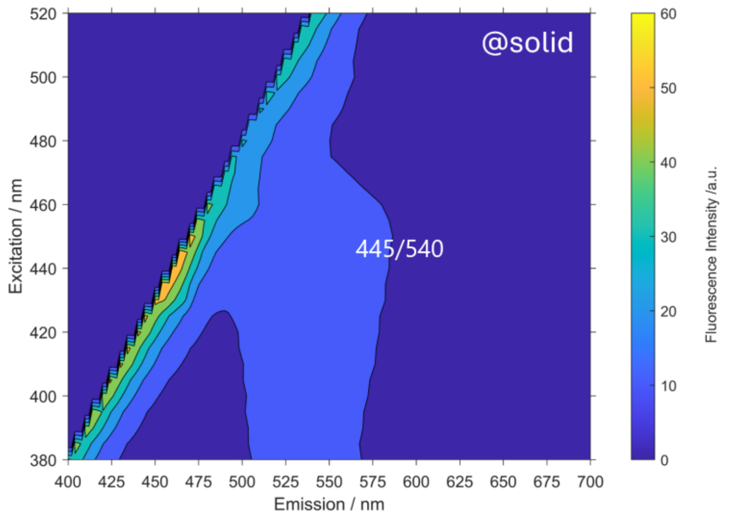

Figure S68. 2D-contour plot of emission excitation fluorescence intensity of solid MePhos after irradiation of 60 min with a 450 nm LED, 64 mWcm^{-2}.

Table S1. Weighed-in masses, quantities in mol, and temperatures for the gas phase synthesis of the Phos@MOF systems.

	**MOF-5**	**MIL-68(In)**	**MIL-68(Ga)**
m_{Host} / mg	60	60	60
m_{Guest} / mg	6.85	17.8	21.0
n_{Host} /mol	$7.80 \cdot 10^{-5}$	$2.03 \cdot 10^{-4}$	$2.39 \cdot 10^{-4}$
n_{Guest} / mol	$9.75 \cdot 10^{-6}$	$2.54 \cdot 10^{-5}$	$2.99 \cdot 10^{-5}$
T / °C	80	80	70

Table S2. Weighed-in masses, quantities in mol, and temperatures for the gas phase synthesis of the Phos@MOF systems.

	**ZIF-8**	**DMOF-1**	**UiO-66**
m_{Host} / mg	60	60	60
m_{Guest} / mg	23.2	9.23	3.16
n_{Host} /mol	$2.64 \cdot 10^{-4}$	$1.05 \cdot 10^{-4}$	$3.60 \cdot 10^{-5}$
n_{Guest} / mol	$3.30 \cdot 10^{-5}$	$1.31 \cdot 10^{-5}$	$4.50 \cdot 10^{-6}$
T / °C	80	80	70

Table S3. Weighed-in masses, quantities in mol, and temperatures for the gas phase synthesis of the MePhos@MOF systems.

	**MOF-5**	**MIL-68(In)**	**MIL-68(Ga)**
m_{Host} / mg	60	60	60
m_{Guest} / mg	7.0	18.2	21.4
n_{Host} /mol	$7.80 \cdot 10^{-5}$	$2.03 \cdot 10^{-4}$	$2.39 \cdot 10^{-4}$
n_{Guest} / mol	$9.75 \cdot 10^{-6}$	$2.54 \cdot 10^{-5}$	$2.99 \cdot 10^{-5}$
T / °C	100	120	110

Table S4. Weighed-in masses, quantities in mol, and temperatures for the gas phase synthesis of the MePhos@MOF systems.

	**ZIF-8**	**DMOF-1**	**UiO-66**
m_{Host} / mg	60	60	60
m_{Guest} / mg	23.7	9.41	3.23
n_{Host} /mol	$2.64 \cdot 10^{-4}$	$1.05 \cdot 10^{-4}$	$3.60 \cdot 10^{-5}$
n_{Guest} / mol	$3.30 \cdot 10^{-5}$	$1.31 \cdot 10^{-5}$	$4.50 \cdot 10^{-6}$
T / °C	100	100	100

Composition determination *via* NMR. For all compounds except of **8** and **18**, NMR measurements were conducted to determine the composition of the Phos@MOF and MePhos@MOF hybrid systems. For this, the characteristic proton signals of both the MOF and

the Phos and MePhos, respectively, were integrated and related to each other. In detail, the following signals were chosen, which are listed in the following Table S7:

Table S5. Relevant ^{1}H NMR signals of Phos and MePhos as well as the different MOF hosts used for the determination of composition *via* NMR.

**compound**	**composition**	**^{1}H signal / ppm**	**Number of protons**
Phos	$C_{22}H_{15}F_9O_{10}S_3$	9.16	1
MePhos	$C_{23}H_{17}F_9O_{10}S_3$	9.19 (s)	1
MOF-5	$Zn_4O(C_8H_4O_2)_3$	8 (s)	12
MIL-68(In)	$In(OH)(C_8H_4O_2)$	8 (s)	4
MIL-68(Ga)	$Ga(OH)(C_8H_4O_2)$	8 (s)	4
ZIF-8	$ZnC_6H_6N_4$	7.48 (s)	2
DMOF-1	$Zn_2(C_8H_4O_2)_2$DABCO	8 (s)	8

Composition determination *via* XPS. For compounds **8** and **18**, XPS measurements were conducted to determine the composition of the Phos@UiO-66 and MePhos@UiO-66 hybrid systems. The calculations for the fits are listed in Tables S7. For Phos@UiO-66, the obtained value is higher than the weighed in ratios. As previously reported, UiO-66 systems tend to show higher guest values, as those position themselves near to the metal-cluster and, therefore, shield the metal cation. As a result, the Zr peaks are lower in intensity, and consequently, the obtained Phos value is higher.

Table S6. Relevant values for the determination of composition of (Me)Phos@UiO-66 materials by XPS.

	**Phos@UiO-66 (7)**	**MePhos@UiO-66 (16)**
Peak area for fluorine	842	546
RSF factor of fluorine	4.71 (F 1s)	4.71 (F 1s)
Fluorine atoms per (Me)Phos molecule	9	9
Peak area for the respective metal	2134	2174
RSF factor of the respective metal	4.17 (Zr $3d_{5/2}$)	4.17 (Zr $3d_{5/2}$)
Metal atoms per formula unit of the respective metal	6 ($Zr_6O_4(OH)_4(BDC)_6$)	6 ($Zr_6O_4(OH)_4(BDC)_6$)
**n((Me)Phos) : n(MOF)**	**0.24 : 1**	**0.16 : 1**

Table S7. Fluorescence lifetimes of the MePhos@MOF systems.

	Sample	τ_{fl} / ns	$\Delta\tau$ / ns	A /%	$\varnothing\tau_{fl}$ / ns
Laser λ_{exc} = 405 nm	**MePhos@MIL-68(In) (8)**	9.06	0.36	69	3.28 [a]
		1.11	0.26	31	
	**MePhos@ZIF-8(11)**	8.26	0.25	56	2.58 [a]
		1.25	0.11	44	
	**MePhos@DMOF-1(12)**	4.33	0.18	10	0.43 [b]
		1.84	0.07	35	
		0.43	0.02	55	
		10.80	0.56	35	1.96 [d]
		2.64	0.15	36	
		0.54	0.03	29	
	**MePhos@MIL-68(Ga) (9)**	6.80	0.02	100	6.80 [a]
	**MePhos@UiO-66 (10)**	4.73	0.08	9	0.30 [c]
		1.41	0.03	29	
		0.29	0.01	62	
		6.96	0.19	21	0.79 [a]
		1.54	0.08	30	
		0.17	0.03	48	
	**MeBF96 (l)**	3.94	0.0068	100	3.94 [b]
Laser λ_{exc} = 490 nm	**MePhos@MIL-68(In) (8)**	5.02	0.09	57	1.55 [e]
		0.53	0.03	43	
	**MePhos@ZIF-8(11)**	6.57	0.34	37	0.94 [e]
		1.19	0.07	26	
		0.14	0.01	37	
	**MePhos@DMOF-1(12)**	5.04	0.09	62	1.69 [e]
		0.67	0.03	38	
	**MePhos@MIL-68(Ga) (9)**	5.02	0.08	76	1.96 [e]
		0.43	0.05	24	
	**MePhos@UiO-66 (10)**	4.90	0.11	35	0.70 [e]
		1.22	0.05	27	
		0.15	0.01	38	
	**MeBF96 (s)**	5.79	0.25	54	1.77 [e]
		0.866	0.058	46	

λ_{det} / nm: [a] 525/50, [b] 470/40, [c] 447/60, [d] 590/50, [e] 585/120.
